# Time course gene expression data in colon of mice after exposure to food-grade E171

**DOI:** 10.1016/j.dib.2017.11.067

**Published:** 2017-11-22

**Authors:** Héloïse Proquin, Marlon J. Jetten, Marloes C.M. Jonkhout, Luis G. Garduño-Balderas, Jacob J. Briedé, Theo M. de Kok, Yolanda I. Chirino, Henk van Loveren

**Affiliations:** a Department of Toxicogenomics, GROW Institute of Oncology and Developmental Biology, Maastricht University, The Netherlands; b Laboratorio de Carcinogénesis y Toxicología, Unidad de Biomedicina, FES-Iztacala, UNAM, Estado de México, Mexico; c IUF-Leibniz Research Institute for Environmental Medicine, Auf'm Hennekamp 50, 40225 DE Düsseldorf, Germany

## Abstract

We investigated gene expression responses in BALB/c mice exposed by gavage to 5 mg/kg bw/day of E171 for 2, 7, 14 and 21 days. Food additive E171 (titanium dioxide) has been shown to induce oxidative stress and DNA damage *in vitro* as well as facilitating growth of colorectal tumours *in vivo*. Full genome expression changes of the colon of mice were investigated by using Agilent SurePrint G3 mouse Gene exp 60kv2 microarrays slides. The data presented in this DiB include all differentially expressed for each time point with EntrezGeneID, gene symbols, gene names and Log2FC as well as genes included in pathways after over-representation analysis in ConsensusPathDataBase. The functions of these genes in relation to the colon were described in our associated article (Proquin et al., 2017 in press) [1]. Raw and normalized gene expression data are available through NCBI GEO (GEO accession: GSE92563).

**Specifications Table**

**Table:** 

Subject area	*Biology*
More specific subject area	*Food Toxicogenomics*
Type of data	*Tables*
How data was acquired	*Agilent SurePrint G3 mouse Gene exp 60kv2 microarrays*
Data format	*Differentially expressed genes (DEG) and DEG in pathways (after over-representation analysis in ConsensusPathDataBase) with Log2FC per time point*
Experimental factors	*BALB/c mice were exposed by gavage to 5 *mg/kg *bw/day of E171 for 2, 7, 14 and 21 days*
Experimental features	*Data from each time point of exposure was corrected by its time-matched control*
Data source location	*Department of Toxicogenomics, Maastricht University, the Netherlands*
Data accessibility	*Raw and normalized gene expression data are available through NCBI GEO (GEO accession:*GSE92563*)*

**Value of the data**

• The DEG obtained after exposure to E171 in colon of mice can serve as a benchmark to validate functional measurements such as metabolomics and proteomics.

• These data can be compared to further advance time series studies in other organs after E171 exposure.
• All the differentially expressed genes that could not be linked directly to pathways are of major interest for further studies since these might be related to yet unknown (biological) processes activated by exposure to E171.

## 1 Data

Titanium dioxide, referred to as E171, is used as a colouring agent in various types of food like sweets, cookies, coffee creamers, and salad dressings [1], [2]. To establish molecular responses that may relate to potential health effects in the colon, BALB/c mouse were intragastrically exposed to 5 mg/kg bw/day of E171 for 2, 7, 14, and 21 days. Microarray analyses of the colon of the mice showed the effects of E171 exposure on the whole transcriptome. The number of DEG was 417 after 2 days of exposure (Table 1), 971 after 7 days of exposure (Table 2), 1512 after 14 days (Table 3), and 229 after 21 days of exposure (Table 4). The data shows that exposure to E171 affects the expression of genes which are involved in oxidative stress, immune response, DNA repair, development of cancer for instance colon cancer, and regulation of GPCR/olfactory and serotonin receptors (Table 5, Table 6, Table 7, Table 8). A relatively large proportion of the DEG could not be linked to known molecular pathways which indicate that E171 is affecting the biological response beyond currently known processes (Figure 3, in Ref. [3]).

**Table 1:** Differentially expressed genes (DEG) in the colon of mice after 2 days of exposure to E171.

**Entrez gene ID**	**Gene symbol**	**Gene name**	**Log2FC**
11434	Acr	acrosin prepropeptide	−0.94033
11498	Adam4	a disintegrin and metallopeptidase domain 4	0.725974
12064	Bdnf	brain derived neurotrophic factor	−1.07104
12154	Bmp10	bone morphogenetic protein 10	−0.79709
12575	Cdkn1a	cyclin-dependent kinase inhibitor 1A (P21)	0.728624
12965	Crygb	crystallin, gamma B	−0.98104
13078	Cyp1b1	cytochrome P450, family 1, subfamily b, polypeptide 1	2.613566
13119	Cyp4a14	cytochrome P450, family 4, subfamily a, polypeptide 14	−0.85899
13172	Dbx1	developing brain homeobox 1	1.557234
13411	Dnah11	dynein, axonemal, heavy chain 11	1.85893
13476	Reep5	receptor accessory protein 5	0.737949
13492	Drd5	dopamine receptor D5	−1.0558
14380	G6pd2	glucose-6-phosphate dehydrogenase 2	0.842643
14599	Gh	growth hormone	−1.02848
14602	Ghrhr	growth hormone releasing hormone receptor	−1.05079
14610	Gja10	gap junction protein, alpha 10	−0.92558
14676	Gna15	guanine nucleotide binding protein, alpha 15	−0.86407
14764	Ptgdr2	prostaglandin D2 receptor 2	−1.13784
14997	H2-M9	histocompatibility 2, M region locus 9	−2.12823
15132	Hbb-bh1	hemoglobin Z, beta-like embryonic chain	−1.10259
15202	Hemt1	hematopoietic cell transcript 1	−0.61414
15502	Dnaja1	DnaJ (Hsp40) homolog, subfamily A, member 1	−0.89922
15505	Hsph1	heat shock 105kDa/110kDa protein 1	−1.21534
15511	Hspa1b	heat shock protein 1B	−2.10535
16150	Ikbkb	inhibitor of kappaB kinase beta	0.601827
16639	Klra8	killer cell lectin-like receptor, subfamily A, member 8	1.072256
17139	Magea3	melanoma antigen, family A, 3	−0.67136
17684	Cited2	Cbp/p300-interacting transactivator, with Glu/Asp-rich carboxy-terminal domain, 2	0.586504
17873	Gadd45b	growth arrest and DNA-damage-inducible 45 beta	0.779545
17928	Myog	myogenin	−1.80681
18013	Neurod2	neurogenic differentiation 2	0.814139
18331	Olfr32	olfactory receptor 32	−0.96808
19127	Prop1	paired like homeodomain factor 1	−0.79344
19206	Ptch1	patched homolog 1	0.672454
19661	Rbp3	retinol binding protein 3, interstitial	1.042255
20302	Ccl3	chemokine (C-C motif) ligand 3	2.147125
20430	Cyfip1	cytoplasmic FMR1 interacting protein 1	0.689064
20445	St6galnac1	ST6 (alpha-N-acetyl-neuraminyl-2,3-beta-galactosyl-1,3)-N-acetylgalactosaminide alpha-2,6-sialyltransferase 1	−0.64032
20539	Slc7a5	solute carrier family 7 (cationic amino acid transporter, y+ system), member 5	−0.76003
20612	Siglec1	sialic acid binding Ig-like lectin 1, sialoadhesin	0.668819
20776	Tmie	transmembrane inner ear	0.849596
21784	Tff1	trefoil factor 1	−0.94951
21924	Tnnc1	troponin C, cardiac/slow skeletal	−1.05397
22213	Ube2g2	ubiquitin-conjugating enzyme E2G 2	−0.6056
22599	Slc6a20b	solute carrier family 6 (neurotransmitter transporter), member 20B	−1.60112
22691	Zscan2	zinc finger and SCAN domain containing 2	0.805138
27412	Peg12	paternally expressed 12	0.62484
30959	Ddx25	DEAD (Asp-Glu-Ala-Asp) box polypeptide 25	−1.41324
51960	Kctd18	potassium channel tetramerisation domain containing 18	0.766087
52392	D1Ertd622e	DNA segment, Chr 1, ERATO Doi 622, expressed	−0.71485
53311	Mybph	myosin binding protein H	−1.5695
53871	Pkd2l2	polycystic kidney disease 2-like 2	0.633894
54652	Cacna1f	calcium channel, voltage-dependent, alpha 1F subunit	0.882943
55981	Pigb	phosphatidylinositol glycan anchor biosynthesis, class B	0.646794
56293	Slc35g3	solute carrier family 35, member G3	−0.98906
56544	Vmn2r1	vomeronasal 2, receptor 1	1.00315
56747	Sez6l	seizure related 6 homolog like	−0.61524
57271	Olfr1509	olfactory receptor 1509	−0.71953
57355	BC051019	cDNA sequence BC051019	1.033251
57429	Sult5a1	sulfotransferase family 5A, member 1	−0.7115
57764	Ntn4	netrin 4	0.724402
57778	Fmnl1	formin-like 1	1.015557
58803	Pga5	pepsinogen 5, group I	−0.74806
65079	Rtn4r	reticulon 4 receptor	1.409669
66139	Tmem8c	transmembrane protein 8C	−0.6415
66184	Rps4l	ribosomal protein S4-like	−0.76974
66514	Asrgl1	asparaginase like 1	−0.6953
66547	2010203P06Rik	RIKEN cDNA 2010203P06 gene	−1.49869
66784	Fam187a	family with sequence similarity 187, member A	1.564028
66917	Chordc1	cysteine and histidine-rich domain (CHORD)-containing, zinc-binding protein 1	−0.67857
66933	1700025L06Rik	RIKEN cDNA 1700025L06 gene	0.83979
67261	2900005J15Rik	RIKEN cDNA 2900005J15 gene	0.664871
67316	1700037F03Rik	RIKEN cDNA 1700037F03 gene	0.997954
67349	1700086P04Rik	RIKEN cDNA 1700086P04 gene	0.841403
67548	4933424M12Rik	RIKEN cDNA 4933424M12 gene	−0.89088
67553	Gstcd	glutathione S-transferase, C-terminal domain containing	1.160582
67585	4930455J16Rik	RIKEN cDNA 4930455J16 gene	−1.27365
67603	Dusp6	dual specificity phosphatase 6	−0.65975
67610	Rspry1	ring finger and SPRY domain containing 1	−0.59499
67637	4930470P17Rik	RIKEN cDNA 4930470P17 gene	−1.41889
67685	Dyx1c1	dyslexia susceptibility 1 candidate 1 homolog (human)	0.596986
67914	Coq9	coenzyme Q9 homolog (yeast)	0.596446
68067	3010026O09Rik	RIKEN cDNA 3010026O09 gene	−1.08629
68175	4930591A17Rik	RIKEN cDNA 4930591A17 gene	−1.09635
68206	2900060N18Rik	RIKEN cDNA 2900060N18 gene	−0.84487
68208	1700039O17Rik	RIKEN cDNA 1700039O17 gene	1.044673
68348	Serpina1f	serine (or cysteine) peptidase inhibitor, clade A, member 1F	−0.90621
69241	Polr2d	polymerase (RNA) II (DNA directed) polypeptide D	−0.66069
69294	Cst13	cystatin 13	−1.32139
69307	Pxt1	peroxisomal, testis specific 1	−0.72362
69308	1700007P06Rik	RIKEN cDNA 1700007P06 gene	−0.76625
69384	Tmem89	transmembrane protein 89	1.226223
69439	Mroh4	maestro heat-like repeat family member 4	−0.96061
69732	2410018L13Rik	RIKEN cDNA 2410018L13 gene	0.624498
69851	2010007E15Rik	RIKEN cDNA 2010007E15 gene	1.098482
70248	Dazap1	DAZ associated protein 1	0.61928
70417	Megf10	multiple EGF-like-domains 10	−0.9536
70426	Tekt5	tektin 5	−0.6079
70448	Atad3aos	ATPase family, AAA domain containing 3A, opposite strand	0.771291
70868	4921515L22Rik	RIKEN cDNA 4921515L22 gene	−1.81913
70993	Prss54	protease, serine 54	−0.87092
71011	4933401B06Rik	GLE1 RNA export mediator pseudogene	0.707354
71296	Crnde	colorectal neoplasia differentially expressed (non-protein coding)	−0.82775
71766	Raver1	ribonucleoprotein, PTB-binding 1	0.836389
71873	2310003N18Rik	RIKEN cDNA 2310003N18 gene	−1.10333
71918	Zcchc24	zinc finger, CCHC domain containing 24	0.796492
71939	Apol6	apolipoprotein L 6	0.990353
72647	2700089I24Rik	RIKEN cDNA 2700089I24 gene	−0.84698
72753	2810442N19Rik	RIKEN cDNA 2810442N19 gene	−0.59812
72789	Veph1	ventricular zone expressed PH domain-containing 1	−0.82245
73335	1700047K16Rik	RIKEN cDNA 1700047K16 gene	1.993123
73353	Actrt2	actin-related protein T2	−1.00022
73472	Spata18	spermatogenesis associated 18	−0.87035
73603	Trp53tg5	transformation related protein 53 target 5	−0.82983
73795	4930405D01Rik	RIKEN cDNA 4930405D01 gene	−0.84615
73906	4833417C18Rik	RIKEN cDNA 4833417C18 gene	0.841393
74314	1700120G07Rik	RIKEN cDNA 1700120G07 gene	1.44201
75010	Tmbim7	transmembrane BAX inhibitor motif containing 7	−0.81651
75100	4930525D18Rik	RIKEN cDNA 4930525D18 gene	−1.07596
75120	4930509E22Rik	RIKEN cDNA 4930509E22 gene	−1.07432
75601	1810049I09Rik	RIKEN cDNA 1810049I09 gene	−1.47298
75802	4930458D05Rik	RIKEN cDNA 4930458D05 gene	0.666758
75878	4930579P08Rik	RIKEN cDNA 4930579P08 gene	−0.91714
75913	4930579G18Rik	RIKEN cDNA 4930579G18 gene	−0.98207
76432	2310001H17Rik	RIKEN cDNA 2310001H17 gene	0.823092
76511	2010004M13Rik	RIKEN cDNA 2010004M13 gene	−0.77825
76646	Wdr38	WD repeat domain 38	−0.68665
76668	Mdh1b	malate dehydrogenase 1B, NAD (soluble)	−1.33129
76713	1700039E15Rik	RIKEN cDNA 1700039E15 gene	0.931671
77128	Crebrf	CREB3 regulatory factor	0.658646
77252	9430038I01Rik	RIKEN cDNA 9430038I01 gene	0.636072
77521	Mtus2	microtubule associated tumor suppressor candidate 2	−0.75097
77645	Ptprtos	protein tyrosine phosphatase, receptor type T, opposite strand	1.675105
78082	9230117E06Rik	RIKEN cDNA 9230117E06 gene	−1.13148
78223	4930577H14Rik	RIKEN cDNA 4930577H14 gene	−0.89476
78257	Lrrc9	leucine rich repeat containing 9	0.847065
81489	Dnajb1	DnaJ (Hsp40) homolog, subfamily B, member 1	−0.67696
83491	Pramel1	preferentially expressed antigen in melanoma-like 1	−1.36516
83557	Lin28a	lin-28 homolog A (C. elegans)	0.674165
100061	Lrrc19	leucine rich repeat containing 19	0.722962
100855	Tbc1d14	TBC1 domain family, member 14	1.260915
102402	AA414992	expressed sequence AA414992	1.11508
102787	AW552889	expressed sequence AW552889	−0.78721
103161	Apof	apolipoprotein F	2.630612
104896	AI852580	expressed sequence AI852580	−0.76398
105271	AU017674	expressed sequence AU017674	−0.6189
108803	4933402P03Rik	RIKEN cDNA 4933402P03 gene	−0.94556
110902	Chrna2	cholinergic receptor, nicotinic, alpha polypeptide 2 (neuronal)	0.618069
113849	Vmn1r52	vomeronasal 1 receptor 52	−1.06252
114652	Ly6g5c	lymphocyte antigen 6 complex, locus G5C	−0.93519
117005	Olfr74	olfactory receptor 74	−1.04523
170484	Nphs2	nephrosis 2, podocin	−0.99487
171190	Vmn1r26	vomeronasal 1 receptor 26	−1.05033
171230	Vmn1r231	vomeronasal 1 receptor 231	−1.11209
171255	Vmn1r201	vomeronasal 1 receptor 201	−1.43574
171257	Vmn1r195	vomeronasal 1 receptor 195	−0.82871
171273	Vmn1r217	vomeronasal 1 receptor 217	−0.66816
171279	Vmn1r216	vomeronasal 1 receptor 216	−0.83986
171469	Gpr37l1	G protein-coupled receptor 37-like 1	−1.00965
193740	Hspa1a	heat shock protein 1A	−1.78004
195555	Pramef6	PRAME family member 6	1.168758
195564	Skint3	selection and upkeep of intraepithelial T cells 3	−0.91023
210373	A530095I07Rik	RIKEN cDNA A530095I07 gene	−0.90452
211383	Amer3	APC membrane recruitment 3	−1.01366
211770	Trib1	tribbles homolog 1 (Drosophila)	−0.81808
213332	AA474331	expressed sequence AA474331	−0.96037
214685	Chadl	chondroadherin-like	−0.61683
214899	Kdm5a	lysine (K)-specific demethylase 5A	0.640465
216766	Gemin5	gem (nuclear organelle) associated protein 5	1.158108
217695	Zfyve1	zinc finger, FYVE domain containing 1	0.646138
218341	Rfesd	Rieske (Fe-S) domain containing	0.761426
218772	Rarb	retinoic acid receptor, beta	−0.63463
223262	Timm8a2	translocase of inner mitochondrial membrane 8A2	0.763222
224754	H2-M11	histocompatibility 2, M region locus 11	1.200937
225579	Slc27a6	solute carrier family 27 (fatty acid transporter), member 6	−1.53388
226356	Cfap221	cilia and flagella associated protein 221	−0.60972
228094	Cerkl	ceramide kinase-like	1.033832
229615	Pias3	protein inhibitor of activated STAT 3	0.627799
231070	Insig1	insulin induced gene 1	0.823299
231503	Tmem150c	transmembrane protein 150C	1.284752
233210	Prr12	proline rich 12	0.604208
233230	Mrgprb4	MAS-related GPR, member B4	−0.99996
233544	A630091E08Rik	RIKEN cDNA A630091E08 gene	0.895014
235493	Fam214a	family with sequence similarity 214, member A	0.747692
237716	Gpr75	G protein-coupled receptor 75	0.60654
237831	Slc13a5	solute carrier family 13 (sodium-dependent citrate transporter), member 5	−0.75315
239167	Synb	syncytin b	−1.08261
239368	Erich5	glutamate rich 5	1.148509
240726	Slco5a1	solute carrier organic anion transporter family, member 5A1	−1.07061
241589	D430041D05Rik	RIKEN cDNA D430041D05 gene	−1.10155
242100	Pglyrp3	peptidoglycan recognition protein 3	−1.08284
244911	C2cd4a	C2 calcium-dependent domain containing 4A	−0.9676
252864	Dusp15	dual specificity phosphatase-like 15	−0.78664
252868	Odf4	outer dense fiber of sperm tails 4	−0.79222
257888	Olfr1386	olfactory receptor 1386	−1.78702
257935	Olfr1287	olfactory receptor 1287	0.742409
258046	Olfr951	olfactory receptor 951	−1.127
258069	Olfr787	olfactory receptor 787	−0.88202
258224	Olfr1358	olfactory receptor 1358	−0.84454
258236	Olfr391-ps	olfactory receptor 391, pseudogene	−0.70202
258285	Olfr122	olfactory receptor 122	−1.24794
258290	Olfr1143	olfactory receptor 1143	−0.7162
258366	Olfr434	olfactory receptor 434	−0.7381
258375	Olfr794	olfactory receptor 794	−0.85019
258507	Olfr96	olfactory receptor 96	−0.87258
258589	Olfr703	olfactory receptor 703	−0.66525
258590	Olfr702	olfactory receptor 702	−1.59164
258677	Olfr76	olfactory receptor 76	−0.91511
258716	Olfr424	olfactory receptor 424	−1.59652
258750	Olfr551	olfactory receptor 551	−0.60955
258825	Olfr975	olfactory receptor 975	−0.91639
258877	Olfr1395	olfactory receptor 1395	−0.93299
258926	Olfr476	olfactory receptor 476	−1.66123
259006	Olfr399	olfactory receptor 399	−1.18
259021	Olfr1054	olfactory receptor 1054	−0.93971
268390	Ahsa2	AHA1, activator of heat shock protein ATPase 2	−0.60857
268782	Agxt2	alanine-glyoxylate aminotransferase 2	0.692485
269023	Zfp608	zinc finger protein 608	0.679619
269604	Gpr157	G protein-coupled receptor 157	0.910932
269630	5031425E22Rik	RIKEN cDNA 5031425E22 gene	0.615826
270185	BC043934	cDNA sequence BC043934	0.589127
271697	Cdk15	cyclin-dependent kinase 15	−0.72819
271813	Agbl2	ATP/GTP binding protein-like 2	1.809137
272589	Tbcel	tubulin folding cofactor E-like	0.651273
277496	Lkaaear1	LKAAEAR motif containing 1 (IKAAEAR murine motif)	−1.31271
279499	Kctd19	potassium channel tetramerisation domain containing 19	1.183617
286940	Flnb	filamin, beta	−0.81259
319433	Serpine3	serpin peptidase inhibitor, clade E (nexin, plasminogen activator inhibitor type 1), member 3	−1.00758
319525	D330013E07Rik	RIKEN cDNA D330013E07 gene	0.69013
319587	4930539J05Rik	RIKEN cDNA 4930539J05 gene	0.604507
319615	Zfp944	zinc finger protein 944	0.631042
319805	C130073E24Rik	RIKEN cDNA C130073E24 gene	−1.43705
319839	A530020G20Rik	RIKEN cDNA A530020G20 gene	0.934835
319990	C730014E05Rik	RIKEN cDNA C730014E05 gene	−1.00032
320099	BC106179	cDNA sequence BC106179	0.833723
320178	4921529L05Rik	RIKEN cDNA 4921529L05 gene	0.934492
320323	A930038B10Rik	RIKEN cDNA A930038B10 gene	2.479141
320333	D830030K20Rik	RIKEN cDNA D830030K20 gene	0.806415
320771	C030040A22Rik	RIKEN cDNA C030040A22 gene	1.11646
320790	Chd7	chromodomain helicase DNA binding protein 7	0.595491
320940	Atp11c	ATPase, class VI, type 11C	0.606641
327780	4933439G19Rik	RIKEN cDNA 4933439G19 gene	−0.81745
328231	Gm5082	predicted gene 5082	1.265112
328354	Gm5087	predicted gene 5087	−0.95851
329509	1810024B03Rik	RIKEN cDNA 1810024B03 gene	0.86008
329679	Fnip2	folliculin interacting protein 2	0.684577
330031	Gm5106	predicted gene 5106	−1.01833
330149	Hfm1	HFM1, ATP-dependent DNA helicase homolog (S. cerevisiae)	−1.1337
330183	Gm16063	predicted gene 16063	−0.7011
331532	Tceal5	transcription elongation factor A (SII)-like 5	−0.77416
353371	Oxct2b	3-oxoacid CoA transferase 2B	1.40638
381350	Spag6l	sperm associated antigen 6 like	−2.56876
381352	Mamdc4	MAM domain containing 4	−0.81521
381544	Gm1661	predicted gene 1661	−0.58549
382053	Ces3a	carboxylesterase 3A	2.495337
382202	LOC382202	uncharacterized LOC382202	−1.22603
382253	Cdkl5	cyclin-dependent kinase-like 5	−1.18451
382551	Cd300lh	CD300 antigen like family member H	−0.8993
383243	Olfr128	olfactory receptor 128	−0.81569
384185	Arl9	ADP-ribosylation factor-like 9	−0.79354
385109	Igkv4-72	immunoglobulin kappa chain variable 4-72	0.782378
385343	Rhox1	reproductive homeobox 1	−0.74473
387285	Hcrtr2	hypocretin (orexin) receptor 2	−1.70246
403345	BC037438	cDNA sequence BC037438	−0.61835
404284	Vmn1r59	vomeronasal 1 receptor 59	−1.04937
404580	AL117821	expressed sequence AL117821	0.788304
408065	Zfp456	zinc finger protein 456	0.689588
414066	BC037032	cDNA Sequence BC037032	0.679343
414075	BC049265	cDNA sequence BC049265	−1.08853
414128	G630064G18Rik	RIKEN cDNA G630064G18 gene	0.678383
432842	LOC432842	uncharacterized LOC432842	−1.04016
433873	Gm9903	predicted gene 9903	0.774662
434228	Gm5600	predicted gene 5600	−0.80092
434282	Gm5608	predicted gene 5608	0.680106
434797	Gm5640	predicted gene 5640	−0.77576
435766	Tnni3k	TNNI3 interacting kinase	−0.98123
442825	A230083G16Rik	RIKEN cDNA A230083G16 gene	−0.83851
474160	Platr17	pluripotency associated transcript 17	−0.72374
504186	Chrna10	cholinergic receptor, nicotinic, alpha polypeptide 10	−2.03423
544763	Hbq1b	hemoglobin, theta 1B	−0.97634
544808	AA623943	expressed sequence AA623943	−1.17616
544881	BB287469	expressed sequence BB287469	−0.75438
546096	Gm29682	predicted gene, 29682	−0.95289
574519	Vax2os	ventral anterior homeobox 2, opposite strand	−0.752
622629	Gm10318	predicted gene 10318	0.763521
622733	Olfr764-ps1	olfactory receptor 764, pseudogene 1	−1.0235
626275	A930012L18Rik	RIKEN cDNA A930012L18 gene	0.800218
626299	Vmn1r194	vomeronasal 1 receptor 194	−1.05125
628185	Vmn2r112	vomeronasal 2, receptor 112	−1.75223
628422	Vmn2r58	vomeronasal 2, receptor 58	−0.95768
632534	Vmn1r191	vomeronasal 1 receptor 191	−1.16439
632687	March10	membrane-associated ring finger (C3HC4) 10	−0.69425
639390	BC094435	cDNA sequence BC094435	0.855954
653030	Arhgap27os3	Rho GTPase activating protein 27, opposite strand 3	−0.72239
665413	Gm7628	predicted gene 7628	0.705552
665466	Gm7644	predicted gene 7644	0.873983
665797	Gm7788	glyceraldehyde-3-phosphate dehydrogenase pseudogene	−1.09479
666919	Gm8363	non-SMC condensin II complex, subunit H2 pseudogene	1.216668
668208	Gm13288	predicted gene 13288	0.586814
723792	Pinc	pregnancy induced noncoding RNA	−0.80514
791273	B630006N21Rik	RIKEN cDNA B630006N21 gene	0.896019
791292	Gm9936	predicted gene 9936	−0.92668
791338	Gm10190	predicted gene 10190	−0.63181
791359	Gm9961	predicted gene 9961	−0.77249
791394	Gm9878	predicted gene 9878	1.279629
791415	Gm12500	predicted gene 12500	0.63538
100036523	Gm16982	predicted gene, 16982	1.878863
100036541	Gm9744	predicted gene 9744	0.840898
100038512	Gm12542	predicted gene 12542	−0.85287
100038580	6820445E23Rik	RIKEN cDNA 6820445E23 gene	0.704537
100038608	Gm10389	predicted gene 10389	−1.62243
100038721	Gm10781	predicted gene 10781	−0.66451
100038731	Gm10753	predicted gene 10753	−0.93414
100038736	Gm10209	predicted gene 10209	0.818848
100038752	Gm10825	predicted gene 10825	−0.9288
100039095	Gm2044	predicted gene 2044	−1.00928
100039286	Gm2138	predicted gene 2138	0.696008
100039344	Gm2172	predicted gene 2172	−0.77306
100039899	Gm2483	predicted gene 2483	−1.43561
100039946	Gm2511	predicted gene 2511	−0.8232
100040567	Gm12409	predicted gene 12409	−0.59888
100040972	Tceal7	transcription elongation factor A (SII)-like 7	−1.177
100041230	Hist1h4m	histone cluster 1, H4m	−0.84498
100041562	Gm14762	predicted gene 14762	0.804936
100041605	Gm3428	predicted gene 3428	−0.60764
100042202	Gm3718	predicted gene 3718	0.702933
100042679	Gm16386	zinc finger protein 946 pseudogene	0.667103
100042761	Gm10697	predicted gene 10697	0.726064
100042960	Gm4131	predicted gene 4131	−1.06387
100043063	Gm4203	predicted gene 4203	1.102296
100043123	Gm11710	predicted gene 11710	−0.73285
100043314	Tigit	T cell immunoreceptor with Ig and ITIM domains	0.996306
100043407	Gm4419	predicted gene 4419	0.650398
100043450	AA387883	expressed sequence AA387883	−1.05861
100043604	Vmn1r132	vomeronasal 1 receptor 132	2.563148
100043831	Gm4681	predicted gene 4681	−0.95104
100049155	D930027P08Rik	RIKEN cDNA D930027P08 gene	0.673712
100126224	Gm11747	predicted gene 11747	0.777315
100216455	Gm14124	predicted gene 14124	0.631449
100359411	Gm9798	predicted gene 9798	0.772542
100379611	Gm11767	predicted gene 11767	1.126146
100502644	AA419673	expressed sequence AA419673	−0.88892
100502868	Gm20597	predicted gene, 20597	0.782722
100502923	Gm17619	predicted gene, 17619	0.793983
100502967	Speer4c	spermatogenesis associated glutamate (E)-rich protein 4c	0.674691
100503007	Gm19500	predicted gene, 19500	−1.23075
100503292	Gm12279	predicted gene 12279	1.207112
100503307	Gm15408	predicted gene 15408	−1.05318
100503347	Gm19648	predicted gene, 19648	0.592679
100503474	Platr22	pluripotency associated transcript 22	−1.87808
100503481	4930453H23Rik	RIKEN cDNA 4930453H23 gene	−0.89361
100503498	Gm16675	predicted gene, 16675	0.74548
100503584	Zfp534	zinc finger protein 534	0.686315
100503790	Gm19897	predicted gene, 19897	−0.94176
100504007	Gm15787	predicted gene 15787	0.88342
100504207	Arhgap33os	Rho GTPase activating protein 33, opposite strand	1.28465
100504231	Gm15708	predicted gene 15708	0.705311
100504464	E230016K23Rik	RIKEN cDNA E230016K23 gene	0.666816
100504734	Gm16794	predicted gene, 16794	0.718494
101055939	LOC101055939	kinesin-like protein KIF19	−0.73348
101055983	Gm14569	predicted gene 14569	−0.85428
101056219	LOC101056219	uncharacterized LOC101056219	0.693525
102632036	Gm30211	predicted gene, 30211	−1.35883
102632061	Gm30234	predicted gene, 30234	−0.92374
102632158	Gm30305	predicted gene, 30305	−0.87698
102632434	Gm30509	predicted gene, 30509	−0.59183
102632567	Gm30606	predicted gene, 30606	−1.45024
102633273	Gm31137	predicted gene, 31137	−0.92847
102633545	Gm31344	predicted gene, 31344	−0.68016
102633682	Gm31447	predicted gene, 31447	−0.81639
102633740	Gm31493	predicted gene, 31493	−0.99917
102633780	Gm28321	predicted gene 28321	0.833415
102633974	Gm28905	predicted gene 28905	−1.02829
102634240	Gm31872	predicted gene, 31872	0.739554
102634384	Gm31980	predicted gene, 31980	−0.60487
102634445	Gm32026	predicted gene, 32026	0.893706
102634642	Gm32178	predicted gene, 32178	0.716286
102634759	Gm32265	predicted gene, 32265	−0.76659
102634913	Gm16070	predicted gene 16070	−0.73195
102634922	Gm32391	predicted gene, 32391	0.617974
102634964	Gm24474	predicted gene, 24474	−0.95048
102635707	LOC102635707	uncharacterized LOC102635707	−1.14721
102636049	Gm33228	predicted gene, 33228	−1.45513
102636446	Gm33509	predicted gene, 33509	1.151381
102636463	Gm38488	predicted gene, 38488	0.790117
102636681	Gm11342	predicted gene 11342	1.225075
102636777	LOC102636777	uncharacterized LOC102636777	−1.35573
102637182	Gm34059	predicted gene, 34059	−1.15365
102637349	Gm34184	predicted gene, 34184	−0.65116
102637460	Gm34263	predicted gene, 34263	−1.01607
102637485	Gm34280	predicted gene, 34280	−0.80203
102637601	Gm34368	predicted gene, 34368	1.341957
102637900	Gm34596	predicted gene, 34596	0.726827
102638146	Gm34780	predicted gene, 34780	−0.69991
102638234	Gm34843	predicted gene, 34843	0.712561
102638424	Gm34995	predicted gene, 34995	−0.6454
102638626	Gm35147	predicted gene, 35147	0.631503
102638746	Gm35236	predicted gene, 35236	−2.02371
102640039	Gm36208	predicted gene, 36208	−0.66706
102640419	Gm16322	predicted gene 16322	0.770732
102640765	Gm36757	predicted gene, 36757	0.746976
102640779	LOC102640779	uncharacterized LOC102640779	0.871822
102641312	LOC102641312	cytochrome P450 3A41-like	−1.07723
102641681	LOC102641681	uncharacterized LOC102641681	−1.06471
102641860	Gm26578	predicted gene, 26578	−0.64534
102641978	LOC102641978	uncharacterized LOC102641978	0.598838
102642028	LOC102642028	uncharacterized LOC102642028	−1.1585
102642104	LOC102642104	uncharacterized LOC102642104	−0.65471
102642578	LOC102642578	uncharacterized LOC102642578	0.962956
102642723	LOC102642723	uncharacterized LOC102642723	−0.59406
105242927	Gm38999	predicted gene, 38999	−0.60384
105243155	Gm26795	predicted gene, 26795	−0.91603
105245323	Gm40798	predicted gene, 40798	0.663404
105246049	Gm41408	predicted gene, 41408	0.8216
105246057	LOC105246057	igE-binding protein-like	0.672734
105246506	LOC105246506	uncharacterized LOC105246506	−0.70599
105247651	LOC105247651	uncharacterized LOC105247651	0.630204

**Table 2:** Differentially expressed genes (DEG) in the colon of mice after 7 days of exposure to E171.

**Entrez gene ID**	**Gene symbol**	**Gene name**	**Log2FC**
11304	Abca4	ATP-binding cassette, sub-family A (ABC1), member 4	−0.66413
11428	Aco1	aconitase 1	0.725006
11496	Adam22	a disintegrin and metallopeptidase domain 22	−0.69335
11498	Adam4	a disintegrin and metallopeptidase domain 4	0.810901
11549	Adra1a	adrenergic receptor, alpha 1a	−0.95816
11648	Akp3	alkaline phosphatase 3, intestine, not Mn requiring	−1.3038
11652	Akt2	thymoma viral proto-oncogene 2	0.802731
11684	Alox12	arachidonate 12-lipoxygenase	−1.24333
11686	Alox12b	arachidonate 12-lipoxygenase, 12R type	−2.15796
11688	Alox8	arachidonate 8-lipoxygenase	−2.48602
11765	Ap1g1	adaptor protein complex AP-1, gamma 1 subunit	0.803441
11789	Apc	adenomatosis polyposis coli	0.608252
11989	Slc7a3	solute carrier family 7 (cationic amino acid transporter, y+ system), member 3	−1.63447
12012	Baat	bile acid-Coenzyme A: amino acid N-acyltransferase	0.757634
12023	Barx2	BarH-like homeobox 2	−1.39414
12035	Bcat1	branched chain aminotransferase 1, cytosolic	−1.27812
12154	Bmp10	bone morphogenetic protein 10	−1.15523
12168	Bmpr2	bone morphogenetic protein receptor, type II (serine/threonine kinase)	0.808147
12180	Smyd1	SET and MYND domain containing 1	−0.86328
12296	Cacnb2	calcium channel, voltage-dependent, beta 2 subunit	1.086221
12497	Entpd6	ectonucleoside triphosphate diphosphohydrolase 6	0.642885
12608	Cebpb	CCAAT/enhancer binding protein (C/EBP), beta	−0.79497
12631	Cfl1	cofilin 1, non-muscle	0.690806
12660	Chka	choline kinase alpha	0.672888
12704	Cit	citron	0.858472
12750	Clk4	CDC like kinase 4	−0.70824
12776	Ccr8	chemokine (C-C motif) receptor 8	−0.9491
12836	Col7a1	collagen, type VII, alpha 1	−1.37157
12847	Copa	coatomer protein complex subunit alpha	0.659294
12922	Crhr2	corticotropin releasing hormone receptor 2	−0.83292
12931	Crlf1	cytokine receptor-like factor 1	−2.01808
12965	Crygb	crystallin, gamma B	−1.18537
12966	Crygc	crystallin, gamma C	0.60513
12992	Csn1s2b	casein alpha s2-like B	−1.13408
13162	Slc6a3	solute carrier family 6 (neurotransmitter transporter, dopamine), member 3	−1.15454
13168	Dbil5	diazepam binding inhibitor-like 5	−0.6261
13185	Dscr3	Down syndrome critical region gene 3	0.653566
13207	Ddx5	DEAD (Asp-Glu-Ala-Asp) box polypeptide 5	0.698294
13367	Diap1	diaphanous homolog 1 (Drosophila)	1.034071
13518	Dst	dystonin	0.841131
13522	Adam28	a disintegrin and metallopeptidase domain 28	−0.98253
13618	Ednrb	endothelin receptor type B	0.846174
13619	Phc1	polyhomeotic-like 1 (Drosophila)	−0.62828
13645	Egf	epidermal growth factor	−1.01864
13806	Eno1	enolase 1, alpha non-neuron	0.683879
13821	Epb4.1l1	erythrocyte protein band 4.1 like 1	0.592088
13822	Epb4.1l2	erythrocyte protein band 4.1 like 2	0.632162
13849	Ephx1	epoxide hydrolase 1, microsomal	−0.61294
13860	Eps8	epidermal growth factor receptor pathway substrate 8	0.78414
13982	Esr1	estrogen receptor 1 (alpha)	−0.60031
14013	Mecom	MDS1 and EVI1 complex locus	0.782283
14043	Ext2	exostoses (multiple) 2	0.625909
14050	Eya3	eyes absent 3 homolog (Drosophila)	0.591668
14264	Fmod	fibromodulin	−0.60553
14275	Folr1	folate receptor 1 (adult)	−0.60525
14343	Fut1	fucosyltransferase 1	−1.19602
14346	Fut4-ps1	fucosyltransferase 4, pseudogene 1	−0.70855
14367	Fzd5	frizzled homolog 5 (Drosophila)	0.653371
14459	Gast	gastrin	−1.77078
14463	Gata4	GATA binding protein 4	−1.75728
14552	Gdap7	ganglioside-induced differentiation-associated-protein 7	0.801168
14602	Ghrhr	growth hormone releasing hormone receptor	−0.99429
14619	Gjb2	gap junction protein, beta 2	−1.53492
14628	Ostm1	osteopetrosis associated transmembrane protein 1	−0.6966
14659	Glrp1	glutamine repeat protein 1	−0.73874
14664	Slc6a9	solute carrier family 6 (neurotransmitter transporter, glycine), member 9	−0.67572
14686	Gnat2	guanine nucleotide binding protein, alpha transducing 2	−0.96149
14788	Gpr162	G protein-coupled receptor 162	−0.90054
15260	Hira	histone cell cycle regulation defective homolog A (S. cerevisiae)	0.740336
15277	Hk2	hexokinase 2	−0.66671
15381	Hnrnpc	heterogeneous nuclear ribonucleoprotein C	0.672078
15401	Hoxa4	homeobox A4	0.824422
15423	Hoxc4	homeobox C4	−2.12955
15441	Hp1bp3	heterochromatin protein 1, binding protein 3	0.682279
15482	Hspa1l	heat shock protein 1-like	−1.394
15495	Hsd3b4	hydroxy-delta-5-steroid dehydrogenase, 3 beta- and steroid delta-isomerase 4	−1.67818
15932	Idua	iduronidase, alpha-L-	0.597911
16162	Il12rb2	interleukin 12 receptor, beta 2	−1.01715
16178	Il1r2	interleukin 1 receptor, type II	−1.93541
16187	Il3	interleukin 3	−2.15191
16333	Ins1	insulin I	−2.1446
16337	Insr	insulin receptor	0.602833
16402	Itga5	integrin alpha 5 (fibronectin receptor alpha)	1.552823
16415	Itgb2l	integrin beta 2-like	−1.39667
16468	Jarid2	jumonji, AT rich interactive domain 2	0.87411
16554	Kif13b	kinesin family member 13B	0.872857
16640	Klra9	killer cell lectin-like receptor subfamily A, member 9	−0.72199
16641	Klrc1	killer cell lectin-like receptor subfamily C, member 1	−1.64803
16661	Krt10	keratin 10	−2.78614
16728	L1cam	L1 cell adhesion molecule	0.808527
16869	Lhx1	LIM homeobox protein 1	−1.33538
16874	Lhx6	LIM homeobox protein 6	0.833857
16971	Lrp1	low density lipoprotein receptor-related protein 1	−0.65473
16979	Lrrn1	leucine rich repeat protein 1, neuronal	−2.85808
17002	Ltf	lactotransferrin	−3.67152
17184	Matr3	matrin 3	0.613994
17188	Maz	MYC-associated zinc finger protein (purine-binding transcription factor)	0.716238
17240	Mdfi	MyoD family inhibitor	−1.47494
17300	Foxc1	forkhead box C1	−1.72822
17311	Kitl	kit ligand	0.738123
17391	Mmp24	matrix metallopeptidase 24	1.012113
17863	Myb	myeloblastosis oncogene	0.958352
18145	Npc1	Niemann-Pick type C1	0.671266
18292	Sebox	SEBOX homeobox	−1.03382
18314	Olfr17	olfactory receptor 17	−0.91665
18315	Olfr18	olfactory receptor 18	−1.44533
18359	Olfr59	olfactory receptor 59	−2.08316
18365	Olfr65	olfactory receptor 65	−1.93793
18439	P2rx7	purinergic receptor P2X, ligand-gated ion channel, 7	−1.05118
18554	Pcsk7	proprotein convertase subtilisin/kexin type 7	0.596425
18571	Pdcd6ip	programmed cell death 6 interacting protein	0.688597
18576	Pde3b	phosphodiesterase 3B, cGMP-inhibited	0.699416
18599	Padi1	peptidyl arginine deiminase, type I	−1.40412
18600	Padi2	peptidyl arginine deiminase, type II	−0.92956
18626	Per1	period circadian clock 1	−0.618
18708	Pik3r1	phosphatidylinositol 3-kinase, regulatory subunit, polypeptide 1 (p85 alpha)	0.711339
18753	Prkcd	protein kinase C, delta	0.684102
18767	Pkia	protein kinase inhibitor, alpha	−0.80903
19152	Prtn3	proteinase 3	−1.40275
19206	Ptch1	patched homolog 1	1.232834
19208	Ptcra	pre T cell antigen receptor alpha	−0.96217
19229	Ptk2b	PTK2 protein tyrosine kinase 2 beta	0.605902
19337	Rab33a	RAB33A, member RAS oncogene family	−0.95374
19344	Rab5b	RAB5B, member RAS oncogene family	0.657767
19358	Rad23a	RAD23a homolog (S. cerevisiae)	0.585802
19377	Rai1	retinoic acid induced 1	0.757726
19400	Rapsn	receptor-associated protein of the synapse	−1.07501
19649	Robo3	roundabout homolog 3 (Drosophila)	−0.87983
19654	Rbm6	RNA binding motif protein 6	0.778903
19672	Rcn1	reticulocalbin 1	−0.611
19724	Rfx1	regulatory factor X, 1 (influences HLA class II expression)	0.763957
19730	Ralgds	ral guanine nucleotide dissociation stimulator	0.760822
20354	Sema4d	sema domain, immunoglobulin domain (Ig), transmembrane domain (TM) and short cytoplasmic domain, (semaphorin) 4D	−0.86875
20430	Cyfip1	cytoplasmic FMR1 interacting protein 1	0.717621
20471	Six1	sine oculis-related homeobox 1	−2.06588
20474	Six4	sine oculis-related homeobox 4	−2.28741
20482	Skil	SKI-like	0.743497
20538	Slc6a2	solute carrier family 6 (neurotransmitter transporter, noradrenalin), member 2	−1.95069
20586	Smarca4	SWI/SNF related, matrix associated, actin dependent regulator of chromatin, subfamily a, member 4	0.823711
20616	Snap91	synaptosomal-associated protein 91	−1.00674
20652	Soat1	sterol O-acyltransferase 1	0.763694
20661	Sort1	sortilin 1	0.744495
20680	Sox7	SRY (sex determining region Y)-box 7	−1.5204
20682	Sox9	SRY (sex determining region Y)-box 9	0.92932
20750	Spp1	secreted phosphoprotein 1	−2.47243
20779	Src	Rous sarcoma oncogene	0.618214
20856	Stc2	stanniocalcin 2	−1.06827
20913	Stxbp4	syntaxin binding protein 4	0.717037
20917	Suclg2	succinate-Coenzyme A ligase, GDP-forming, beta subunit	0.991799
20962	Sycp3	synaptonemal complex protein 3	1.533723
21337	Tacr2	tachykinin receptor 2	0.860064
21372	Tbl1x	transducin (beta)-like 1 X-linked	0.594507
21784	Tff1	trefoil factor 1	−0.86501
21816	Tgm1	transglutaminase 1, K polypeptide	−2.39971
21946	Pglyrp1	peptidoglycan recognition protein 1	0.676286
21951	Tnks	tankyrase, TRF1-interacting ankyrin-related ADP-ribose polymerase	0.624257
21987	Tpd52l1	tumor protein D52-like 1	−0.78499
22042	Tfrc	transferrin receptor	0.631074
22059	Trp53	transformation related protein 53	0.607334
22239	Ugt8a	UDP galactosyltransferase 8A	−1.8794
22287	Scgb1a1	secretoglobin, family 1A, member 1 (uteroglobin)	−1.7998
22296	Vmn1r51	vomeronasal 1 receptor 51	1.806044
22378	Wbp2	WW domain binding protein 2	0.673888
22410	Wnt10b	wingless-type MMTV integration site family, member 10B	−0.77983
22422	Wnt7b	wingless-type MMTV integration site family, member 7B	−1.2964
22427	Wrn	Werner syndrome homolog (human)	0.689264
22688	Zfp26	zinc finger protein 26	0.702018
22750	Zfp9	zinc finger protein 9	−0.74203
22775	Zik1	zinc finger protein interacting with K protein 1	−0.83276
23789	Coro1b	coronin, actin binding protein 1B	0.643091
23790	Coro1c	coronin, actin binding protein 1C	0.630664
23985	Slc26a4	solute carrier family 26, member 4	−1.05828
24044	Scamp2	secretory carrier membrane protein 2	0.628678
24102	Trex2	three prime repair exonuclease 2	−3.56133
26570	Slc7a11	solute carrier family 7 (cationic amino acid transporter, y+ system), member 11	−1.64966
26920	Cntrl	centriolin	0.638412
26936	Mprip	myosin phosphatase Rho interacting protein	0.829495
26942	Spag1	sperm associated antigen 1	−0.95497
26965	Cul1	cullin 1	0.587056
27382	Tcl1b5	T cell leukemia/lymphoma 1B, 5	−0.67158
27411	Slc14a2	solute carrier family 14 (urea transporter), member 2	−0.61673
27493	A230006K03Rik	RIKEN cDNA A230006K03 gene	0.713618
28006	Fam21	family with sequence similarity 21	0.700624
28114	Nsun2	NOL1/NOP2/Sun domain family member 2	0.748181
28248	Slco1a1	solute carrier organic anion transporter family, member 1a1	−0.83729
29848	Olfr29-ps1	olfactory receptor 29, pseudogene 1	−0.88445
30060	Mfi2	antigen p97 (melanoma associated) identified by monoclonal antibodies 133.2 and 96.5	−0.97835
50772	Mapk6	mitogen-activated protein kinase 6	0.700356
50781	Dkk3	dickkopf homolog 3 (Xenopus laevis)	0.674366
50934	Slc7a8	solute carrier family 7 (cationic amino acid transporter, y+ system), member 8	−1.28248
50994	Mtag2	metastasis associated gene 2	−1.28582
52108	D9Ertd115e	DNA segment, Chr 9, ERATO Doi 115, expressed	−0.69497
52850	Sgsm1	small G protein signaling modulator 1	0.768399
53322	Nucb2	nucleobindin 2	−1.29668
53381	Prdx4	peroxiredoxin 4	−0.59671
54126	Arhgef7	Rho guanine nucleotide exchange factor (GEF7)	0.671002
54199	Ccrl2	chemokine (C-C motif) receptor-like 2	−0.80972
54338	Slc23a2	solute carrier family 23 (nucleobase transporters), member 2	0.678156
54383	Phc2	polyhomeotic-like 2 (Drosophila)	0.702052
54448	Il1f6	interleukin 1 family, member 6	−3.48841
54611	Pde3a	phosphodiesterase 3A, cGMP inhibited	0.612094
54652	Cacna1f	calcium channel, voltage-dependent, alpha 1F subunit	0.914421
55981	Pigb	phosphatidylinositol glycan anchor biosynthesis, class B	0.74657
55993	Msh4	mutS homolog 4 (E. coli)	1.158784
56070	Tcerg1	transcription elongation regulator 1 (CA150)	−0.74169
56149	Grasp	GRP1 (general receptor for phosphoinositides 1)-associated scaffold protein	−0.87899
56215	Acin1	apoptotic chromatin condensation inducer 1	0.594454
56219	Extl1	exostoses (multiple)-like 1	−1.27118
56306	Fam60a	family with sequence similarity 60, member A	0.636922
56384	Letm1	leucine zipper-EF-hand containing transmembrane protein 1	0.784147
56406	Ncoa6	nuclear receptor coactivator 6	0.637659
56468	Socs5	suppressor of cytokine signaling 5	0.843892
56506	Cib2	calcium and integrin binding family member 2	−0.60266
56538	Klk11	kallikrein related-peptidase 11	−2.3751
57277	Slurp1	secreted Ly6/Plaur domain containing 1	−2.95558
57738	Slc15a2	solute carrier family 15 (H+/peptide transporter), member 2	−1.30016
58170	Asic5	acid-sensing (proton-gated) ion channel family member 5	−1.42954
58214	Cst10	cystatin 10 (chondrocytes)	−1.19353
58865	Tdh	L-threonine dehydrogenase	−2.57185
59026	Huwe1	HECT, UBA and WWE domain containing 1	0.622268
59083	Fetub	fetuin beta	−2.29439
63913	Fam129a	family with sequence similarity 129, member A	0.61201
64176	Sv2b	synaptic vesicle glycoprotein 2 b	−0.92457
64340	Dhx38	DEAH (Asp-Glu-Ala-His) box polypeptide 38	0.645424
64654	Fgf23	fibroblast growth factor 23	0.61382
65086	Lpar3	lysophosphatidic acid receptor 3	−0.70333
65107	Lrp10	low-density lipoprotein receptor-related protein 10	0.593185
66011	Ranbp17	RAN binding protein 17	−0.91862
66084	Rmnd1	required for meiotic nuclear division 1 homolog (S. cerevisiae)	0.609075
66127	1110014L15Rik	RIKEN cDNA 1110014L15 gene	−0.95708
66240	Kcne1l	potassium voltage-gated channel, Isk-related family, member 1-like, pseudogene	−2.1844
66313	Smurf2	SMAD specific E3 ubiquitin protein ligase 2	0.696636
66341	Eid3	EP300 interacting inhibitor of differentiation 3	0.613088
66344	Lce3b	late cornified envelope 3B	−3.89494
66561	Teddm3	transmembrane epididymal family member 3	−3.46887
66637	Tsen15	tRNA splicing endonuclease 15 homolog (S. cerevisiae)	0.734184
66829	Lrrc75aos2	leucine rich repeat containing 75A, opposite strand 2	0.916303
66830	Nacc1	nucleus accumbens associated 1, BEN and BTB (POZ) domain containing	1.249668
66839	0610009O20Rik	RIKEN cDNA 0610009O20 gene	0.742334
66898	Baiap2l1	BAI1-associated protein 2-like 1	0.597833
66932	Rexo1	REX1, RNA exonuclease 1 homolog (S. cerevisiae)	0.61969
66939	Aagab	alpha- and gamma-adaptin binding protein	0.604241
66957	Serpinb11	serine (or cysteine) peptidase inhibitor, clade B (ovalbumin), member 11	−2.87384
67040	Ddx17	DEAD (Asp-Glu-Ala-Asp) box polypeptide 17	0.629016
67088	Cand2	cullin-associated and neddylation-dissociated 2 (putative)	−0.75137
67289	3110021A11Rik	RIKEN cDNA 3110021A11 gene	0.741633
67327	1700031L13Rik	RIKEN cDNA 1700031L13 gene	−1.50459
67342	Kcnmb4os1	potassium large conductance calcium-activated channel, subfamily M, beta member 4, opposite strand 1	−1.5008
67492	Zfand4	zinc finger, AN1-type domain 4	−0.91248
67498	Kcnv1	potassium channel, subfamily V, member 1	−1.04995
67547	Slc39a8	solute carrier family 39 (metal ion transporter), member 8	0.917607
67561	Wdr48	WD repeat domain 48	0.646133
67685	Dyx1c1	dyslexia susceptibility 1 candidate 1 homolog (human)	−0.63112
67712	Slc25a37	solute carrier family 25, member 37	0.683473
67848	Ddx55	DEAD (Asp-Glu-Ala-Asp) box polypeptide 55	0.723513
67928	Abca14	ATP-binding cassette, sub-family A (ABC1), member 14	−0.9217
67956	Setd8	SET domain containing (lysine methyltransferase) 8	0.754544
67978	Tctn2	tectonic family member 2	0.630605
67988	Tmx3	thioredoxin-related transmembrane protein 3	0.839764
68149	Otub2	OTU domain, ubiquitin aldehyde binding 2	−0.78583
68175	4930591A17Rik	RIKEN cDNA 4930591A17 gene	−0.77172
68178	Cgnl1	cingulin-like 1	0.679068
68214	Gsto2	glutathione S-transferase omega 2	−0.79791
68226	Efcab2	EF-hand calcium binding domain 2	0.687146
68228	1700095K22Rik	RIKEN cDNA 1700095K22 gene	−0.83216
68311	Lypd2	Ly6/Plaur domain containing 2	−1.51522
68396	Nat8	N-acetyltransferase 8 (GCN5-related, putative)	−1.49203
68465	Adipor2	adiponectin receptor 2	0.767648
68697	1110036E04Rik	RIKEN cDNA 1110036E04 gene	0.878523
68744	Zfp740	zinc finger protein 740	0.627958
68770	Phtf2	putative homeodomain transcription factor 2	−0.67831
68964	Ctc1	CTS telomere maintenance complex component 1	0.736298
69031	Galnt6os	UDP-N-acetyl-alpha-D-galactosamine:polypeptide N-acetylgalactosaminyltransferase 6, opposite strand	−1.56979
69065	Chac1	ChaC, cation transport regulator 1	−1.65248
69202	Ptms	parathymosin	0.610983
69206	2010016I18Rik	RIKEN cDNA 2010016I18 gene	0.805808
69207	Srsf11	serine/arginine-rich splicing factor 11	0.592404
69307	Pxt1	peroxisomal, testis specific 1	−0.86674
69309	Slc16a13	solute carrier family 16 (monocarboxylic acid transporters), member 13	0.614244
69349	1700008O03Rik	RIKEN cDNA 1700008O03 gene	−0.79128
69374	1700023A20Rik	RIKEN cDNA 1700023A20 gene	−1.51032
69384	Tmem89	transmembrane protein 89	1.234292
69432	1700026J14Rik	RIKEN cDNA 1700026J14 gene	−1.71345
69489	2310007J06Rik	RIKEN cDNA 2310007J06 gene	−1.02391
69511	Klk12	kallikrein related-peptidase 12	−2.12594
69543	Capns2	calpain, small subunit 2	−1.90891
69571	2310034O05Rik	RIKEN cDNA 2310034O05 gene	−0.63191
69602	Otop3	otopetrin 3	−2.23857
69739	2410004I01Rik	RIKEN cDNA 2410004I01 gene	−0.64746
69772	Bdh2	3-hydroxybutyrate dehydrogenase, type 2	−0.91556
69857	1810053B23Rik	RIKEN cDNA 1810053B23 gene	−2.51944
69926	Dnah17	dynein, axonemal, heavy chain 17	−1.12593
70007	1700029J08Rik	RIKEN cDNA 1700029J08 gene	−0.81527
70055	1700030L20Rik	RIKEN cDNA 1700030L20 gene	−1.80562
70061	Sdr9c7	4short chain dehydrogenase/reductase family 9C, member 7	−1.6906
70230	3300002P13Rik	RIKEN cDNA 3300002P13 gene	−0.9735
70237	Bhlhb9	basic helix-loop-helix domain containing, class B9	−0.61368
70296	Tbc1d13	TBC1 domain family, member 13	0.680468
70355	Gprc5c	G protein-coupled receptor, family C, group 5, member C	−0.65647
70439	Taf15	TAF15 RNA polymerase II, TATA box binding protein (TBP)-associated factor	0.658771
70470	Rprd1b	regulation of nuclear pre-mRNA domain containing 1B	0.644537
70568	Cpne3	copine III	0.771544
70730	6330409D20Rik	RIKEN cDNA 6330409D20 gene	−1.0955
70793	Gm9725	predicted gene 9725	0.849167
70817	4633402D09Rik	RIKEN cDNA 4633402D09 gene	−1.04343
70835	Prss22	protease, serine 22	−1.81815
70963	4931402H11Rik	RIKEN cDNA 4931402H11 gene	1.281475
71065	4933407O12Rik	RIKEN cDNA 4933407O12 gene	−0.73158
71067	4933411E08Rik	RIKEN cDNA 4933411E08 gene	−1.21629
71078	Adam30	a disintegrin and metallopeptidase domain 30	−1.12467
71128	4933417C20Rik	RIKEN cDNA 4933417C20 gene	0.878534
71137	Rfx4	regulatory factor X, 4 (influences HLA class II expression)	−1.01222
71263	Mro	maestro	−1.13673
71406	5430416O09Rik	RIKEN cDNA 5430416O09 gene	−0.88034
71650	4930456L15Rik	RIKEN cDNA 4930456L15 gene	−0.91403
71720	Osbpl3	oxysterol binding protein-like 3	−0.58902
71760	Etnppl	ethanolamine phosphate phospholyase	−0.98218
71774	Shroom1	shroom family member 1	−0.7375
71781	Slc16a14	solute carrier family 16 (monocarboxylic acid transporters), member 14	−1.92754
71861	Zswim2	zinc finger SWIM-type containing 2	−2.55208
71864	Fam217a	family with sequence similarity 217, member A	0.73753
71955	Ist1	increased sodium tolerance 1 homolog (yeast)	0.5985
71996	1600014K23Rik	RIKEN cDNA 1600014K23 gene	−1.15263
72014	Btbd17	BTB (POZ) domain containing 17	−0.65561
72061	2010111I01Rik	RIKEN cDNA 2010111I01 gene	0.825361
72145	Wdfy3	WD repeat and FYVE domain containing 3	0.725186
72147	Zbtb46	zinc finger and BTB domain containing 46	−0.59109
72185	Dbndd1	dysbindin (dystrobrevin binding protein 1) domain containing 1	−0.95824
72242	Psg21	pregnancy-specific glycoprotein 21	−1.09573
72252	1700022A21Rik	glycerol-3-phosphate dehydrogenase 1-like pseudogene	1.288523
72475	Ssbp3	single-stranded DNA binding protein 3	0.93094
72523	2700005E23Rik	RIKEN cDNA 2700005E23 gene	−0.64663
72667	Zfp444	zinc finger protein 444	0.70073
72739	Zkscan3	zinc finger with KRAB and SCAN domains 3	0.618544
72747	Ttc39c	tetratricopeptide repeat domain 39C	−0.69873
72753	2810442N19Rik	RIKEN cDNA 2810442N19 gene	−1.00662
72805	Zfp839	zinc finger protein 839	0.599802
72925	March1	membrane-associated ring finger (C3HC4) 1	0.850855
73009	2900057B20Rik	RIKEN cDNA 2900057B20 gene	−0.88695
73106	Prss57	protease, serine 57	−0.77097
73130	Tmed5	transmembrane emp24 protein transport domain containing 5	−0.67232
73191	Fezf1	Fez family zinc finger 1	−1.33411
73192	Xpot	exportin, tRNA (nuclear export receptor for tRNAs)	−0.66157
73395	1700049E22Rik	RIKEN cDNA 1700049E22 gene	−0.95191
73407	Tepp	testis, prostate and placenta expressed	−0.6637
73440	1700062C10Rik	RIKEN cDNA 1700062C10 gene	−1.10313
73466	Ms4a13	membrane-spanning 4-domains, subfamily A, member 13	−1.24695
73603	Trp53tg5	transformation related protein 53 target 5	−0.89139
73606	1700120E14Rik	RIKEN cDNA 1700120E14 gene	0.969043
73696	Platr9	pluripotency associated transcript 9	0.690679
73707	Gucy2g	guanylate cyclase 2g	−2.27127
73719	Lce1c	late cornified envelope 1C	−2.72812
73863	4930415O20Rik	RIKEN cDNA 4930415O20 gene	−0.81284
73941	4930412L05Rik	RIKEN cDNA 4930412L05 gene	−2.32861
73949	Speer9-ps1	spermatogenesis associated glutamate (E)-rich protein 9, pseudogene 1	−1.33639
73994	4930449C09Rik	RIKEN cDNA 4930449C09 gene	−0.72145
74007	Btbd11	BTB (POZ) domain containing 11	−0.79803
74076	4933406C10Rik	RIKEN cDNA 4933406C10 gene	−0.96732
74081	Cep350	centrosomal protein 350	0.849242
74127	Krt80	keratin 80	−1.91014
74222	sep-14	septin 14	−2.68921
74253	Klrg2	killer cell lectin-like receptor subfamily G, member 2	−1.52826
74286	Tbc1d21	TBC1 domain family, member 21	−1.17616
74296	1700093J21Rik	RIKEN cDNA 1700093J21 gene	−0.66263
74297	1700106J16Rik	RIKEN cDNA 1700106J16 gene	−1.67683
74302	Mtmr3	myotubularin related protein 3	0.636379
74351	Ddx23	DEAD (Asp-Glu-Ala-Asp) box polypeptide 23	0.586899
74369	mei-01	meiosis defective 1	−0.58684
74386	Rmi1	RMI1, RecQ mediated genome instability 1, homolog (S. cerevisiae)	0.712505
74467	Pus10	pseudouridylate synthase 10	−0.61067
74525	8430419L09Rik	RIKEN cDNA 8430419L09 gene	−0.78417
74634	4930423D22Rik	RIKEN cDNA 4930423D22 gene	−0.74361
74849	4930412F12Rik	RIKEN cDNA 4930412F12 gene	0.871862
74864	4930449E01Rik	RIKEN cDNA 4930449E01 gene	−0.759
75060	4930506C21Rik	RIKEN cDNA 4930506C21 gene	−0.84533
75097	Ube2dnl2	ubiquitin-conjugating enzyme E2D N-terminal like 2	−1.2845
75216	Cep128	centrosomal protein 128	0.655509
75280	4930554G24Rik	RIKEN cDNA 4930554G24 gene	1.010478
75309	4930565D16Rik	RIKEN cDNA 4930565D16 gene	−1.59875
75311	4930550C14Rik	RIKEN cDNA 4930550C14 gene	−1.83876
75400	Defb29	defensin beta 29	−1.45368
75465	Dynlrb2	dynein light chain roadblock-type 2	−1.06277
75497	Fabp12	fatty acid binding protein 12	−1.16257
75510	Izumo2	IZUMO family member 2	−1.36381
75535	1700016D02Rik	RIKEN cDNA 1700016D02 gene	−1.1259
75549	1700019L22Rik	RIKEN cDNA 1700019L22 gene	−1.04531
75571	Spata9	spermatogenesis associated 9	−0.59263
75689	Higd1b	HIG1 domain family, member 1B	−1.02087
75690	Vsig10l	ZV-set and immunoglobulin domain containing 10 like	−1.14018
75729	Fam227a	family with sequence similarity 227, member A	1.220646
75862	4930572O13Rik	RIKEN cDNA 4930572O13 gene	−0.98271
75863	Clec4g	C-type lectin domain family 4, member g	−1.13827
75880	4930592A05Rik	RIKEN cDNA 4930592A05 gene	−0.85837
75900	4930566D17Rik	RIKEN cDNA 4930566D17 gene	−0.94911
75947	4930594O21Rik	RIKEN cDNA 4930594O21 gene	−0.80557
76007	Zmym2	zinc finger, MYM-type 2	0.595213
76223	Agbl3	ATP/GTP binding protein-like 3	−1.06288
76382	1700012A03Rik	RIKEN cDNA 1700012A03 gene	−1.55564
76486	Ly6k	lymphocyte antigen 6 complex, locus K	−0.74361
76614	Immt	inner membrane protein, mitochondrial	0.772655
76640	1700113H08Rik	RIKEN cDNA 1700113H08 gene	−1.32126
76645	Pkd1l2	polycystic kidney disease 1 like 2	−0.94642
76936	Hnrnpm	heterogeneous nuclear ribonucleoprotein M	0.880421
76954	St5	suppression of tumorigenicity 5	0.802365
77015	Mpped2	metallophosphoesterase domain containing 2	−0.71754
77044	Arid2	AT rich interactive domain 2 (ARID, RFX-like)	−0.74807
77125	Il33	interleukin 33	−1.39979
77188	A430105D02Rik	RIKEN cDNA A430105D02 gene	0.683667
77207	8030425K09Rik	RIKEN cDNA 8030425K09 gene	−0.85832
77271	9430024F10Rik	RIKEN cDNA 9430024F10 gene	0.630722
77397	9530003J23Rik	RIKEN cDNA 9530003J23 gene	0.979814
77417	C030013C21Rik	RIKEN cDNA C030013C21 gene	0.644658
77552	Shisa4	shisa family member 4	−0.72836
77577	Spns3	spinster homolog 3	−1.06179
77592	4931406H21Rik	RIKEN cDNA 4931406H21 gene	0.720232
77703	9230106L01Rik	RIKEN cDNA 9230106L01 gene	−1.30358
77884	6720427H10Rik	RIKEN cDNA 6720427H10 gene	1.162931
77940	A930004D18Rik	RIKEN cDNA A930004D18 gene	−0.83788
78031	4930547E08Rik	RIKEN cDNA 4930547E08 gene	−1.20674
78154	4930430O22Rik	RIKEN cDNA 4930430O22 gene	−0.94561
78172	4930487D11Rik	RIKEN cDNA 4930487D11 gene	−2.39208
78192	4930540I17Rik	RIKEN cDNA 4930540I17 gene	−1.56933
78211	4930558N11Rik	RIKEN cDNA 4930558N11 gene	0.61235
78279	5330421C15Rik	RIKEN cDNA 5330421C15 gene	−0.93899
78500	1700063D05Rik	RIKEN cDNA 1700063D05 gene	−1.51247
78629	1700072I22Rik	RIKEN cDNA 1700072I22 gene	−0.59109
78757	Rictor	RPTOR independent companion of MTOR, complex 2	0.632871
78758	4921518K17Rik	RIKEN cDNA 4921518K17 gene	−0.9143
78762	4933424L21Rik	RIKEN cDNA 4933424L21 gene	−1.34361
78772	Hhipl2	hedgehog interacting protein-like 2	0.830186
78829	Tsc22d4	TSC22 domain family, member 4	0.608479
78926	Gas2l1	growth arrest-specific 2 like 1	0.821025
79362	Bhlhe41	basic helix-loop-helix family, member e41	−1.25631
79459	Aldoart2	aldolase 1 A, retrogene 2	1.047077
80885	Hcar2	hydroxycarboxylic acid receptor 2	−1.4179
80913	Pum2	pumilio RNA-binding family member 2	0.685887
80981	Arl4d	ADP-ribosylation factor-like 4D	−0.98255
81702	Ankrd17	ankyrin repeat domain 17	0.605263
81904	Cacng7	calcium channel, voltage-dependent, gamma subunit 7	−0.65114
83766	Actl6b	actin-like 6B	−1.09156
83796	Smarcd2	SWI/SNF related, matrix associated, actin dependent regulator of chromatin, subfamily d, member 2	0.759713
93897	Fzd10	frizzled homolog 10 (Drosophila)	−1.96894
94060	Lce3c	late cornified envelope 3C	−4.23
94184	Pdxdc1	pyridoxal-dependent decarboxylase domain containing 1	0.841541
94353	Hmgn3	high mobility group nucleosomal binding domain 3	−0.63599
97086	Slc9b2	solute carrier family 9, subfamily B (NHA2, cation proton antiporter 2), member 2	−0.8071
97122	Hist2h4	histone cluster 2, H4	0.763623
99094	AI849538	expressed sequence AI849538	0.6666
99167	Ssx2ip	synovial sarcoma, X breakpoint 2 interacting protein	0.645126
99296	Hrh3	histamine receptor H3	−0.84015
100088	Rcc1	regulator of chromosome condensation 1	0.643165
100470	Lao1	L-amino acid oxidase 1	−2.07078
101100	Ttll3	tubulin tyrosine ligase-like family, member 3	0.837423
101533	Klk9	kallikrein related-peptidase 9	−2.37858
102570	Slc22a13	solute carrier family 22 (organic cation transporter), member 13	−0.90109
102626	Mapkapk3	mitogen-activated protein kinase-activated protein kinase 3	0.621833
103067	AA522020	expressed sequence AA522020	0.655432
103080	sep-10	septin 10	0.681456
103268	Cep57l1	centrosomal protein 57-like 1	0.773083
103846	AI845619	expressed sequence AI845619	−0.92823
104248	Cabin1	calcineurin binding protein 1	0.613774
104362	Meig1	meiosis expressed gene 1	−1.59234
104681	Slc16a6	solute carrier family 16 (monocarboxylic acid transporters), member 6	−0.63591
104708	BB217526	expressed sequence BB217526	0.657657
104859	Tecpr2	tectonin beta-propeller repeat containing 2	0.593026
105005	Fam84a	family with sequence similarity 84, member A	−1.76563
105352	Dusp22	dual specificity phosphatase 22	−0.96787
105511	Fam170b	family with sequence similarity 170, member B	−1.73188
105653	Phyhip	phytanoyl-CoA hydroxylase interacting protein	−1.86543
106068	Slc45a4	solute carrier family 45, member 4	0.734658
106633	Ift140	intraflagellar transport 140	0.828963
106762	AW047481	expressed sequence AW047481	0.806171
107448	Unc5a	unc-5 homolog A (C. elegans)	−0.92021
107477	Guca1b	guanylate cyclase activator 1B	−0.72256
107503	Atf5	activating transcription factor 5	−0.64083
107515	Lgr4	leucine-rich repeat-containing G protein-coupled receptor 4	0.968492
108800	Ston2	stonin 2	0.747513
108832	Tmem74b	transmembrane protein 74B	−0.84324
108897	Aif1l	allograft inflammatory factor 1-like	−1.1509
109095	Rbm15b	RNA binding motif protein 15B	0.658633
109198	6030407O03Rik	RIKEN cDNA 6030407O03 gene	−1.2255
109342	Slc5a10	solute carrier family 5 (sodium/glucose cotransporter), member 10	−1.11164
109359	Fam175b	family with sequence similarity 175, member B	0.660947
109754	Cyb5r3	cytochrome b5 reductase 3	0.61693
109857	Cbr3	carbonyl reductase 3	−0.70278
109978	Art4	ADP-ribosyltransferase 4	−1.81093
110175	Ggct	gamma-glutamyl cyclotransferase	−0.68327
110637	Grik4	glutamate receptor, ionotropic, kainate 4	−0.72295
113845	Vmn1r48	vomeronasal 1 receptor 48	−1.049
113846	Vmn1r47	vomeronasal 1 receptor 47	−1.51436
113847	Vmn1r43	vomeronasal 1 receptor 43	−1.70758
114585	D17H6S53E	DNA segment, Chr 17, human D6S53E	0.70983
114615	Elac1	elaC homolog 1 (E. coli)	−0.71608
116811	Zim3	zinc finger, imprinted 3	−1.69901
116903	Calcb	calcitonin-related polypeptide, beta	−1.87798
116972	Fam57a	family with sequence similarity 57, member A	−0.95462
117147	Acsm1	acyl-CoA synthetase medium-chain family member 1	−1.20031
117590	Asb10	ankyrin repeat and SOCS box-containing 10	−1.11281
140546	Eri3	exoribonuclease 3	0.632673
140709	Col26a1	collagen, type XXVI, alpha 1	−1.00617
140904	Caln1	calneuron 1	−1.16418
170458	Gpha2	glycoprotein hormone alpha 2	−0.78142
170719	Oxr1	oxidation resistance 1	0.632419
170733	Klra17	killer cell lectin-like receptor, subfamily A, member 17	−1.34296
170735	Arr3	arrestin 3, retinal	−0.91118
170740	Zfp287	zinc finger protein 287	0.589692
170744	Tlr8	toll-like receptor 8	−0.82869
170829	Tram2	translocating chain-associating membrane protein 2	0.708449
171229	Vmn1r227	vomeronasal 1 receptor 227	−1.03346
171235	Vmn1r236	vomeronasal 1 receptor 236	−1.86013
171248	Vmn1r214	vomeronasal 1 receptor 214	−1.31339
171260	Vmn1r89	vomeronasal 1 receptor 89	−1.56777
171275	Vmn1r212	vomeronasal 1 receptor 212	−1.56988
171281	Acot3	acyl-CoA thioesterase 3	0.678602
171580	Mical1	microtubule associated monooxygenase, calponin and LIM domain containing 1	1.013549
192194	Btnl10	butyrophilin-like 10	0.811317
192285	Phf21a	PHD finger protein 21A	0.905948
192734	Lrrc75b	leucine rich repeat containing 75B	−1.02733
194655	Klf11	Kruppel-like factor 11	−0.89779
194908	Pld6	phospholipase D family, member 6	−0.68125
195564	Skint3	selection and upkeep of intraepithelial T cells 3	−1.27937
195733	Grhl1	grainyhead-like 1 (Drosophila)	−1.9831
207209	Ccdc154	coiled-coil domain containing 154	−1.03486
208643	Eif4g1	eukaryotic translation initiation factor 4, gamma 1	0.706288
208691	Eif5a2	eukaryotic translation initiation factor 5A2	−0.58893
208982	Hmgcll1	3-hydroxymethyl-3-methylglutaryl-Coenzyme A lyase-like 1	−0.83101
209824	Vmn1r183	vomeronasal 1 receptor 183	−2.24989
211134	Lzts1	leucine zipper, putative tumor suppressor 1	−1.10406
211550	Tifa	TRAF-interacting protein with forkhead-associated domain	−0.6945
212390	Klhl32	kelch-like 32	−0.69072
212528	Trmt1	tRNA methyltransferase 1	0.947646
212952	Gm44	predicted gene 44	−1.96356
213438	A630033H20Rik	RIKEN cDNA A630033H20 gene	−0.75217
213498	Arhgef11	Rho guanine nucleotide exchange factor (GEF) 11	0.639783
213696	Duoxa1	dual oxidase maturation factor 1	−2.2442
213783	Plekhg1	pleckstrin homology domain containing, family G (with RhoGef domain) member 1	−0.84573
213948	Atg9b	autophagy related 9B	−2.11574
214812	Zfp609	zinc finger protein 609	0.722991
215015	Fam20b	family with sequence similarity 20, member B	0.757405
215274	Il1f10	interleukin 1 family, member 10	−1.46053
215456	Gpat2	glycerol-3-phosphate acyltransferase 2, mitochondrial	−1.12537
215819	Nhsl1	NHS-like 1	0.816479
215999	Mcu	mitochondrial calcium uniporter	0.8517
216033	Ctnna3	catenin (cadherin associated protein), alpha 3	−2.53526
216188	Aldh1l2	aldehyde dehydrogenase 1 family, member L2	−1.71
216238	Eea1	early endosome antigen 1	0.67036
216377	Gm38403	predicted gene, 38403	0.858941
216578	Papolg	poly(A) polymerase gamma	0.639371
216643	Gabrp	gamma-aminobutyric acid (GABA) A receptor, pi	−1.21446
216835	Usp43	ubiquitin specific peptidase 43	0.940283
216892	Spns2	spinster homolog 2	−1.27362
217217	Asb16	ankyrin repeat and SOCS box-containing 16	−0.98193
217294	BC006965	cDNA sequence BC006965	−0.83067
217371	Rab40b	Rab40B, member RAS oncogene family	−0.85711
217674	Gphb5	glycoprotein hormone beta 5	−1.60542
217837	Itpk1	inositol 1,3,4-triphosphate 5/6 kinase	0.652385
217866	Cdc42bpb	CDC42 binding protein kinase beta	0.830481
218820	Zfp503	zinc finger protein 503	−0.76933
219150	Hmbox1	homeobox containing 1	0.948487
223658	Mroh1	maestro heat-like repeat family member 1	0.745331
223809	Smgc	submandibular gland protein C	−1.3385
224023	Klhl22	kelch-like 22	1.000464
224093	Fam43a	family with sequence similarity 43, member A	−0.86106
224143	Poglut1	protein O-glucosyltransferase 1	−0.67618
224613	Flywch1	FLYWCH-type zinc finger 1	0.952196
224792	Adgrf5	adhesion G protein-coupled receptor F5	−0.63765
225049	Ttc7	tetratricopeptide repeat domain 7	−0.73385
225131	Wac	WW domain containing adaptor with coiled-coil	0.615318
225443	Gm94	predicted gene 94	−3.59998
225908	Myrf	myelin regulatory factor	−1.22479
226075	Glis3	GLIS family zinc finger 3	0.663285
226245	Plekhs1	pleckstrin homology domain containing, family S member 1	−1.06638
226255	Atrnl1	attractin like 1	0.778521
226418	Yod1	YOD1 OTU deubiquitinating enzyme 1 homologue (S. cerevisiae)	−0.62753
226922	Kcnq5	potassium voltage-gated channel, subfamily Q, member 5	−0.88707
226970	Arhgef4	Rho guanine nucleotide exchange factor (GEF) 4	−1.07033
227721	Ppapdc3	phosphatidic acid phosphatase type 2 domain containing 3	−1.29695
228012	Tlk1	tousled-like kinase 1	0.613239
228355	Madd	MAP-kinase activating death domain	0.612579
228543	Rhov	ras homolog gene family, member V	−0.90042
228775	Trib3	tribbles homolog 3 (Drosophila)	−0.87301
228792	Dnmt3bos	DNA methyltransferase 3B, opposite strand	−0.74878
228796	Bpifb6	BPI fold containing family B, member 6	−0.70324
229542	Gatad2b	GATA zinc finger domain containing 2B	0.749205
229709	Ahcyl1	S-adenosylhomocysteine hydrolase-like 1	0.599917
229759	Olfm3	olfactomedin 3	−1.92722
230451	Junos	jun proto-oncogene, opposite strand	−0.93596
230603	Ttc39a	tetratricopeptide repeat domain 39A	0.662711
230613	Skint10	selection and upkeep of intraepithelial T cells 10	−0.95999
230861	Eif4g3	eukaryotic translation initiation factor 4 gamma, 3	0.870118
230971	Megf6	multiple EGF-like-domains 6	−0.58583
231002	Plekhn1	pleckstrin homology domain containing, family N member 1	−0.69277
231051	Kmt2c	lysine (K)-specific methyltransferase 2C	0.670753
231128	Fam193a	family with sequence similarity 193, member A	0.590958
231287	Atp10d	ATPase, class V, type 10D	−0.75598
231637	Ssh1	slingshot homolog 1 (Drosophila)	0.585963
231801	Agfg2	ArfGAP with FG repeats 2	0.964417
232035	Ccser1	coiled-coil serine rich 1	0.596191
232341	Wnk1	WNK lysine deficient protein kinase 1	0.849796
232415	Gm156	predicted gene 156	−2.43861
232599	Gm4876	predicted gene 4876	−0.74981
232943	Klc3	kinesin light chain 3	−1.21848
232959	Vmn1r178	vomeronasal 1 receptor 178	−1.0788
233544	A630091E08Rik	RIKEN cDNA A630091E08 gene	0.696576
233670	Olfr6	olfactory receptor 6	0.93882
233863	Gtf3c1	general transcription factor III C 1	0.596035
234023	Arglu1	arginine and glutamate rich 1	0.595255
234129	Tpte	transmembrane phosphatase with tensin homology	−0.85326
234724	Tat	tyrosine aminotransferase	−0.70604
234857	Spire2	spire homolog 2 (Drosophila)	0.747072
235028	Zfp426	zinc finger protein 426	0.655082
235504	Slc17a5	solute carrier family 17 (anion/sugar transporter), member 5	0.76319
235611	Plxnb1	plexin B1	−0.74232
235682	Zfp445	zinc finger protein 445	0.714018
236266	Alms1	Alstrom syndrome 1	0.616049
237611	Stac3	SH3 and cysteine rich domain 3	−0.95162
238021	Fscn2	fascin homolog 2, actin-bundling protein, retinal (Strongylocentrotus purpuratus)	−0.73853
238871	Pde4d	phosphodiesterase 4D, cAMP specific	0.709113
239410	A930017M01Rik	Smg-5 homolog, nonsense mediated mRNA decay factor pseudogene	−1.12958
239554	Foxred2	FAD-dependent oxidoreductase domain containing 2	−0.75763
239691	AU021092	expressed sequence AU021092	−0.92696
240411	Loxhd1	lipoxygenase homology domains 1	−1.19039
240505	Cdc42bpg	CDC42 binding protein kinase gamma (DMPK-like)	−0.59521
240518	Peli3	pellino 3	−0.84694
240638	Slc16a12	solute carrier family 16 (monocarboxylic acid transporters), member 12	−0.73791
240753	Plekha6	pleckstrin homology domain containing, family A member 6	0.827224
240755	4933406M09Rik	RIKEN cDNA 4933406M09 gene	−0.96799
241113	Prkag3	protein kinase, AMP-activated, gamma 3 non-catatlytic subunit	−0.99043
241201	Cdh7	cadherin 7, type 2	0.855924
241627	Wdr76	WD repeat domain 76	0.736018
242037	Ankub1	ankrin repeat and ubiquitin domain containing 1	−0.85464
242093	Rxfp4	relaxin family peptide receptor 4	−0.63316
242125	Mab21l3	mab-21-like 3 (C. elegans)	−2.39948
243535	BC048671	cDNA sequence BC048671	−1.60083
243819	Ppp6r1	protein phosphatase 6, regulatory subunit 1	0.678459
244199	Ovch2	ovochymase 2	−1.08592
244281	Myo16	myosin XVI	−1.16172
244332	Defb14	defensin beta 14	−2.80306
244886	AI118078	expressed sequence AI118078	−1.0719
245684	Cnksr2	connector enhancer of kinase suppressor of Ras 2	−0.84846
246081	Defb11	defensin beta 11	−1.10548
246735	AY074887	cDNA sequence AY074887	−0.99381
246788	Trpv3	transient receptor potential cation channel, subfamily V, member 3	1.11016
252837	Ackr4	atypical chemokine receptor 4	−0.97382
257885	Olfr885	olfactory receptor 885	−0.98794
257902	Olfr704	olfactory receptor 704	−1.09448
257912	Olfr948	olfactory receptor 948	−1.66079
258023	Olfr1306	olfactory receptor 1306	−1.3753
258025	Olfr1211	olfactory receptor 1211	−1.97936
258064	Olfr316	olfactory receptor 316	−0.64194
258177	Olfr1222	olfactory receptor 1222	−1.53601
258207	Olfr452	olfactory receptor 452	−1.35668
258290	Olfr1143	olfactory receptor 1143	−0.84815
258310	Olfr145	olfactory receptor 145	−0.83906
258326	Olfr642	olfactory receptor 642	−1.88474
258334	Olfr1396	olfactory receptor 1396	−1.12314
258335	Olfr374	olfactory receptor 374	−0.82561
258353	Olfr521	olfactory receptor 521	−0.88221
258359	Olfr1312	olfactory receptor 1312	−0.85416
258371	Olfr368	olfactory receptor 368	−1.53827
258390	Olfr1276	olfactory receptor 1276	−2.28313
258409	Olfr1431	olfactory receptor 1431	−1.85304
258417	Olfr470	olfactory receptor 470	−1.23992
258465	Olfr1387	olfactory receptor 1387	−0.84763
258477	Olfr197	olfactory receptor 197	0.727059
258492	Olfr484	olfactory receptor 484	1.013283
258494	Olfr318	olfactory receptor 318	−1.36443
258560	Olfr843	olfactory receptor 843	−1.4106
258590	Olfr702	olfactory receptor 702	1.771986
258616	Olfr357	olfactory receptor 357	−1.44533
258662	Olfr738	olfactory receptor 738	−1.18753
258674	Olfr1427	olfactory receptor 1427	−2.1351
258701	Olfr401	olfactory receptor 401	−0.88657
258730	Olfr483	olfactory receptor 483	−1.24945
258784	Olfr1245	olfactory receptor 1245	−1.52883
258800	Olfr905	olfactory receptor 905	−1.40489
258807	Olfr910	olfactory receptor 910	−1.42112
258808	Olfr620	olfactory receptor 620	−1.15739
258830	Olfr103	olfactory receptor 103	−1.16458
258879	Olfr330	olfactory receptor 330	1.320387
258880	Olfr218	olfactory receptor 218	−1.65817
258939	Olfr63	olfactory receptor 63	−1.23907
258976	Olfr1262	olfactory receptor 1262	−1.46816
259023	Olfr1055	olfactory receptor 1055	−1.46147
259041	Olfr1414	olfactory receptor 1414	−1.12738
259071	Olfr166	olfactory receptor 166	−1.67436
259082	Olfr1062	olfactory receptor 1062	−3.1017
259103	Olfr616	olfactory receptor 616	0.595395
259105	Olfr549	olfactory receptor 549	−0.79583
259277	Klk8	kallikrein related-peptidase 8	−1.69862
259279	Tubgcp3	tubulin, gamma complex associated protein 3	0.63937
268281	Shprh	SNF2 histone linker PHD RING helicase	0.603204
268482	Krt12	keratin 12	−1.4923
269023	Zfp608	zinc finger protein 608	0.771547
269643	Ppp2r2c	protein phosphatase 2, regulatory subunit B, gamma	−1.09993
270150	Ccdc153	coiled-coil domain containing 153	0.887061
270669	Mbtps2	membrane-bound transcription factor peptidase, site 2	−0.66564
271639	Adcy10	adenylate cyclase 10	1.048009
271697	Cdk15	cyclin-dependent kinase 15	−0.8656
272350	Gm5065	predicted gene 5065	0.948934
277343	Wfdc8	WAP four-disulfide core domain 8	0.725271
277899	Gm725	predicted gene 725	−1.17606
280645	B3gat2	beta-1,3-glucuronyltransferase 2 (glucuronosyltransferase S)	0.821913
319148	Hist1h3c	histone cluster 1, H3c	0.96292
319156	Hist1h4d	histone cluster 1, H4d	0.840808
319159	Hist1h4j	histone cluster 1, H4j	0.831783
319160	Hist1h4k	histone cluster 1, H4k	0.876777
319272	A130077B15Rik	RIKEN cDNA A130077B15 gene	0.635704
319336	C130036L24Rik	RIKEN cDNA C130036L24 gene	−1.1094
319565	Syne2	spectrin repeat containing, nuclear envelope 2	0.682204
319616	5930412G12Rik	RIKEN cDNA 5930412G12 gene	−0.67874
319638	Nt5dc1	5'-nucleotidase domain containing 1	−0.59093
319665	A430010J10Rik	RIKEN cDNA A430010J10 gene	−0.87303
319707	C430002N11Rik	RIKEN cDNA C430002N11 gene	−1.62649
319739	B230303O12Rik	RIKEN cDNA B230303O12 gene	−0.67322
319798	A730090N16Rik	RIKEN cDNA A730090N16 gene	−2.19574
319888	Oacyl	O-acyltransferase like	0.931066
319916	A730011C13Rik	RIKEN cDNA A730011C13 gene	−0.67345
319922	Vwc2	von Willebrand factor C domain containing 2	−0.81209
319939	Tns3	tensin 3	0.621004
320057	A630057N01Rik	RIKEN cDNA A630057N01 gene	0.856072
320099	BC106179	cDNA sequence BC106179	0.934393
320116	Fndc9	fibronectin type III domain containing 9	−1.50624
320176	D930032P07Rik	RIKEN cDNA D930032P07 gene	−1.76539
320206	A730028G07Rik	RIKEN cDNA A730028G07 gene	0.775949
320234	Ccdc66	coiled-coil domain containing 66	0.704543
320332	Hist4h4	histone cluster 4, H4	0.738735
320449	A730004F24Rik	RIKEN cDNA A730004F24 gene	−0.84895
320473	Heatr5b	HEAT repeat containing 5B	0.812605
320557	Fam169a	family with sequence similarity 169, member A	0.852969
320595	Phf8	PHD finger protein 8	0.632429
320718	Slc26a9	solute carrier family 26, member 9	−2.47693
320771	C030040A22Rik	RIKEN cDNA C030040A22 gene	1.001158
320790	Chd7	chromodomain helicase DNA binding protein 7	0.862642
320938	Tnpo3	transportin 3	0.795327
320939	5930403L14Rik	RIKEN cDNA 5930403L14 gene	−1.13108
327872	Gm12018	predicted gene 12018	−1.77223
328108	Fam179b	family with sequence similarity 179, member B	0.599925
328233	C230040D14	uncharacterized protein C230040D14	−0.84505
328699	Gabrr3	gamma-aminobutyric acid (GABA) receptor, rho 3	1.462313
328766	Gm5092	predicted gene 5092	−1.04859
328779	Hs3st6	heparan sulfate (glucosamine) 3-O-sulfotransferase 6	−2.36965
328789	Lhfpl5	lipoma HMGIC fusion partner-like 5	−0.66469
328971	Spink10	serine peptidase inhibitor, Kazal type 10	−1.22364
329387	C230014O12Rik	RIKEN cDNA C230014O12 gene	−0.6325
329509	1810024B03Rik	RIKEN cDNA 1810024B03 gene	−1.23083
329731	Fam19a3	family with sequence similarity 19, member A3	−0.80972
330662	Dock1	dedicator of cytokinesis 1	0.813496
331045	A530083I20Rik	RIKEN cDNA A530083I20 gene	−0.77418
331046	Tgm4	transglutaminase 4 (prostate)	−0.67338
331524	Xkrx	X Kell blood group precursor related X linked	−1.27333
331532	Tceal5	transcription elongation factor A (SII)-like 5	−1.08131
332131	Krt78	keratin 78	−2.62136
333639	Mamld1	mastermind-like domain containing 1	0.782401
338366	Mia3	melanoma inhibitory activity 3	0.718258
353188	Adam32	a disintegrin and metallopeptidase domain 32	−0.7638
378435	Mafa	v-maf musculoaponeurotic fibrosarcoma oncogene family, protein A (avian)	0.779909
380928	Lmo7	LIM domain only 7	0.91705
381269	Mreg	melanoregulin	−1.35993
381280	Hjurp	Holliday junction recognition protein	0.602615
381286	Serpinb3c	serine (or cysteine) peptidase inhibitor, clade B, member 3C	−2.74951
381373	Sp9	trans-acting transcription factor 9	−1.17358
381452	Gm1647	predicted gene 1647	0.798287
381476	Stpg2	sperm tail PG rich repeat containing 2	−1.11169
381598	2610005L07Rik	cadherin 11 pseudogene	0.702115
381628	Adgrf3	adhesion G protein-coupled receptor F3	−0.81046
382036	A530010L16Rik	RIKEN cDNA A530010L16 gene	−0.76642
382090	Cep162	centrosomal protein 162	0.632628
382522	Hist3h2bb-ps	histone cluster 3, H2bb, pseudogene	−1.45034
382551	Cd300lh	CD300 antigen like family member H	−0.63997
382793	Mtx3	metaxin 3	0.604577
384534	Vmn2r52	vomeronasal 2, receptor 52	−0.83778
386463	Cdsn	corneodesmosin	−2.69546
387565	Cd300c	CD300C antigen	−0.71718
399548	Scn4b	sodium channel, type IV, beta	−1.46963
404222	Olfr231	olfactory receptor 231	−1.24083
404311	Olfr209	olfactory receptor 209	−0.8033
404318	Olfr681	olfactory receptor 681	−1.02977
407803	BC051226	cDNA sequence BC051226	−0.60891
432637	Gm5433	predicted gene 5433	0.926254
433873	Gm9903	predicted gene 9903	1.215905
434147	D930028M14Rik	RIKEN cDNA D930028M14 gene	−0.91844
434285	BB014433	expressed sequence BB014433	0.739098
434440	Fbxw20	F-box and WD-40 domain protein 20	−1.38324
436336	Gm5767	predicted gene 5767	−1.24568
497097	Xkr4	X Kell blood group precursor related family member 4	0.980645
541610	Trcg1	taste receptor cell gene 1	−0.76876
544736	Glipr1l3	GLI pathogenesis-related 1 like 3	−0.96865
544881	BB287469	expressed sequence BB287469	−0.65289
545047	Gm5800	predicted gene 5800	−1.80319
545410	Fcnaos	ficolin A, opposite strand	−0.94578
545481	Arhgap40	Rho GTPase activating protein 40	−1.55664
545925	Psg27	pregnancy-specific glycoprotein 27	−1.00025
545934	Vmn1r173	vomeronasal 1 receptor 173	−0.75283
545975	Cers3	ceramide synthase 3	−2.88925
546801	Etv3l	ets variant 3-like	−0.86064
546886	Ccdc42b	coiled-coil domain containing 42B	−0.83395
546896	Olfr455	olfactory receptor 455	−0.65756
574415	Gm6042	type II keratin Kb32P	−1.20439
574417	Tas2r137	taste receptor, type 2, member 137	−1.33149
613264	1810020O05Rik	Riken cDNA 1810020O05 gene	−0.96643
619310	Zfp872	zinc finger protein 872	−1.29616
619548	Defb42	defensin beta 42	−1.09536
620760	2900079G21Rik	RIKEN cDNA 2900079G21 gene	−1.05197
622129	Gm6288	predicted gene 6288	−0.91767
622675	Zfp827	zinc finger protein 827	0.640512
623781	Gm14137	predicted gene 14137	−1.78948
624086	A230045G11Rik	RIKEN cDNA A230045G11 gene	0.763289
625243	Gm6567	predicted gene 6567	−0.74373
626848	Etohi1	ethanol induced 1	0.690213
627049	Zfp800	zinc finger protein 800	0.737874
628236	Lipo4	lipase, member O4	−1.19376
629554	Gm6980	predicted gene 6980	−2.05611
629754	Wfdc9	WAP four-disulfide core domain 9	−1.7709
630994	Lce3d	late cornified envelope 3D	−3.30253
633057	Gm7102	predicted gene 7102	0.833147
637053	Vmn2r4	vomeronasal 2, receptor 4	−1.06975
664609	Rhox4a	reproductive homeobox 4A	2.609269
664783	Dux	double homeobox	−1.46052
665001	Gm14391	predicted gene 14391	0.591605
665097	Gm7489	predicted gene 7489	−1.13701
665306	3930402G23Rik	RIKEN cDNA 3930402G23 gene	−0.67137
665998	Krtap4-9	keratin associated protein 4-9	−1.14971
666060	Frmpd1	FERM and PDZ domain containing 1	−0.77551
666168	Cyp4a31	cytochrome P450, family 4, subfamily a, polypeptide 31	−1.22105
666339	Muc3	mucin 3, intestinal	1.184079
667277	C1rb	complement component 1, r subcomponent B	0.724315
668311	Gm9099	predicted gene 9099	−0.76956
670558	H60c	histocompatibility 60c	−1.23452
670764	Vmn1r124	vomeronasal 1 receptor 124	−1.4086
670912	Gm9510	predicted gene 9510	0.593004
672284	Nkx1-1	NK1 transcription factor related, locus 1 (Drosophila)	0.823447
672511	Rnf213	ring finger protein 213	0.794514
675812	Zfp605	zinc finger protein 605	0.603514
675921	Tnk2os	tyrosine kinase, non-receptor 2, opposite strand	0.757281
791293	Gm9948	predicted gene 9948	−0.88202
791310	Gm10158	predicted gene 10158	0.844847
791415	Gm12500	predicted gene 12500	−0.66322
100036538	BC028777	cDNA sequence BC028777	−1.33134
100038358	Gm10857	predicted gene 10857	−1.23782
100038614	Gm10791	predicted gene 10791	−0.69418
100038680	Gm1971	predicted gene 1971	0.771704
100038691	Gm10344	predicted gene 10344	−1.05372
100038692	Gm10537	predicted gene 10537	−0.61348
100038730	Gm10387	predicted gene 10387	−0.9425
100038732	Gm11681	predicted gene 11681	−1.89885
100038740	Gm10325	predicted gene 10325	−1.03296
100038752	Gm10825	predicted gene 10825	−0.908
100038977	Gm1993	predicted gene 1993	−1.50013
100040491	Gm12349	predicted gene 12349	−1.032
100040852	Gm3002	alpha-takusan pseudogene	0.648214
100040902	Gm3032	predicted gene 3032	−1.2139
100040990	Gm3081	predicted gene 3081	0.618071
100042057	Gm3643	predicted gene 3643	−0.58699
100042499	Vmn2r55	vomeronasal 2, receptor 55	−0.76392
100042968	Vmn1r101	vomeronasal 1 receptor 101	−1.59822
100043072	A230070E04Rik	RIKEN cDNA A230070E04 gene	−0.75411
100043101	Vmn1r168	vomeronasal 1 receptor 168	−0.91621
100043229	Gm10046	predicted gene 10046	0.710773
100043255	Gm4319	predicted gene 4319	−1.05534
100043305	Gm4349	SET domain, bifurcated 1 pseudogene	0.851664
100043387	Gm14305	predicted gene 14305	0.585791
100043682	Gm10584	predicted gene 10584	−0.68142
100043765	Gm4632	predicted gene 4632	−0.71608
100047133	Gm16894	predicted gene, 16894	−0.8656
100047646	Gm17782	predicted gene, 17782	−0.97997
100049155	D930027P08Rik	RIKEN cDNA D930027P08 gene	0.880391
100125931	Phtf1os	putative homeodomain transcription factor 1, opposite strand	0.589855
100126202	9630029G12Rik	RIKEN cDNA 9630029G12 gene	−0.59159
100126224	Gm11747	predicted gene 11747	0.661016
100169876	Gm11123	predicted gene 11123	−0.66625
100306944	Snora73a	small nucleolar RNA, H/ACA box 73a	0.62275
100384890	D230030E09Rik	Riken cDNA D230030E09 gene	0.844222
100502593	Gm19269	predicted gene, 19269	0.956215
100502644	AA419673	expressed sequence AA419673	−0.80118
100502865	2300003K06Rik	RIKEN cDNA 2300003K06 gene	2.653412
100502868	Gm20597	predicted gene, 20597	0.994375
100502876	Kcnmb3	potassium large conductance calcium-activated channel, subfamily M, beta member 3	−1.46165
100503085	Klhl3	kelch-like 3	−1.10797
100503185	Btbd8	BTB (POZ) domain containing 8	0.849817
100503323	A230087F16Rik	RIKEN cDNA A230087F16 gene	0.685612
100503357	AW060742	expressed sequence AW060742	−1.20884
100503384	Gm19665	predicted gene, 19665	1.369618
100503460	Gm19705	predicted gene, 19705	−0.68039
100503469	Gm19710	predicted gene, 19710	−1.05228
100503609	Lnp1	leukemia NUP98 fusion partner 1	−1.36631
100503630	Gm12002	predicted gene 12002	−0.73483
100503704	Gm16861	predicted gene, 16861	−0.73992
100504169	Gm20098	predicted gene, 20098	−1.08526
100504453	Gm20236	predicted gene, 20236	−1.16211
100504464	E230016K23Rik	RIKEN cDNA E230016K23 gene	−0.78439
100504561	9230112J17Rik	RIKEN cDNA 9230112J17 gene	−0.6298
100504569	Gm13205	predicted gene 13205	0.769604
100504650	A730085E03Rik	RIKEN cDNA A730085E03 gene	−0.7639
100862431	LOC100862431	H-2 class I histocompatibility antigen, D-D alpha chain-like	0.652366
102631545	Gm29856	predicted gene, 29856	−1.19714
102631791	Gm11465	predicted gene 11465	−0.85102
102631866	Gm30092	predicted gene, 30092	−0.65396
102631991	Gm30178	predicted gene, 30178	−1.78287
102631997	Gm12589	predicted gene 12589	−0.75224
102632193	Gm30333	predicted gene, 30333	−1.19779
102632388	Gm38444	predicted gene, 38444	0.65274
102632891	Gm30848	predicted gene, 30848	1.270809
102633315	Gm16159	predicted gene 16159	0.753177
102633555	Gm14051	predicted gene 14051	0.818671
102633705	LOC102633705	uncharacterized LOC102633705	−1.3771
102633711	Gm15320	predicted gene 15320	−0.99499
102633793	Gm31534	predicted gene, 31534	−0.65
102633805	Gm26688	predicted gene, 26688	−2.96899
102634491	Gm32062	predicted gene, 32062	−2.39892
102634598	Gm32139	predicted gene, 32139	−1.56876
102634642	Gm32178	predicted gene, 32178	0.99884
102634982	Gm32431	predicted gene, 32431	−1.60357
102635494	Gm12576	predicted gene 12576	0.948289
102635495	Gm32817	predicted gene, 32817	−0.72467
102635553	Gm15624	predicted gene 15624	0.706913
102635587	Gm32880	predicted gene, 32880	1.193606
102635639	Gm32926	predicted gene, 32926	−0.98054
102635721	Gm26651	predicted gene, 26651	−1.15859
102635799	Gm33047	predicted gene, 33047	0.852917
102635801	LOC102635801	uncharacterized LOC102635801	1.789109
102636085	Gm33255	predicted gene, 33255	−1.98616
102636217	Gm26806	predicted gene, 26806	−1.31753
102636463	Gm38488	predicted gene, 38488	0.795664
102637189	Gm34066	predicted gene, 34066	−1.6205
102637485	Gm34280	predicted gene, 34280	−0.81988
102637558	Gm34336	predicted gene, 34336	−3.93697
102638051	Gm13657	predicted gene 13657	−1.31116
102638077	Gm34726	predicted gene, 34726	−1.34647
102638234	Gm34843	predicted gene, 34843	0.901377
102638282	Gm34883	predicted gene, 34883	−0.64989
102638285	Gm13415	predicted gene 13415	−0.83406
102638424	Gm34995	predicted gene, 34995	−1.01039
102638584	Gm35113	predicted gene, 35113	−1.50116
102638626	Gm35147	predicted gene, 35147	0.92129
102638630	Gm26779	predicted gene, 26779	0.856981
102638651	Gm35164	predicted gene, 35164	0.755207
102638667	Gm35177	predicted gene, 35177	−1.88393
102638784	Gm35266	predicted gene, 35266	1.049232
102638917	Gm35363	predicted gene, 35363	0.982775
102639110	Gm35501	predicted gene, 35501	−1.42978
102639139	Gm35522	predicted gene, 35522	−0.64853
102639228	Gm35587	predicted gene, 35587	−0.89923
102639958	LOC102639958	uncharacterized LOC102639958	−0.75044
102639962	Gm36151	predicted gene, 36151	0.78053
102639996	Gm36177	predicted gene, 36177	−1.41141
102640128	Gm36269	predicted gene, 36269	−1.375
102640184	Gm36313	predicted gene, 36313	−0.87374
102640241	LOC102640241	uncharacterized LOC102640241	0.710842
102640255	Gm36367	predicted gene, 36367	0.827981
102640379	Gm11716	predicted gene 11716	−1.01706
102640449	Tvp23bos	trans-golgi network vesicle protein 23B, opposite strand	−0.63835
102640521	Gm36561	predicted gene, 36561	−0.67657
102640562	LOC102640562	uncharacterized LOC102640562	−1.38291
102640826	Gm36802	predicted gene, 36802	−1.18872
102641119	LOC102641119	uncharacterized LOC102641119	0.878519
102641261	LOC102641261	uncharacterized LOC102641261	0.984663
102641428	LOC102641428	uncharacterized LOC102641428	0.977322
102641783	Gm38575	predicted gene, 38575	−1.45318
102641978	LOC102641978	uncharacterized LOC102641978	0.673717
102642140	LOC102642140	uncharacterized LOC102642140	1.357604
102642271	LOC102642271	calcium-binding and coiled-coil domain-containing protein 2-like	0.880414
102642579	LOC102642579	cyclic AMP-responsive element-binding protein 3-like protein 2 pseudogene	0.711796
102642951	LOC102642951	uncharacterized LOC102642951	0.901922
105242587	Gm38758	predicted gene, 38758	−0.61912
105242792	Adap2os	ArfGAP with dual PH domains 2, opposite strand	−2.0222
105243282	LOC105243282	uncharacterized LOC105243282	−1.01203
105244026	Gm38293	predicted gene, 38293	0.621271
105245424	Gm40881	predicted gene, 40881	0.810625
105246057	LOC105246057	igE-binding protein-like	0.622452
105247377	LOC105247377	uncharacterized LOC105247377	−0.66635

**Table 3:** Differentially expressed genes (DEG) in the colon of mice after 14 days of exposure to E171.

**Entrez gene ID**	**Gene symbol**	**Gene name**	**Log2FC**
11421	Ace	angiotensin I converting enzyme (peptidyl-dipeptidase A) 1	0.911786
11480	Acvr2a	activin receptor IIA	−0.59046
11499	Adam5	a disintegrin and metallopeptidase domain 5	−1.04683
11513	Adcy7	adenylate cyclase 7	−0.68104
11520	Plin2	perilipin 2	0.620984
11542	Adora3	adenosine A3 receptor	−0.80428
11553	Adra2c	adrenergic receptor, alpha 2c	−0.70549
11652	Akt2	thymoma viral proto-oncogene 2	1.261177
11731	Ang2	angiogenin, ribonuclease A family, member 2	−2.80064
11765	Ap1g1	adaptor protein complex AP-1, gamma 1 subunit	0.700875
11803	Aplp1	amyloid beta (A4) precursor-like protein 1	0.649492
11834	Aqr	aquarius	−0.70336
11856	Arhgap6	Rho GTPase activating protein 6	−1.036
11859	Phox2a	paired-like homeobox 2a	−1.00702
11977	Atp7a	ATPase, Cu++ transporting, alpha polypeptide	−0.78423
12013	Bach1	BTB and CNC homology 1	−0.58678
12029	Bcl6b	B cell CLL/lymphoma 6, member B	−0.71772
12036	Bcat2	branched chain aminotransferase 2, mitochondrial	0.609443
12041	Bckdk	branched chain ketoacid dehydrogenase kinase	0.610643
12042	Bcl10	B cell leukemia/lymphoma 10	−0.60308
12047	Bcl2a1d	B cell leukemia/lymphoma 2 related protein A1d	−0.98334
12051	Bcl3	B cell leukemia/lymphoma 3	−0.59878
12053	Bcl6	B cell leukemia/lymphoma 6	−0.63678
12111	Bgn	biglycan	−0.78843
12164	Bmp8b	bone morphogenetic protein 8b	−1.65646
12193	Zfp36l2	zinc finger protein 36, C3H type-like 2	−0.72839
12270	C4bp-ps1	complement component 4 binding protein, pseudogene 1	−0.82253
12273	C5ar1	complement component 5a receptor 1	−1.12916
12290	Cacna1e	calcium channel, voltage-dependent, R type, alpha 1E subunit	−0.66657
12291	Cacna1g	calcium channel, voltage-dependent, T type, alpha 1G subunit	−1.42502
12326	Camk4	calcium/calmodulin-dependent protein kinase IV	−1.12934
12395	Runx1t1	runt-related transcription factor 1; translocated to, 1 (cyclin D-related)	−0.72272
12400	Cbfb	core binding factor beta	−0.60334
12412	Cbx1	chromobox 1	−0.89405
12445	Ccnd3	cyclin D3	−0.59227
12448	Ccne2	cyclin E2	−1.32048
12453	Ccni	cyclin I	0.645054
12506	Cd48	CD48 antigen	−0.83964
12523	Cd84	CD84 antigen	−1.14485
12524	Cd86	CD86 antigen	−1.08973
12575	Cdkn1a	cyclin-dependent kinase inhibitor 1A (P21)	0.821051
12631	Cfl1	cofilin 1, non-muscle	0.962626
12704	Cit	citron	1.21133
12752	Cln3	ceroid lipofuscinosis, neuronal 3, juvenile (Batten, Spielmeyer-Vogt disease)	0.654024
12772	Ccr2	chemokine (C-C motif) receptor 2	−1.20079
12804	Cntfr	ciliary neurotrophic factor receptor	−0.9379
12812	Coil	coilin	−0.6788
12819	Col15a1	collagen, type XV, alpha 1	0.805905
12827	Col4a2	collagen, type IV, alpha 2	0.890455
12961	Crybb2	crystallin, beta B2	−0.59977
12977	Csf1	colony stimulating factor 1 (macrophage)	−0.85002
13058	Cybb	cytochrome b-245, beta polypeptide	−1.07607
13132	Dab2	disabled 2, mitogen-responsive phosphoprotein	−1.91221
13169	Dbnl	drebrin-like	0.660419
13171	Dbt	dihydrolipoamide branched chain transacylase E2	−0.61663
13172	Dbx1	developing brain homeobox 1	−0.98763
13178	Dck	deoxycytidine kinase	−0.6267
13190	Dct	dopachrome tautomerase	−1.01545
13367	Diap1	diaphanous homolog 1 (Drosophila)	1.324411
13400	Dmpk	dystrophia myotonica-protein kinase	0.9284
13426	Dync1i1	dynein cytoplasmic 1 intermediate chain 1	−0.76492
13430	Dnm2	dynamin 2	0.6927
13498	Atn1	atrophin 1	0.605124
13508	Dscam	Down syndrome cell adhesion molecule	−0.74569
13518	Dst	dystonin	0.65511
13532	Usp17lc	ubiquitin specific peptidase 17-like C	0.755766
13586	Ear1	eosinophil-associated, ribonuclease A family, member 1	−0.9434
13618	Ednrb	endothelin receptor type B	0.93025
13629	Eef2	eukaryotic translation elongation factor 2	0.637698
13655	Egr3	early growth response 3	−2.15349
13665	Eif2s1	eukaryotic translation initiation factor 2, subunit 1 alpha	−0.63654
13666	Eif2ak3	eukaryotic translation initiation factor 2 alpha kinase 3	−0.68318
13733	Adgre1	adhesion G protein-coupled receptor E1	−0.73302
13806	Eno1	enolase 1, alpha non-neuron	0.603135
13835	Epha1	Eph receptor A1	−0.97562
13982	Esr1	estrogen receptor 1 (alpha)	−0.68068
13990	Smarcad1	SWI/SNF-related, matrix-associated actin-dependent regulator of chromatin, subfamily a, containing DEAD/H box 1	−0.59761
14017	Evi2a	ecotropic viral integration site 2a	−1.06606
14055	Ezh1	enhancer of zeste homolog 1 (Drosophila)	0.622265
14105	Srsf10	serine/arginine-rich splicing factor 10	−0.59777
14129	Fcgr1	Fc receptor, IgG, high affinity I	−0.82062
14130	Fcgr2b	Fc receptor, IgG, low affinity IIb	−0.78621
14190	Fgl2	fibrinogen-like protein 2	−0.77933
14202	Fhl4	four and a half LIM domains 4	−0.74795
14238	Foxf2	forkhead box F2	−0.9202
14247	Fli1	Friend leukemia integration 1	−1.08505
14248	Flii	flightless I homolog (Drosophila)	0.678156
14347	Fut7	fucosyltransferase 7	−1.19874
14371	Fzd9	frizzled homolog 9 (Drosophila)	−1.62967
14387	Gaa	glucosidase, alpha, acid	0.816403
14395	Gabra2	gamma-aminobutyric acid (GABA) A receptor, subunit alpha 2	−2.09585
14406	Gabrg2	gamma-aminobutyric acid (GABA) A receptor, subunit gamma 2	1.85988
14453	Gas2	growth arrest specific 2	−0.77701
14460	Gata1	GATA binding protein 1	−1.43685
14528	Gch1	GTP cyclohydrolase 1	−0.71103
14536	Nr6a1	nuclear receptor subfamily 6, group A, member 1	−0.67133
14537	Gcnt1	glucosaminyl (N-acetyl) transferase 1, core 2	−1.25441
14559	Gdf1	growth differentiation factor 1	−0.63515
14566	Gdf9	growth differentiation factor 9	−0.88193
14567	Gdi1	guanosine diphosphate (GDP) dissociation inhibitor 1	0.625394
14573	Gdnf	glial cell line derived neurotrophic factor	−0.92243
14602	Ghrhr	growth hormone releasing hormone receptor	0.587572
14610	Gja10	gap junction protein, alpha 10	1.249212
14615	Gjc1	gap junction protein, gamma 1	−0.62925
14628	Ostm1	osteopetrosis associated transmembrane protein 1	−0.61576
14654	Glra1	glycine receptor, alpha 1 subunit	0.824164
14670	Gnl1	guanine nucleotide binding protein-like 1	−0.69678
14705	Bscl2	Berardinelli-Seip congenital lipodystrophy 2 homolog (seipin)	0.625182
14739	S1pr2	sphingosine-1-phosphate receptor 2	−0.762
14744	Gpr65	G-protein coupled receptor 65	−1.13336
14765	Gpr50	G-protein-coupled receptor 50	0.985069
14772	Grk4	G protein-coupled receptor kinase 4	−1.02692
14886	Gtf2i	general transcription factor II I	0.632871
14904	Gtpbp1	GTP binding protein 1	0.678717
14940	Gzmc	granzyme C	−1.27284
15162	Hck	hemopoietic cell kinase	−1.01759
15205	Hes1	hairy and enhancer of split 1 (Drosophila)	−1.35713
15213	Hey1	hairy/enhancer-of-split related with YRPW motif 1	−0.7366
15242	Hhex	hematopoietically expressed homeobox	−1.16279
15257	Hipk1	homeodomain interacting protein kinase 1	−0.64974
15260	Hira	histone cell cycle regulation defective homolog A (S. cerevisiae)	0.643582
15277	Hk2	hexokinase 2	−1.11475
15365	Hmga2-ps1	high mobility group AT-hook 2, pseudogene 1	−0.99581
15394	Hoxa1	homeobox A1	−1.2248
15399	Hoxa2	homeobox A2	−0.86133
15401	Hoxa4	homeobox A4	0.807245
15478	Hs3st3a1	heparan sulfate (glucosamine) 3-O-sulfotransferase 3A1	−0.81603
15551	Htr1b	5-hydroxytryptamine (serotonin) receptor 1B	−1.07636
15565	Htr6	5-hydroxytryptamine (serotonin) receptor 6	1.237737
15566	Htr7	5-hydroxytryptamine (serotonin) receptor 7	−1.20957
15932	Idua	iduronidase, alpha-L-	0.700167
15939	Ier5	immediate early response 5	−0.62746
15964	Ifna11	interferon alpha 11	−1.54094
16154	Il10ra	interleukin 10 receptor, alpha	−0.86002
16182	Il18r1	interleukin 18 receptor 1	−1.19697
16192	Il5ra	interleukin 5 receptor, alpha	−0.89021
16348	Invs	inversin	−0.58624
16434	Itpa	inosine triphosphatase (nucleoside triphosphate pyrophosphatase)	−0.80611
16449	Jag1	jagged 1	−0.61725
16504	Kcnc3	potassium voltage gated channel, Shaw-related subfamily, member 3	0.632554
16554	Kif13b	kinesin family member 13B	0.672891
16598	Klf2	Kruppel-like factor 2 (lung)	−0.91807
16627	Klra1	killer cell lectin-like receptor, subfamily A, member 1	−1.54136
16639	Klra8	killer cell lectin-like receptor, subfamily A, member 8	−1.11264
16658	Mafb	v-maf musculoaponeurotic fibrosarcoma oncogene family, protein B (avian)	−0.74396
16681	Krt2	keratin 2	0.702325
16728	L1cam	L1 cell adhesion molecule	0.712046
16825	Ldb1	LIM domain binding 1	0.607098
16874	Lhx6	LIM homeobox protein 6	0.906306
16878	Lif	leukemia inhibitory factor	−0.96284
16977	Lrrc23	leucine rich repeat containing 23	−0.80253
16993	Lta4h	leukotriene A4 hydrolase	−0.77169
17101	Lyst	lysosomal trafficking regulator	−0.82374
17110	Lyz1	lysozyme 1	−1.11301
17164	Mapkapk2	MAP kinase-activated protein kinase 2	−0.68625
17188	Maz	MYC-associated zinc finger protein (purine-binding transcription factor)	0.74489
17240	Mdfi	MyoD family inhibitor	−0.98588
17254	Slc3a2	solute carrier family 3 (activators of dibasic and neutral amino acid transport), member 2	0.82047
17312	Clec10a	C-type lectin domain family 10, member A	−1.13626
17364	Trpm1	transient receptor potential cation channel, subfamily M, member 1	−0.91785
17389	Mmp16	matrix metallopeptidase 16	−1.0012
17706	ATP8	ATP synthase F0 subunit 8	0.691655
17716	ND1	NADH dehydrogenase subunit 1	0.590994
17720	ND4L	NADH dehydrogenase subunit 4L	0.725803
17721	ND5	NADH dehydrogenase subunit 5	0.797087
17762	Mapt	microtubule-associated protein tau	−0.77412
17777	Mttp	microsomal triglyceride transfer protein	−0.71893
17880	Myh11	myosin, heavy polypeptide 11, smooth muscle	1.047129
17910	Myo15	myosin XV	−1.22989
17918	Myo5a	myosin VA	−0.71429
17933	Myt1l	myelin transcription factor 1-like	−1.12454
17954	Nap1l2	nucleosome assembly protein 1-like 2	−1.48339
17962	Nat3	N-acetyltransferase 3	−2.61957
17999	Nedd4	neural precursor cell expressed, developmentally down-regulated 4	−0.68405
18004	Nek1	NIMA (never in mitosis gene a)-related expressed kinase 1	−0.59819
18080	Nin	ninein	−0.92448
18104	Nqo1	NAD(P)H dehydrogenase, quinone 1	−0.8429
18109	Mycn	v-myc myelocytomatosis viral related oncogene, neuroblastoma derived (avian)	−0.91148
18198	Musk	muscle, skeletal, receptor tyrosine kinase	−0.95657
18208	Ntn1	netrin 1	−0.91143
18214	Ddr2	discoidin domain receptor family, member 2	0.740068
18223	Numbl	numb-like	−0.8831
18307	Olfr10	olfactory receptor 10	0.993785
18392	Orc1	origin recognition complex, subunit 1	−0.65765
18432	Mybbp1a	MYB binding protein (P160) 1a	0.819548
18439	P2rx7	purinergic receptor P2X, ligand-gated ion channel, 7	−1.32629
18452	P4ha2	procollagen-proline, 2-oxoglutarate 4-dioxygenase (proline 4-hydroxylase), alpha II polypeptide	−0.86494
18504	Pax2	paired box 2	0.802788
18591	Pdgfb	platelet derived growth factor, B polypeptide	−0.73059
18595	Pdgfra	platelet derived growth factor receptor, alpha polypeptide	−0.79464
18604	Pdk2	pyruvate dehydrogenase kinase, isoenzyme 2	0.787127
18637	Pfdn2	prefoldin 2	−0.61542
18640	Pfkfb2	6-phosphofructo-2-kinase/fructose-2,6-biphosphatase 2	−0.73418
18679	Phka1	phosphorylase kinase alpha 1	−2.34329
18706	Pik3ca	phosphatidylinositol 3-kinase, catalytic, alpha polypeptide	−1.03111
18708	Pik3r1	phosphatidylinositol 3-kinase, regulatory subunit, polypeptide 1 (p85 alpha)	0.668175
18709	Pik3r2	phosphatidylinositol 3-kinase, regulatory subunit, polypeptide 2 (p85 beta)	0.590647
18733	Pirb	paired Ig-like receptor B	−0.88798
18779	Pla2r1	phospholipase A2 receptor 1	−0.62708
18786	Plaa	phospholipase A2, activating protein	−0.94444
18810	Plec	plectin	0.588317
18830	Pltp	phospholipid transfer protein	1.179872
18996	Pou4f1	POU domain, class 4, transcription factor 1	−1.06055
19042	Ppm1a	protein phosphatase 1A, magnesium dependent, alpha isoform	−0.62283
19069	Nup88	nucleoporin 88	−0.61846
19076	Prim2	DNA primase, p58 subunit	−0.76832
19143	St14	suppression of tumorigenicity 14 (colon carcinoma)	0.601419
19206	Ptch1	patched homolog 1	1.05912
19211	Pten	phosphatase and tensin homolog	−0.65603
19222	Ptgir	prostaglandin I receptor (IP)	−1.06318
19241	Tmsb4x	thymosin, beta 4, X chromosome	−0.69182
19260	Ptpn22	protein tyrosine phosphatase, non-receptor type 22 (lymphoid)	−1.3422
19261	Sirpa	signal-regulatory protein alpha	−0.86607
19267	Ptpre	protein tyrosine phosphatase, receptor type, E	−0.80866
19274	Ptprm	protein tyrosine phosphatase, receptor type, M	−0.95632
19296	Pvt1	plasmacytoma variant translocation 1	−0.99522
19326	Rab11b	RAB11B, member RAS oncogene family	0.842466
19358	Rad23a	RAD23a homolog (S. cerevisiae)	0.906769
19387	Rangap1	RAN GTPase activating protein 1	0.793977
19671	Rce1	RCE1 homolog, prenyl protein peptidase (S. cerevisiae)	−0.6925
19679	Pitpnm2	phosphatidylinositol transfer protein, membrane-associated 2	0.656582
19713	Ret	ret proto-oncogene	−0.85647
20020	Polr2a	polymerase (RNA) II (DNA directed) polypeptide A	0.609676
20163	Rsu1	Ras suppressor protein 1	0.95159
20293	Ccl12	chemokine (C-C motif) ligand 12	−0.84754
20301	Ccl27a	chemokine (C-C motif) ligand 27A	−0.74211
20302	Ccl3	chemokine (C-C motif) ligand 3	−2.21765
20306	Ccl7	chemokine (C-C motif) ligand 7	−1.10314
20352	Sema4b	sema domain, immunoglobulin domain (Ig), transmembrane domain (TM) and short cytoplasmic domain, (semaphorin) 4B	−0.6176
20409	Ostf1	osteoclast stimulating factor 1	−0.6474
20411	Sorbs1	sorbin and SH3 domain containing 1	0.827534
20430	Cyfip1	cytoplasmic FMR1 interacting protein 1	0.947959
20439	Siah2	seven in absentia 2	−0.61219
20443	St3gal4	ST3 beta-galactoside alpha-2,3-sialyltransferase 4	−1.00668
20522	Slc23a1	solute carrier family 23 (nucleobase transporters), member 1	−0.76077
20535	Slc4a2	solute carrier family 4 (anion exchanger), member 2	0.660713
20583	Snai2	snail family zinc finger 2	−0.99509
20586	Smarca4	SWI/SNF related, matrix associated, actin dependent regulator of chromatin, subfamily a, member 4	0.754049
20587	Smarcb1	SWI/SNF related, matrix associated, actin dependent regulator of chromatin, subfamily b, member 1	−0.82292
20616	Snap91	synaptosomal-associated protein 91	−1.3523
20663	Sos2	son of sevenless homolog 2 (Drosophila)	−0.65643
20668	Sox13	SRY (sex determining region Y)-box 13	0.593157
20678	Sox5	SRY (sex determining region Y)-box 5	−1.24779
20682	Sox9	SRY (sex determining region Y)-box 9	0.74219
20688	Sp4	trans-acting transcription factor 4	−0.72986
20728	Spic	Spi-C transcription factor (Spi-1/PU.1 related)	−1.05292
20737	Spn	sialophorin	−1.15893
20750	Spp1	secreted phosphoprotein 1	−2.51359
20779	Src	Rous sarcoma oncogene	0.798342
20821	Trim21	tripartite motif-containing 21	−0.59109
20849	Stat4	signal transducer and activator of transcription 4	−1.55203
20897	Stra6	stimulated by retinoic acid gene 6	−1.73208
20917	Suclg2	succinate-Coenzyme A ligase, GDP-forming, beta subunit	0.820739
20926	Supt6	suppressor of Ty 6	0.622045
20979	Syt1	synaptotagmin I	−0.65486
20981	Syt3	synaptotagmin III	0.627271
21337	Tacr2	tachykinin receptor 2	0.87752
21349	Tal1	T cell acute lymphocytic leukemia 1	−0.60698
21379	Tbrg4	transforming growth factor beta regulated gene 4	0.651441
21426	Tfec	transcription factor EC	−1.75512
21427	Vps72	vacuolar protein sorting 72 (yeast)	0.636153
21665	Tdg	thymine DNA glycosylase	−0.91211
21819	Tg	thyroglobulin	−0.84227
21833	Thra	thyroid hormone receptor alpha	0.631708
21917	Tmpo	thymopoietin	−0.66021
21930	Tnfaip6	tumor necrosis factor alpha induced protein 6	−0.8083
21942	Tnfrsf9	tumor necrosis factor receptor superfamily, member 9	−1.41051
21943	Tnfsf11	tumor necrosis factor (ligand) superfamily, member 11	−1.93816
21946	Pglyrp1	peptidoglycan recognition protein 1	0.814414
21950	Tnfsf9	tumor necrosis factor (ligand) superfamily, member 9	−0.82536
21951	Tnks	tankyrase, TRF1-interacting ankyrin-related ADP-ribose polymerase	0.718512
21983	Tpbg	trophoblast glycoprotein	−0.91729
22022	Tpst2	protein-tyrosine sulfotransferase 2	−0.76378
22059	Trp53	transformation related protein 53	0.876668
22068	Trpc6	transient receptor potential cation channel, subfamily C, member 6	−0.92416
22113	Phlda2	pleckstrin homology-like domain, family A, member 2	−1.71736
22123	Psmd3	proteasome (prosome, macropain) 26S subunit, non-ATPase, 3	0.785903
22141	Tub	tubby candidate gene	−0.73882
22142	Tuba1a	tubulin, alpha 1A	−0.60318
22165	Txk	TXK tyrosine kinase	−1.63873
22174	Tyro3	TYRO3 protein tyrosine kinase 3	−0.60664
22200	Uba3	ubiquitin-like modifier activating enzyme 3	−0.75795
22201	Uba1	ubiquitin-like modifier activating enzyme 1	0.651076
22213	Ube2g2	ubiquitin-conjugating enzyme E2G 2	−0.69422
22228	Ucp2	uncoupling protein 2 (mitochondrial, proton carrier)	0.870627
22229	Ucp3	uncoupling protein 3 (mitochondrial, proton carrier)	0.784513
22258	Usp4	ubiquitin specific peptidase 4 (proto-oncogene)	−0.68226
22260	Nr1h2	nuclear receptor subfamily 1, group H, member 2	0.604991
22321	Vars	valyl-tRNA synthetase	0.645143
22359	Vldlr	very low density lipoprotein receptor	−0.66092
22363	Vpreb2	pre-B lymphocyte gene 2	−1.24648
22378	Wbp2	WW domain binding protein 2	0.628657
22401	Zmat3	zinc finger matrin type 3	−0.858
22420	Wnt6	wingless-type MMTV integration site family, member 6	−0.65279
22439	Xk	Kell blood group precursor (McLeod phenotype) homolog	−0.65043
22441	Xlr	X-linked lymphocyte-regulated	−0.71592
22691	Zscan2	zinc finger and SCAN domain containing 2	0.610053
22693	Zfp30	zinc finger protein 30	−0.68597
22700	Zfp40	zinc finger protein 40	−0.59553
22710	Zfp52	zinc finger protein 52	−1.00494
22715	Zfp57	zinc finger protein 57	−1.40029
22750	Zfp9	zinc finger protein 9	−1.25677
23890	Gpr34	G protein-coupled receptor 34	−0.81039
23965	Tenm3	teneurin transmembrane protein 3	−1.00709
23989	Med24	mediator complex subunit 24	0.629217
24004	Rai2	retinoic acid induced 2	−0.86776
24017	Rnf13	ring finger protein 13	−0.66811
24044	Scamp2	secretory carrier membrane protein 2	0.92345
24055	Sh3bp2	SH3-domain binding protein 2	−0.71473
24056	Sh3bp5	SH3-domain binding protein 5 (BTK-associated)	−0.63987
24100	Tpra1	transmembrane protein, adipocyte asscociated 1	0.657648
24113	Vax2	ventral anterior homeobox 2	−1.51845
24116	Nelfa	negative elongation factor complex member A, Whsc2	−0.7588
24132	Zfp53	zinc finger protein 53	−0.60066
26428	Orc4	origin recognition complex, subunit 4	−0.88981
26445	Psmb2	proteasome (prosome, macropain) subunit, beta type 2	−0.62388
26448	Mok	MOK protein kinase	−0.73433
26457	Slc27a1	solute carrier family 27 (fatty acid transporter), member 1	0.710861
26570	Slc7a11	solute carrier family 7 (cationic amino acid transporter, y+ system), member 11	−1.84392
26874	Abcd2	ATP-binding cassette, sub-family D (ALD), member 2	−2.35733
26877	B3galt1	UDP-Gal:betaGlcNAc beta 1,3-galactosyltransferase, polypeptide 1	−0.90065
26904	Sh2d1b1	SH2 domain containing 1B1	−2.90015
26914	H2afy	H2A histone family, member Y	0.594298
26934	Racgap1	Rac GTPase-activating protein 1	1.040865
26936	Mprip	myosin phosphatase Rho interacting protein	0.811936
26939	Polr3e	polymerase (RNA) III (DNA directed) polypeptide E	−0.64162
27028	Ermap	erythroblast membrane-associated protein	0.658658
27055	Fkbp9	FK506 binding protein 9	−0.64779
27083	Xlr4b	X-linked lymphocyte-regulated 4B	−1.16424
27401	Skp2	S-phase kinase-associated protein 2 (p45)	−0.8318
27962	D9Wsu90e	DNA segment, Chr 9, Wayne State University 90, expressed	−1.27352
28006	Fam21	family with sequence similarity 21	0.79424
28064	Yipf3	Yip1 domain family, member 3	0.638926
28077	Med10	mediator complex subunit 10	−0.68093
28114	Nsun2	NOL1/NOP2/Sun domain family member 2	0.669351
28135	Cep63	centrosomal protein 63	0.911453
29810	Bag3	BCL2-associated athanogene 3	−0.74136
29865	Cabp5	calcium binding protein 5	−0.63451
30052	Pcsk1n	proprotein convertase subtilisin/kexin type 1 inhibitor	0.726871
30785	Cttnbp2	cortactin binding protein 2	−0.69114
30840	Fbxl6	F-box and leucine-rich repeat protein 6	−0.77752
30853	Mlf2	myeloid leukemia factor 2	0.678133
30928	Zbtb18	zinc finger and BTB domain containing 18	−0.61872
30951	Cbx8	chromobox 8	−0.61326
50492	Thop1	thimet oligopeptidase 1	−1.47365
50781	Dkk3	dickkopf homolog 3 (Xenopus laevis)	0.666643
50876	Tmod2	tropomodulin 2	−1.03941
50878	Stag3	stromal antigen 3	−0.79787
50912	Exosc10	exosome component 10	0.593462
50928	Klrg1	killer cell lectin-like receptor subfamily G, member 1	−0.94962
51869	Rif1	Rap1 interacting factor 1 homolog (yeast)	−0.60659
51873	D2Ertd127e	DNA segment, Chr 2, ERATO Doi 127, expressed	−0.61571
52009	Hn1l	hematological and neurological expressed 1-like	−0.73126
52076	Tmem38b	transmembrane protein 38B	−1.71186
52250	Reep1	receptor accessory protein 1	−0.76504
52357	Wwc2	WW, C2 and coiled-coil domain containing 2	−0.59555
52409	D5Ertd798e	DNA segment, Chr 5, ERATO Doi 798, expressed	−1.18975
52480	Snhg14	small nucleolar RNA host gene 14	−0.8668
52679	E2f7	E2F transcription factor 7	0.611548
52855	Lair1	leukocyte-associated Ig-like receptor 1	−1.02348
52892	Sco1	SCO cytochrome oxidase deficient homolog 1 (yeast)	−0.84059
53325	Banp	BTG3 associated nuclear protein	−0.87587
53421	Sec61a1	Sec61 alpha 1 subunit (S. cerevisiae)	0.620013
53602	Hpcal1	hippocalcin-like 1	0.608778
53614	Reck	reversion-inducing-cysteine-rich protein with kazal motifs	−0.59103
53761	Prrc2a	proline-rich coiled-coil 2A	0.774422
53871	Pkd2l2	polycystic kidney disease 2-like 2	0.816075
53872	Caprin1	cell cycle associated protein 1	−0.82494
53959	AA914427	EST AA914427	−1.05313
54125	Polm	polymerase (DNA directed), mu	−0.84766
54354	Rassf5	Ras association (RalGDS/AF-6) domain family member 5	−1.9136
54397	Ppt2	palmitoyl-protein thioesterase 2	0.646458
54646	Ppp1r3f	protein phosphatase 1, regulatory (inhibitor) subunit 3F	−0.70748
54667	Atp8b2	ATPase, class I, type 8B, member 2	−0.68706
54710	Hs3st3b1	heparan sulfate (glucosamine) 3-O-sulfotransferase 3B1	−1.31401
54712	Plxnc1	plexin C1	−1.25562
55983	Pdzrn3	PDZ domain containing RING finger 3	0.683867
55984	Camkk1	calcium/calmodulin-dependent protein kinase kinase 1, alpha	−0.79255
55993	Msh4	mutS homolog 4 (E. coli)	1.045297
55994	Smad9	SMAD family member 9	−0.94001
56041	Uso1	USO1 vesicle docking factor	−0.79266
56070	Tcerg1	transcription elongation regulator 1 (CA150)	−0.84427
56079	Astn2	astrotactin 2	0.795433
56087	Dnah10	dynein, axonemal, heavy chain 10	−1.33786
56149	Grasp	GRP1 (general receptor for phosphoinositides 1)-associated scaffold protein	−0.88589
56191	Tro	trophinin	−0.80328
56193	Plek	pleckstrin	−1.0003
56198	Heyl	hairy/enhancer-of-split related with YRPW motif-like	−0.70871
56273	Pex14	peroxisomal biogenesis factor 14	−0.82539
56305	Pitpnb	phosphatidylinositol transfer protein, beta	−0.86783
56323	Dnajb5	DnaJ (Hsp40) homolog, subfamily B, member 5	0.591571
56380	Arid3b	AT rich interactive domain 3B (BRIGHT-like)	−0.71907
56437	Rrad	Ras-related associated with diabetes	−0.75675
56448	Cyp2d22	cytochrome P450, family 2, subfamily d, polypeptide 22	0.760234
56484	Foxo3	forkhead box O3	0.778968
56692	Lamtor3	late endosomal/lysosomal adaptor, MAPK and MTOR activator 3	−0.63953
56696	Gpr132	G protein-coupled receptor 132	−1.21147
56702	Hist1h1b	histone cluster 1, H1b	1.152689
56708	Clcf1	cardiotrophin-like cytokine factor 1	−0.92025
56743	Lat2	linker for activation of T cells family, member 2	−0.59939
56747	Sez6l	seizure related 6 homolog like	0.703235
56752	Aldh9a1	aldehyde dehydrogenase 9, subfamily A1	0.749505
57257	Vav3	vav 3 oncogene	−0.62284
57316	C1d	C1D nuclear receptor co-repressor	−0.66216
57781	Cd200r1	CD200 receptor 1	−0.75241
57815	Spata5	spermatogenesis associated 5	−0.86262
57915	Tbc1d1	TBC1 domain family, member 1	−0.92906
58180	Hic2	hypermethylated in cancer 2	−0.59036
58198	Sall1	sal-like 1 (Drosophila)	−0.86838
58206	Zbtb32	zinc finger and BTB domain containing 32	−0.64192
58229	Efcc1	EF hand and coiled-coil domain containing 1	0.709159
58239	Dexi	dexamethasone-induced transcript	−0.6686
58242	Nudt11	nudix (nucleoside diphosphate linked moiety X)-type motif 11	−1.38558
58248	1700123O20Rik	RIKEN cDNA 1700123O20 gene	−0.61058
58802	Kcnmb4	potassium large conductance calcium-activated channel, subfamily M, beta member 4	−0.92256
58803	Pga5	pepsinogen 5, group I	0.756267
58804	Cdc42ep5	CDC42 effector protein (Rho GTPase binding) 5	0.79136
58894	Zfp862-ps	zinc finger protein 862, pseudogene	−0.85602
60321	Wbp11	WW domain binding protein 11	−0.66117
60322	Chst7	carbohydrate (N-acetylglucosamino) sulfotransferase 7	−0.78254
60406	Sap30	sin3 associated polypeptide	−0.87835
60531	Npvf	neuropeptide VF precursor	−1.35333
63859	Impg1	interphotoreceptor matrix proteoglycan 1	0.767522
63953	Dusp10	dual specificity phosphatase 10	−0.93348
64085	Clstn2	calsyntenin 2	−1.24802
64095	Gpr35	G protein-coupled receptor 35	0.628931
64214	Rgs18	regulator of G-protein signaling 18	−0.96826
64291	Osbpl1a	oxysterol binding protein-like 1A	0.659995
64383	Sirt2	sirtuin 2	0.588691
64450	Gpr85	G protein-coupled receptor 85	−1.36561
64654	Fgf23	fibroblast growth factor 23	0.67401
65079	Rtn4r	reticulon 4 receptor	−1.74136
65107	Lrp10	low-density lipoprotein receptor-related protein 10	0.681082
66011	Ranbp17	RAN binding protein 17	−1.07455
66086	Fopnl	Fgfr1op N-terminal like	−0.62764
66118	Sarnp	SAP domain containing ribonucleoprotein	−0.71805
66120	Fkbp11	FK506 binding protein 11	−3.83345
66140	Ska2	spindle and kinetochore associated complex subunit 2	−2.08181
66156	Anapc11	anaphase promoting complex subunit 11	0.688481
66158	Cxx1a	CAAX box 1A	0.689986
66185	1110037F02Rik	RIKEN cDNA 1110037F02 gene	−0.93276
66194	Pycrl	pyrroline-5-carboxylate reductase-like	−0.6542
66223	Mrpl35	mitochondrial ribosomal protein L35	−0.89475
66225	Llph	LLP homolog, long-term synaptic facilitation (Aplysia)	−0.65982
66226	Trappc2	trafficking protein particle complex 2	−0.87458
66240	Kcne1l	potassium voltage-gated channel, Isk-related family, member 1-like, pseudogene	−1.58358
66259	Camk2n1	calcium/calmodulin-dependent protein kinase II inhibitor 1	−0.76737
66334	1700025K04Rik	RIKEN cDNA 1700025K04 gene	−2.90572
66410	Mterf3	mitochondrial transcription termination factor 3	−0.90271
66440	Cdc26	cell division cycle 26	−0.79685
66456	2810001G20Rik	RIKEN cDNA 2810001G20 gene	−0.7053
66548	Adamtsl5	ADAMTS-like 5	0.651309
66618	Snrnp27	small nuclear ribonucleoprotein 27 (U4/U6.U5)	−0.70114
66638	5730458M16Rik	RIKEN cDNA 5730458M16 gene	−0.73631
66681	Pgm1	phosphoglucomutase 1	−0.60273
66684	Tceal8	transcription elongation factor A (SII)-like 8	−0.63863
66707	Nkapl	NFKB activating protein-like	−0.93526
66714	4921524J17Rik	RIKEN cDNA 4921524J17 gene	−0.82902
66765	4933411K16Rik	RIKEN cDNA 4933411K16 gene	0.616624
66776	Pisd-ps3	phosphatidylserine decarboxylase, pseudogene 3	0.667682
66789	Alg14	asparagine-linked glycosylation 14	−0.63171
66910	Tmem107	transmembrane protein 107	−0.6006
66932	Rexo1	REX1, RNA exonuclease 1 homolog (S. cerevisiae)	0.714589
66939	Aagab	alpha- and gamma-adaptin binding protein	0.644505
66975	Trappc13	trafficking protein particle complex 13	−0.75532
67037	Pmf1	polyamine-modulated factor 1	0.838946
67039	Rbm25	RNA binding motif protein 25	−0.75041
67050	Nkap	NFKB activating protein	−0.77782
67103	Ptgr1	prostaglandin reductase 1	−0.59049
67128	Ube2g1	ubiquitin-conjugating enzyme E2G 1	−0.6596
67136	Kbtbd4	kelch repeat and BTB (POZ) domain containing 4	−0.61233
67155	Smarca2	SWI/SNF related, matrix associated, actin dependent regulator of chromatin, subfamily a, member 2	0.818912
67164	Lipt2	lipoyl(octanoyl) transferase 2 (putative)	−0.6135
67222	Srfbp1	serum response factor binding protein 1	−0.60051
67228	Dph7	diphthamine biosynethesis 7	−0.64482
67239	Rpf2	ribosome production factor 2 homolog (S. cerevisiae)	−0.83676
67266	Fam69a	family with sequence similarity 69, member A	−0.74891
67311	Nanp	N-acetylneuraminic acid phosphatase	−0.63962
67329	1700018L02Rik	RIKEN cDNA 1700018L02 gene	0.653454
67379	Dedd2	death effector domain-containing DNA binding protein 2	−0.63439
67412	Soga3	SOGA family member 3	−1.0559
67416	Armcx2	armadillo repeat containing, X-linked 2	−0.63628
67490	Ufl1	UFM1 specific ligase 1	−0.60828
67492	Zfand4	zinc finger, AN1-type domain 4	−1.07333
67504	Rnf151	ring finger protein 151	−0.6958
67543	Pabpc6	poly(A) binding protein, cytoplasmic 6	−1.26116
67552	H2afy3	H2A histone family, member Y3	−0.64924
67556	Pigm	phosphatidylinositol glycan anchor biosynthesis, class M	−0.65397
67581	Tbc1d23	TBC1 domain family, member 23	−0.96763
67605	Akt1s1	AKT1 substrate 1 (proline-rich)	0.61903
67618	Aasdhppt	aminoadipate-semialdehyde dehydrogenase-phosphopantetheinyl transferase	−0.83089
67619	Nob1	NIN1/RPN12 binding protein 1 homolog (S. cerevisiae)	−0.68517
67647	4930523C07Rik	RIKEN cDNA 4930523C07 gene	−0.63528
67685	Dyx1c1	dyslexia susceptibility 1 candidate 1 homolog (human)	−0.72161
67708	Pcnxl4	pecanex-like 4 (Drosophila)	−0.60239
67726	Fam114a2	family with sequence similarity 114, member A2	0.697382
67728	Dph2	DPH2 homolog (S. cerevisiae)	−0.59488
67746	4930577N17Rik	RIKEN cDNA 4930577N17 gene	−0.7114
67749	Mgarp	mitochondria localized glutamic acid rich protein	−1.12453
67832	Brix1	BRX1, biogenesis of ribosomes, homolog (S. cerevisiae)	−0.67051
67843	Slc35a4	solute carrier family 35, member A4	0.624622
67883	Uxs1	UDP-glucuronate decarboxylase 1	−0.65658
67902	Sumf2	sulfatase modifying factor 2	−0.78246
67920	Mak16	MAK16 homolog (S. cerevisiae)	−0.69434
67934	1700124L16Rik	RIKEN cDNA 1700124L16 gene	−1.23527
67952	Tomm20	translocase of outer mitochondrial membrane 20 homolog (yeast)	−0.58862
67956	Setd8	SET domain containing (lysine methyltransferase) 8	0.765426
68037	2900093K20Rik	RIKEN cDNA 2900093K20 gene	−0.70538
68040	Zfp593	zinc finger protein 593	−0.65741
68055	Atp5s	ATP synthase, H+ transporting, mitochondrial F0 complex, subunit S	−0.61914
68075	Lurap1	leucine rich adaptor protein 1	−0.73969
68127	B230217C12Rik	RIKEN cDNA B230217C12 gene	−0.97611
68163	A930006D01Rik	RIKEN cDNA A930006D01 gene	−2.03335
68226	Efcab2	EF-hand calcium binding domain 2	0.779822
68299	Vps53	vacuolar protein sorting 53 (yeast)	−0.95112
68304	Kdelc2	KDEL (Lys-Asp-Glu-Leu) containing 2	−0.59902
68318	Aph1c	anterior pharynx defective 1c homolog (C. elegans)	−0.84754
68375	Ndufa8	NADH dehydrogenase (ubiquinone) 1 alpha subcomplex, 8	−0.68243
68402	0710001A04Rik	RIKEN cDNA 0710001A04 gene	−1.0965
68465	Adipor2	adiponectin receptor 2	0.789982
68691	Kansl1l	KAT8 regulatory NSL complex subunit 1-like	−0.63651
68703	Rere	arginine glutamic acid dipeptide (RE) repeats	0.677792
68725	1110032F04Rik	RIKEN cDNA 1110032F04 gene	−2.59569
68750	Rreb1	ras responsive element binding protein 1	−0.6608
68770	Phtf2	putative homeodomain transcription factor 2	−0.75238
68776	Taf11	TAF11 RNA polymerase II, TATA box binding protein (TBP)-associated factor	−0.64046
68938	Aspscr1	alveolar soft part sarcoma chromosome region, candidate 1 (human)	0.878954
68946	1500002C15Rik	RIKEN cDNA 1500002C15 gene	−0.86573
68979	Nol11	nucleolar protein 11	−1.06513
69002	1500026H17Rik	RIKEN cDNA 1500026H17 gene	−0.70915
69202	Ptms	parathymosin	0.770376
69237	Gtpbp4	GTP binding protein 4	−0.66275
69428	1700016C15Rik	RIKEN cDNA 1700016C15 gene	−0.75653
69459	Ubl7	ubiquitin-like 7 (bone marrow stromal cell-derived)	1.154686
69519	Rwdd2a	RWD domain containing 2A	−0.85754
69581	Rhou	ras homolog gene family, member U	−0.67623
69608	Sec24d	Sec24 related gene family, member D (S. cerevisiae)	−0.71508
69627	Fam89a	family with sequence similarity 89, member A	−0.98462
69635	Dapk1	death associated protein kinase 1	−0.74639
69683	Emc10	ER membrane protein complex subunit 10	0.911207
69690	2310057B04Rik	RIKEN cDNA 2310057B04 gene	−0.65452
69749	Epb4.1l4aos	erythrocyte protein band 4.1 like 1 opposite strand	−0.86027
69769	Tnfaip8l2	tumor necrosis factor, alpha-induced protein 8-like 2	−1.08013
69809	1810046K07Rik	RIKEN cDNA 1810046K07 gene	−1.42871
69810	Clec4b1	C-type lectin domain family 4, member b1	−0.84026
69849	2010007H06Rik	RIKEN cDNA 2010007H06 gene	−1.37376
69863	Ttc39b	tetratricopeptide repeat domain 39B	−0.99521
69922	Vrk2	vaccinia related kinase 2	−0.66165
69930	Zfp715	zinc finger protein 715	−0.6557
69976	Galk2	galactokinase 2	−0.64906
69981	Tmem30a	transmembrane protein 30A	−0.77382
69987	1700026L06Rik	RIKEN cDNA 1700026L06 gene	−0.7874
70001	1700028B04Rik	NADH dehydrogenase (ubiquinone) 1 beta subcomplex 8 pseudogene	−0.74908
70057	2210008F06Rik	RIKEN cDNA 2210008F06 gene	−1.03059
70082	Lysmd2	LysM, putative peptidoglycan-binding, domain containing 2	−0.739
70190	2610036A22Rik	RIKEN cDNA 2610036A22 gene	−0.96837
70227	Zfp619	zinc finger protein 619	−0.61798
70296	Tbc1d13	TBC1 domain family, member 13	0.907941
70317	Arl16	ADP-ribosylation factor-like 16	−0.61566
70325	Pigw	phosphatidylinositol glycan anchor biosynthesis, class W	−0.75659
70355	Gprc5c	G protein-coupled receptor, family C, group 5, member C	−0.67835
70426	Tekt5	tektin 5	0.70459
70449	2610209C05Rik	RIKEN cDNA 2610209C05 gene	−1.40834
70458	2610318N02Rik	RIKEN cDNA 2610318N02 gene	−0.92819
70489	5730405O15Rik	RIKEN cDNA 5730405O15 gene	−0.60839
70650	Zcchc8	zinc finger, CCHC domain containing 8	−0.94749
70680	3021401N23Rik	RIKEN cDNA 3021401N23 gene	−0.69732
70691	3830403N18Rik	RIKEN cDNA 3830403N18 gene	−1.62532
70720	6330407A03Rik	RIKEN cDNA 6330407A03 gene	−1.01181
70839	P2ry12	purinergic receptor P2Y, G-protein coupled 12	−0.98975
70904	4921515G04Rik	RIKEN cDNA 4921515G04 gene	−0.6536
70925	Cdkn2aip	CDKN2A interacting protein	−0.93838
70948	Wdr20rt	WD repeat domain 20, retrogene	−1.54168
70998	Phf6	PHD finger protein 6	−0.64555
71063	Zfp597	zinc finger protein 597	−0.86648
71091	Cdkl1	cyclin-dependent kinase-like 1 (CDC2-related kinase)	−0.75399
71123	4933411B09Rik	RIKEN cDNA 4933411B09 gene	−1.38929
71132	Cabyr	calcium-binding tyrosine-(Y)-phosphorylation regulated (fibrousheathin 2)	−1.43923
71133	4933422A05Rik	RIKEN cDNA 4933422A05 gene	−0.95134
71147	Oxsm	3-oxoacyl-ACP synthase, mitochondrial	−0.77899
71176	Fbxo24	F-box protein 24	−1.0756
71398	5430427O19Rik	RIKEN cDNA 5430427O19 gene	−1.02284
71436	Flrt3	fibronectin leucine rich transmembrane protein 3	−0.662
71494	8430406P12Rik	RIKEN cDNA 8430406P12 gene	−1.05142
71524	8430432A02Rik	RIKEN cDNA 8430432A02 gene	−0.7714
71544	Arhgap42	Rho GTPase activating protein 42	−0.65337
71583	9130008F23Rik	RIKEN cDNA 9130008F23 gene	−0.6464
71599	Senp8	SUMO/sentrin specific peptidase 8	−1.02521
71682	Wdr27	WD repeat domain 27	−0.85462
71722	Cic	capicua homolog (Drosophila)	0.612276
71726	Smug1	single-strand selective monofunctional uracil DNA glycosylase	0.842899
71740	Pvrl4	poliovirus receptor-related 4	−1.81837
71770	Ap2b1	adaptor-related protein complex 2, beta 1 subunit	−0.83671
71776	Tha1	threonine aldolase 1	0.590807
71780	Isyna1	myo-inositol 1-phosphate synthase A1	0.66428
71795	Pitpnc1	phosphatidylinositol transfer protein, cytoplasmic 1	−0.95258
71819	Kif23	kinesin family member 23	0.642121
71870	Cfap45	cilia and flagella associated protein 45	−0.94117
71873	2310003N18Rik	RIKEN cDNA 2310003N18 gene	1.017664
71916	Dus4l	dihydrouridine synthase 4-like (S. cerevisiae)	−0.83778
71923	2310047M10Rik	RIKEN cDNA 2310047M10 gene	−0.61514
71934	Car13	carbonic anhydrase 13	−0.70619
71950	Nanog	Nanog homeobox	1.055165
71951	Gpc2	glypican 2 (cerebroglycan)	−0.78001
71971	Zswim1	zinc finger SWIM-type containing 1	−0.76874
71989	Rpusd4	RNA pseudouridylate synthase domain containing 4	−0.61413
71997	Smg9	smg-9 homolog, nonsense mediated mRNA decay factor (C. elegans)	0.880594
72117	Naa50	N(alpha)-acetyltransferase 50, NatE catalytic subunit	−0.63577
72145	Wdfy3	WD repeat and FYVE domain containing 3	0.75203
72167	Thumpd2	THUMP domain containing 2	−0.69783
72171	Shq1	SHQ1 homolog (S. cerevisiae)	−0.92009
72185	Dbndd1	dysbindin (dystrobrevin binding protein 1) domain containing 1	−0.62561
72201	Otud6b	OTU domain containing 6B	−0.60111
72215	1700001P01Rik	RIKEN cDNA 1700001P01 gene	−0.71342
72315	Ccdc74a	coiled-coil domain containing 74A	−0.64998
72333	Palld	palladin, cytoskeletal associated protein	0.598538
72344	Usp36	ubiquitin specific peptidase 36	0.889101
72388	Ripk4	receptor-interacting serine-threonine kinase 4	−0.64809
72397	Rbm12b1	RNA binding motif protein 12 B1	−0.60613
72425	Katnbl1	katanin p80 subunit B like 1	−0.61353
72459	Htatsf1	HIV TAT specific factor 1	0.591036
72475	Ssbp3	single-stranded DNA binding protein 3	0.991036
72515	Wdr43	WD repeat domain 43	−0.60297
72543	Mvb12b	multivesicular body subunit 12B	−0.83561
72555	Shisa9	shisa family member 9	1.597419
72569	Bbs5	Bardet-Biedl syndrome 5 (human)	−0.83584
72640	Mex3a	mex3 homolog A (C. elegans)	−1.39724
72661	Serp2	stress-associated endoplasmic reticulum protein family member 2	−0.60195
72662	Dis3	DIS3 mitotic control homolog (S. cerevisiae)	−0.65819
72716	2810047C21Rik1	RIKEN cDNA 2810047C21 gene 1	−0.65151
72720	Zfp248	zinc finger protein 248	−0.81831
72723	Zfp74	zinc finger protein 74	−0.69623
72750	Fam117b	family with sequence similarity 117, member B	−0.72226
72754	Arhgef10l	Rho guanine nucleotide exchange factor (GEF) 10-like	−0.96016
72789	Veph1	ventricular zone expressed PH domain-containing 1	0.922191
72828	Ubash3b	ubiquitin associated and SH3 domain containing, B	−0.61811
72834	2810468N07Rik	RIKEN cDNA 2810468N07 gene	−0.96156
72893	2900040C04Rik	RIKEN cDNA 2900040C04 gene	0.754736
72902	Spock3	sparc/osteonectin, cwcv and kazal-like domains proteoglycan 3	−1.02503
72925	March1	membrane-associated ring finger (C3HC4) 1	0.801931
72927	Hepacam	hepatocyte cell adhesion molecule	1.042589
72962	Tymp	thymidine phosphorylase	0.615945
73049	2900054C01Rik	RIKEN cDNA 2900054C01 gene	−0.78302
73067	Tmem192	transmembrane protein 192	0.63416
73094	Sgip1	SH3-domain GRB2-like (endophilin) interacting protein 1	−0.99541
73162	Otud3	OTU domain containing 3	−0.69108
73183	5430402O13Rik	RIKEN cDNA 5430402O13 gene	−1.26961
73192	Xpot	exportin, tRNA (nuclear export receptor for tRNAs)	−0.76378
73225	Fam118a	family with sequence similarity 118, member A	−0.66145
73259	Cib4	calcium and integrin binding family member 4	0.724613
73321	1700042O10Rik	RIKEN cDNA 1700042O10 gene	−1.49591
73332	Ccdc30	coiled-coil domain containing 30	0.84663
73341	Arhgef6	Rac/Cdc42 guanine nucleotide exchange factor (GEF) 6	−0.59782
73385	Fam177a	family with sequence similarity 177, member A	−1.47643
73467	1700066M21Rik	RIKEN cDNA 1700066M21 gene	−0.65242
73528	1700081N11Rik	RIKEN cDNA 1700081N11 gene	−0.95721
73634	1700125H20Rik	RIKEN cDNA 1700125H20 gene	−1.57148
73656	Ms4a6c	membrane-spanning 4-domains, subfamily A, member 6C	−0.87714
73689	Bloc1s2	biogenesis of lysosomal organelles complex-1, subunit 2	−0.67351
73730	Lce1l	late cornified envelope 1L	0.597907
73953	4930421J07Rik	RIKEN cDNA 4930421J07 gene	−0.68315
73994	4930449C09Rik	RIKEN cDNA 4930449C09 gene	0.607311
74002	Psd2	pleckstrin and Sec7 domain containing 2	−1.21546
74091	Npl	N-acetylneuraminate pyruvate lyase	−0.87224
74103	Nebl	nebulette	−1.23266
74149	Zfp946	zinc finger protein 946	−0.74177
74197	Gtf2e1	general transcription factor II E, polypeptide 1 (alpha subunit)	−0.65696
74249	Lrrc2	leucine rich repeat containing 2	1.126143
74273	1700064E03Rik	uncharacterized LOC74273	−0.59684
74351	Ddx23	DEAD (Asp-Glu-Ala-Asp) box polypeptide 23	0.985507
74393	Map10	microtubule-associated protein 10	−0.60922
74438	Clvs1	clavesin 1	−1.00791
74448	Arl13a	ADP-ribosylation factor-like 13A	−1.46547
74528	Mgme1	mitochondrial genome maintainance exonuclease 1	−0.85113
74552	Nipal3	NIPA-like domain containing 3	−0.73293
74564	9.13E+15	uncharacterized 9130022E09	−0.92438
74579	4833419E13Rik	RIKEN cDNA 4833419E13 gene	−1.366
74604	4833417J20Rik	RIKEN cDNA 4833417J20 gene	−0.81768
74626	Tmem81	transmembrane protein 81	−0.62761
74635	4930425O10Rik	RIKEN cDNA 4930425O10 gene	−1.00346
74670	Zfp943	zinc finger prtoein 943	−0.59703
74675	Ptchd3	patched domain containing 3	0.674573
74753	5830415F09Rik	RIKEN cDNA 5830415F09 gene	−0.62319
74840	Manf	mesencephalic astrocyte-derived neurotrophic factor	−0.6492
74844	4833447I15Rik	RIKEN cDNA 4833447I15 gene	−0.71396
74963	4930470F04Rik	RIKEN cDNA 4930470F04 gene	−1.71387
75115	4930509E16Rik	RIKEN cDNA 4930509E16 gene	−1.48728
75120	4930509E22Rik	RIKEN cDNA 4930509E22 gene	1.032611
75160	4930543I03Rik	RIKEN cDNA 4930543I03 gene	−1.26272
75203	4930539N22Rik	RIKEN cDNA 4930539N22 gene	−1.98321
75216	Cep128	centrosomal protein 128	0.860122
75224	4930528J11Rik	RIKEN cDNA 4930528J11 gene	0.798136
75357	4930557J02Rik	RIKEN cDNA 4930557J02 gene	1.101407
75516	Ttc32	tetratricopeptide repeat domain 32	−0.58808
75625	Mageh1	melanoma antigen, family H, 1	−0.94946
75627	Snapc1	small nuclear RNA activating complex, polypeptide 1	−0.69748
75645	Ccdc172	coiled-coil domain containing 172	−2.18275
75660	Lin37	lin-37 homolog (C. elegans)	0.595662
75710	Rbm12	RNA binding motif protein 12	−0.71791
75722	4932412D23Rik	RIKEN cDNA 4932412D23 gene	0.739958
75735	Pank1	pantothenate kinase 1	−1.01252
75745	Rian	RNA imprinted and accumulated in nucleus	−0.67685
75754	9030607L02Rik	RIKEN cDNA 9030607L02 gene	−0.63813
75767	Rab11fip1	RAB11 family interacting protein 1 (class I)	−0.59524
75769	Lppr5	lipid phosphate phosphatase-related protein type 5	−1.72522
75812	Tasp1	taspase, threonine aspartase 1	−0.73887
75913	4930579G18Rik	RIKEN cDNA 4930579G18 gene	0.867669
75939	4930579G24Rik	RIKEN cDNA 4930579G24 gene	−0.74769
75974	Dock11	dedicator of cytokinesis 11	−0.60614
75984	5031415H12Rik	RIKEN cDNA 5031415H12 gene	−0.80192
75985	Rab30	RAB30, member RAS oncogene family	−0.92072
75995	5033417F24Rik	RIKEN cDNA 5033417F24 gene	−0.66926
76013	5830407E08Rik	RIKEN cDNA 5830407E08 gene	−1.76901
76056	5830443J22Rik	RIKEN cDNA 5830443J22 gene	−0.71186
76080	Ttpal	tocopherol (alpha) transfer protein-like	−0.7626
76286	1110006E14Rik	RIKEN cDNA 1110006E14 gene	−0.75768
76293	Mfap4	microfibrillar-associated protein 4	0.649903
76311	1110019D14Rik	RIKEN cDNA 1110019D14 gene	−0.59607
76355	Tgds	TDP-glucose 4,6-dehydratase	−1.05112
76371	2810408B13Rik	RIKEN cDNA 2810408B13 gene	−1.45516
76419	Smkr-ps	smal lysine rich protein 1, pseudogene	1.365021
76510	Trappc9	trafficking protein particle complex 9	0.653014
76524	Cln6	ceroid-lipofuscinosis, neuronal 6	0.662862
76561	Snx7	sorting nexin 7	−0.65032
76589	Unc5cl	unc-5 homolog C (C. elegans)-like	−1.04334
76611	1700071A11Rik	RIKEN cDNA 1700071A11 gene	−0.77159
76687	Spcs3	signal peptidase complex subunit 3 homolog (S. cerevisiae)	−0.67412
76794	Man2c1os	mannosidase, alpha, class 2C, member 1, opposite strand	−0.60488
76803	2410141K09Rik	RIKEN cDNA 2410141K09 gene	−0.79015
76820	Fam49a	family with sequence similarity 49, member A	−0.71369
76867	Rhbdd1	rhomboid domain containing 1	−0.71947
76877	Rab36	RAB36, member RAS oncogene family	−1.18065
76918	3110001N23Rik	RIKEN cDNA 3110001N23 gene	−0.74385
76936	Hnrnpm	heterogeneous nuclear ribonucleoprotein M	0.601472
76954	St5	suppression of tumorigenicity 5	1.02554
76971	Sult2a8	sulfotransferase family 2A, dehydroepiandrosterone (DHEA)-preferring, member 8	−0.98833
77045	Bcl7a	B cell CLL/lymphoma 7A	−0.88996
77053	Sun1	Sad1 and UNC84 domain containing 1	0.847962
77056	Tmco4	transmembrane and coiled-coil domains 4	0.738708
77090	Ocel1	occludin/ELL domain containing 1	0.628336
77095	D330022H12Rik	RIKEN cDNA D330022H12 gene	−0.72426
77113	Klhl2	kelch-like 2, Mayven	−0.58965
77209	8030453O22Rik	RIKEN cDNA 8030453O22 gene	−0.63266
77252	9430038I01Rik	RIKEN cDNA 9430038I01 gene	0.745099
77286	Nkrf	NF-kappaB repressing factor	−0.64765
77352	Axdnd1	axonemal dynein light chain domain containing 1	−1.11526
77447	9430096G21Rik	RIKEN cDNA 9430096G21 gene	−1.82291
77519	Zfp266	zinc finger protein 266	−0.66095
77533	C030034I22Rik	RIKEN cDNA C030034I22 gene	−0.80221
77579	Myh10	myosin, heavy polypeptide 10, non-muscle	−0.76395
77599	5830420C07Rik	RIKEN cDNA 5830420C07 gene	−0.74827
77665	9030204H09Rik	RIKEN cDNA 9030204H09 gene	0.599592
77775	A430103D13Rik	RIKEN cDNA A430103D13 gene	−0.80602
77789	A930007D18Rik	RIKEN cDNA A930007D18 gene	−0.87584
77795	A930010G16Rik	RIKEN cDNA A930010G16 gene	−1.07794
77864	Ypel2	yippee-like 2 (Drosophila)	−0.58792
77940	A930004D18Rik	RIKEN cDNA A930004D18 gene	−1.0215
77941	A930001M01Rik	RIKEN cDNA A930001M01 gene	−0.95297
77956	A930026B05Rik	RIKEN cDNA A930026B05 gene	−0.7379
78045	4930564I24Rik	RIKEN cDNA 4930564I24 gene	−0.69919
78109	Lrp8os1	low density lipoprotein receptor-related protein 8, apolipoprotein e receptor, opposite strand 1	0.616691
78174	Cox7b2	cytochrome c oxidase subunit VIIb2	−2.885
78257	Lrrc9	leucine rich repeat containing 9	−0.72909
78316	Platr27	pluripotency associated transcript 27	−1.61941
78353	2500002B13Rik	RIKEN cDNA 2500002B13 gene	−0.60411
78412	3110062M04Rik	RIKEN cDNA 3110062M04 gene	−1.16953
78416	Rnase6	ribonuclease, RNase A family, 6	−1.22221
78428	Wibg	within bgcn homolog (Drosophila)	0.672495
78438	A930028N01Rik	RIKEN cDNA A930028N01 gene	0.62632
78445	C330013E15Rik	RIKEN cDNA C330013E15 gene	−1.96651
78454	9530077C14Rik	RIKEN cDNA 9530077C14 gene	−0.61383
78603	B230216N24Rik	RIKEN cDNA B230216N24 gene	−1.03526
78610	Uvrag	UV radiation resistance associated gene	−0.62355
78619	Zfp449	zinc finger protein 449	−0.72235
78668	E130112N10Rik	RIKEN cDNA E130112N10 gene	−0.68212
78784	Celf3	CUGBP, Elav-like family member 3	−0.63526
78825	Desi2	desumoylating isopeptidase 2	−0.5877
78829	Tsc22d4	TSC22 domain family, member 4	0.627395
78921	9130019O22Rik	RIKEN cDNA 9130019O22 gene	−0.74487
78926	Gas2l1	growth arrest-specific 2 like 1	0.767342
79362	Bhlhe41	basic helix-loop-helix family, member e41	−1.14259
79459	Aldoart2	aldolase 1 A, retrogene 2	1.158981
80509	Med8	mediator complex subunit 8	−0.67295
80707	Wwox	WW domain-containing oxidoreductase	−0.64542
80743	Vps16	vacuolar protein sorting 16 (yeast)	−1.05958
80883	Ntng1	netrin G1	−0.98812
80890	Trim2	tripartite motif-containing 2	−0.92197
80901	Cxcr6	chemokine (C-X-C motif) receptor 6	−0.90825
80904	Dtx3	deltex 3 homolog (Drosophila)	0.659925
81910	Rrbp1	ribosome binding protein 1	0.658042
83561	Tdrd1	tudor domain containing 1	−1.55906
83669	Wdr6	WD repeat domain 6	0.836221
83672	Sytl3	synaptotagmin-like 3	−0.87915
83675	Bicc1	bicaudal C homolog 1 (Drosophila)	−0.59427
83691	Crispld1	cysteine-rich secretory protein LCCL domain containing 1	−1.71477
83885	Slc25a2	solute carrier family 25 (mitochondrial carrier, ornithine transporter) member 2	−0.69483
84094	Plvap	plasmalemma vesicle associated protein	0.79435
84543	Sval2	seminal vesicle antigen-like 2	−1.40835
85030	Tnfrsf25	tumor necrosis factor receptor superfamily, member 25	−0.78213
93688	Klhl1	kelch-like 1	−0.70661
93717	Pcdhga9	protocadherin gamma subfamily A, 9	0.656106
93761	Smarca1	SWI/SNF related, matrix associated, actin dependent regulator of chromatin, subfamily a, member 1	−0.71967
93889	Pcdhb18	protocadherin beta 18	−0.59971
94043	Tm2d1	TM2 domain containing 1	−0.61284
94091	Trim11	tripartite motif-containing 11	−0.70273
94109	Csmd1	CUB and Sushi multiple domains 1	−1.29077
94184	Pdxdc1	pyridoxal-dependent decarboxylase domain containing 1	0.834688
94218	Cnnm3	cyclin M3	0.619263
94221	Gopc	golgi associated PDZ and coiled-coil motif containing	−0.72954
94253	Hecw1	HECT, C2 and WW domain containing E3 ubiquitin protein ligase 1	−1.23232
97086	Slc9b2	solute carrier family 9, subfamily B (NHA2, cation proton antiporter 2), member 2	−0.94933
97122	Hist2h4	histone cluster 2, H4	0.881916
97212	Hadha	hydroxyacyl-Coenzyme A dehydrogenase/3-ketoacyl-Coenzyme A thiolase/enoyl-Coenzyme A hydratase (trifunctional protein), alpha subunit	0.767457
97550	C130081A10Rik	RIKEN cDNA C130081A10 gene	−0.80604
97775	D930048N14Rik	RIKEN cDNA D930048N14 gene	−0.8934
97848	Serpinb6c	serine (or cysteine) peptidase inhibitor, clade B, member 6c	−0.82304
97874	B430203I24Rik	RIKEN cDNA B430203I24 gene	−0.70653
97884	B3galnt2	UDP-GalNAc:betaGlcNAc beta 1,3-galactosaminyltransferase, polypeptide 2	−0.79603
98303	D630023F18Rik	RIKEN cDNA D630023F18 gene	−0.60981
98662	AW061147	expressed sequence AW061147	−1.07209
98878	Ehd4	EH-domain containing 4	−1.42552
99029	AI596198	expressed sequence AI596198	−0.67529
99041	AI646519	expressed sequence AI646519	−0.71972
99683	Sec24b	Sec24 related gene family, member B (S. cerevisiae)	−0.80383
99730	Taf13	TAF13 RNA polymerase II, TATA box binding protein (TBP)-associated factor	−0.68556
99889	Arfip1	ADP-ribosylation factor interacting protein 1	−0.58876
99890	Prmt6	protein arginine N-methyltransferase 6	−0.6627
100061	Lrrc19	leucine rich repeat containing 19	0.862128
100072	Camta1	calmodulin binding transcription activator 1	−0.67293
100088	Rcc1	regulator of chromosome condensation 1	0.761578
100177	Zmym6	zinc finger, MYM-type 6	−0.65018
100317	AU040320	expressed sequence AU040320	0.786443
100465	Mob3c	MOB kinase activator 3C	−0.75174
100515	Zfp518b	zinc finger protein 518B	−0.85298
100535	Oas1d	2'-5' oligoadenylate synthetase 1D	−0.63228
100637	N4bp2l1	NEDD4 binding protein 2-like 1	−0.87386
100710	Pds5b	PDS5, regulator of cohesion maintenance, homolog B (S. cerevisiae)	−0.6391
100740	AI839979	expressed sequence AI839979	−0.79714
100910	Chpf2	chondroitin polymerizing factor 2	−0.73774
101148	B630005N14Rik	RIKEN cDNA B630005N14 gene	−0.62268
101646	B830008H07Rik	RIKEN cDNA B830008H07 gene	−0.60041
101835	AW146154	expressed sequence AW146154	−0.66974
101923	BB212172	expressed sequence BB212172	−0.59223
102436	Lars2	leucyl-tRNA synthetase, mitochondrial	0.602089
102644	Oaf	OAF homolog (Drosophila)	−0.90462
102954	Nudt10	nudix (nucleoside diphosphate linked moiety X)-type motif 10	−1.233
103067	AA522020	expressed sequence AA522020	0.785372
103080	sep-10	septin 10	0.710424
103098	Slc6a15	solute carrier family 6 (neurotransmitter transporter), member 15	−1.42215
103161	Apof	apolipoprotein F	3.593897
103466	Nt5dc3	5'-nucleotidase domain containing 3	−0.75275
103573	Xpo1	exportin 1, CRM1 homolog (yeast)	−0.81124
103817	AI662501	expressed sequence AI662501	−0.75723
103841	Cuedc1	CUE domain containing 1	0.72041
103967	Dnm3	dynamin 3	−0.87888
104307	Rnu12	RNA U12, small nuclear	−0.6048
104360	Isl2	insulin related protein 2 (islet 2)	−1.3989
104362	Meig1	meiosis expressed gene 1	−1.5427
104383	Rcor2	REST corepressor 2	−0.75159
104445	Cdc42ep1	CDC42 effector protein (Rho GTPase binding) 1	−0.87179
105083	Pelo	pelota homolog (Drosophila)	−0.70256
105203	Fam208b	family with sequence similarity 208, member B	−0.87285
105245	Txndc5	thioredoxin domain containing 5	−0.64907
105404	BB123696	expressed sequence BB123696	−1.93535
105504	Exoc5	exocyst complex component 5	−0.59824
105525	AI480461	expressed sequence AI480461	−0.93626
106143	Cggbp1	CGG triplet repeat binding protein 1	−0.64008
106258	AI790442	expressed sequence AI790442	−1.17428
106459	BB163080	expressed sequence BB163080	−1.07188
106529	Tecr	trans-2,3-enoyl-CoA reductase	0.673678
106585	Ankrd12	ankyrin repeat domain 12	−0.74893
107047	Psmg2	proteasome (prosome, macropain) assembly chaperone 2	−1.54224
107377	AW492981	expressed sequence AW492981	0.886629
107515	Lgr4	leucine-rich repeat-containing G protein-coupled receptor 4	0.729123
107528	Magee1	melanoma antigen, family E, 1	−0.63916
107723	Slc12a6	solute carrier family 12, member 6	0.590395
107769	Tm6sf1	transmembrane 6 superfamily member 1	−0.74232
108052	Slc14a1	solute carrier family 14 (urea transporter), member 1	−1.05763
108151	Sema3d	sema domain, immunoglobulin domain (Ig), short basic domain, secreted, (semaphorin) 3D	−1.09708
108654	Fam210a	family with sequence similarity 210, member A	−0.64689
108672	Zdhhc15	zinc finger, DHHC domain containing 15	−0.61212
108978	4930555G01Rik	RIKEN cDNA 4930555G01 gene	−1.6494
108989	Tpr	translocated promoter region, nuclear basket protein	−0.66401
109054	Pfdn4	prefoldin 4	−0.68448
109225	Ms4a7	membrane-spanning 4-domains, subfamily A, member 7	−0.63855
109305	Orai1	ORAI calcium release-activated calcium modulator 1	0.664724
109674	Ampd2	adenosine monophosphate deaminase 2	0.681569
109754	Cyb5r3	cytochrome b5 reductase 3	0.703426
109978	Art4	ADP-ribosyltransferase 4	−1.31941
110593	Prdm2	PR domain containing 2, with ZNF domain	−0.78139
110637	Grik4	glutamate receptor, ionotropic, kainate 4	−1.21534
110794	Cebpe	CCAAT/enhancer binding protein (C/EBP), epsilon	−1.09945
114565	Zbtb21	zinc finger and BTB domain containing 21	−1.2948
114666	Krtap5-5	keratin associated protein 5-5	0.654529
116847	Prelp	proline arginine-rich end leucine-rich repeat	0.678198
116914	Slc19a2	solute carrier family 19 (thiamine transporter), member 2	−0.65563
117600	Srgap1	SLIT-ROBO Rho GTPase activating protein 1	0.635125
121021	Cspg4	chondroitin sulfate proteoglycan 4	−0.93035
140580	Elmo1	engulfment and cell motility 1	−0.99056
140780	Bmp2k	BMP2 inducible kinase	−0.80858
170624	Dep1	diabetic embryopathy 1	−0.64353
170731	Mfn2	mitofusin 2	0.591107
170742	Sertad3	SERTA domain containing 3	−0.62528
170755	Sgk3	serum/glucocorticoid regulated kinase 3	−0.88688
170780	Cd209e	CD209e antigen	−1.70641
170833	Hook2	hook homolog 2 (Drosophila)	0.656767
170935	Grid2ip	glutamate receptor, ionotropic, delta 2 (Grid2) interacting protein 1	0.805369
171273	Vmn1r217	vomeronasal 1 receptor 217	0.749356
171285	Havcr2	hepatitis A virus cellular receptor 2	−1.23854
171469	Gpr37l1	G protein-coupled receptor 37-like 1	1.027729
171486	Cd99l2	CD99 antigen-like 2	−0.63381
171580	Mical1	microtubule associated monooxygenase, calponin and LIM domain containing 1	1.057196
192167	Nlgn1	neuroligin 1	−1.85825
192174	Rwdd4a	RWD domain containing 4A	−0.82438
192194	Btnl10	butyrophilin-like 10	0.830443
192285	Phf21a	PHD finger protein 21A	0.721023
192656	Ripk2	receptor (TNFRSF)-interacting serine-threonine kinase 2	−0.63475
192734	Lrrc75b	leucine rich repeat containing 75B	−0.74641
193452	Zfp184	zinc finger protein 184 (Kruppel-like)	−0.80251
193742	Abhd16a	abhydrolase domain containing 16A	0.633779
195712	4930421P07Rik	RIKEN cDNA 4930421P07 gene	−2.64623
207181	Rbms3	RNA binding motif, single stranded interacting protein	−0.66008
207740	Ubald1	UBA-like domain containing 1	0.617406
207911	Mchr1	melanin-concentrating hormone receptor 1	−0.97182
208171	Tmprss7	transmembrane serine protease 7	−0.83198
208501	1810043H04Rik	RIKEN cDNA 1810043H04 gene	−1.11547
208595	Mterf1b	mitochondrial transcription termination factor 1b	−0.60592
208643	Eif4g1	eukaryotic translation initiation factor 4, gamma 1	0.845838
208820	Triqk	triple QxxK/R motif containing	−0.70964
208836	Fanci	Fanconi anemia, complementation group I	−0.99452
208898	Unc13c	unc-13 homolog C (C. elegans)	−0.72491
209032	Zc3hav1l	zinc finger CCCH-type, antiviral 1-like	−0.84852
209601	Erich3	glutamate rich 3	−1.20752
209707	Lcorl	ligand dependent nuclear receptor corepressor-like	−0.61257
210544	Tbc1d31	TBC1 domain family, member 31	−0.74179
210573	Tmem151b	transmembrane protein 151B	−1.04789
210876	Vmn2r111	vomeronasal 2, receptor 111	−0.82831
211228	Lrrc25	leucine rich repeat containing 25	−0.76391
211389	Suox	sulfite oxidase	−0.83785
211480	Kcnj14	potassium inwardly-rectifying channel, subfamily J, member 14	−0.78113
211896	Depdc7	DEP domain containing 7	−1.05402
211922	Dennd6a	DENN/MADD domain containing 6A	−0.61144
212326	Fam149a	family with sequence similarity 149, member A	−0.64819
212528	Trmt1	tRNA methyltransferase 1	0.663803
213068	Tmem71	transmembrane protein 71	−1.39557
213119	Itga10	integrin, alpha 10	−1.49396
213233	Tapbpl	TAP binding protein-like	0.65139
213498	Arhgef11	Rho guanine nucleotide exchange factor (GEF) 11	0.694707
213499	Fbxo42	F-box protein 42	−0.65575
214106	4933430I17Rik	RIKEN cDNA 4933430I17 gene	−0.90814
214162	Kmt2a	lysine (K)-specific methyltransferase 2A	0.700823
214403	Gm4788	predicted gene 4788	−2.03621
214547	She	src homology 2 domain-containing transforming protein E	−0.58549
214812	Zfp609	zinc finger protein 609	0.597878
215015	Fam20b	family with sequence similarity 20, member B	0.901403
215090	Maneal	mannosidase, endo-alpha-like	−0.83128
215114	Hip1	huntingtin interacting protein 1	−0.81737
215627	Zbtb8b	zinc finger and BTB domain containing 8b	−1.71704
215690	Nav1	neuron navigator 1	−0.82661
215748	Cnksr3	Cnksr family member 3	−0.63952
216197	Ckap4	cytoskeleton-associated protein 4	−0.84932
216439	Agap2	ArfGAP with GTPase domain, ankyrin repeat and PH domain 2	−1.55652
216835	Usp43	ubiquitin specific peptidase 43	1.193657
216869	Arrb2	arrestin, beta 2	−0.61054
217262	Abca9	ATP-binding cassette, sub-family A (ABC1), member 9	−0.61863
217305	Cd300ld	CD300 molecule-like family member d	−1.56884
217340	Rnf157	ring finger protein 157	−1.04908
217344	Rhbdf2	rhomboid 5 homolog 2 (Drosophila)	−0.6141
217356	Tmc8	transmembrane channel-like gene family 8	0.644216
217449	Trappc12	trafficking protein particle complex 12	0.770431
217692	Sipa1l1	signal-induced proliferation-associated 1 like 1	−0.69194
217695	Zfyve1	zinc finger, FYVE domain containing 1	0.778389
217705	Fam161b	family with sequence similarity 161, member B	−0.68851
217826	Kcnk13	potassium channel, subfamily K, member 13	−0.95761
218441	Zfyve16	zinc finger, FYVE domain containing 16	−0.60524
218544	Sgtb	small glutamine-rich tetratricopeptide repeat (TPR)-containing, beta	−0.66448
219131	Phf11a	PHD finger protein 11A	−0.94665
219150	Hmbox1	homeobox containing 1	0.826927
223601	Fam49b	family with sequence similarity 49, member B	−0.58852
223658	Mroh1	maestro heat-like repeat family member 1	0.59568
224023	Klhl22	kelch-like 22	0.741641
224093	Fam43a	family with sequence similarity 43, member A	−1.07559
224273	Crybg3	beta-gamma crystallin domain containing 3	−0.63046
224613	Flywch1	FLYWCH-type zinc finger 1	1.027128
224648	Uhrf1bp1	UHRF1 (ICBP90) binding protein 1	−0.59813
224807	Tmem63b	transmembrane protein 63b	−0.63524
224912	Crb3	crumbs family member 3	−0.68709
225004	BC027072	cDNA sequence BC027072	−0.7081
225160	Thoc1	THO complex 1	−0.68774
225207	Zfp521	zinc finger protein 521	−0.85601
225348	Wdr36	WD repeat domain 36	−0.70459
225845	Pla2g16	phospholipase A2, group XVI	−0.86648
226098	Hectd2	HECT domain containing 2	−0.77252
226351	Tmem185b	transmembrane protein 185B	−0.74692
226562	Prrc2c	proline-rich coiled-coil 2C	−0.61813
226610	Fam78b	family with sequence similarity 78, member B	−0.70465
226652	Arhgap30	Rho GTPase activating protein 30	−1.27679
226695	Ifi205	interferon activated gene 205	−1.09742
226982	Eif5b	eukaryotic translation initiation factor 5B	−1.0119
227058	Dnah7b	dynein, axonemal, heavy chain 7B	−0.61933
227449	Zcchc2	zinc finger, CCHC domain containing 2	−0.60589
228012	Tlk1	tousled-like kinase 1	0.591133
228662	Btbd3	BTB (POZ) domain containing 3	−1.32196
228852	Ppp1r16b	protein phosphatase 1, regulatory (inhibitor) subunit 16B	−1.17843
228880	Zmynd8	zinc finger, MYND-type containing 8	0.596695
229055	Zbtb10	zinc finger and BTB domain containing 10	−0.87408
229504	Isg20l2	interferon stimulated exonuclease gene 20-like 2	−0.72018
229542	Gatad2b	GATA zinc finger domain containing 2B	0.732688
229663	Csde1	cold shock domain containing E1, RNA binding	−1.41927
229675	Rsbn1	rosbin, round spermatid basic protein 1	−0.6836
229694	AI504432	expressed sequence AI504432	−1.37762
229714	Gpr61	G protein-coupled receptor 61	−1.04805
229780	Trmt13	tRNA methyltransferase 13	−1.21023
230085	N28178	expressed sequence N28178	−0.68116
230594	Zcchc11	zinc finger, CCHC domain containing 11	−0.72357
230796	Wdtc1	WD and tetratricopeptide repeats 1	0.647391
230861	Eif4g3	eukaryotic translation initiation factor 4 gamma, 3	0.814682
231151	Tada2b	transcriptional adaptor 2B	−0.587
231287	Atp10d	ATPase, class V, type 10D	−0.99158
231380	Uba6	ubiquitin-like modifier activating enzyme 6	−0.67428
231506	Lin54	lin-54 homolog (C. elegans)	−0.59736
231805	Pilra	paired immunoglobin-like type 2 receptor alpha	−0.96164
231868	E130309D02Rik	RIKEN cDNA E130309D02 gene	−0.63021
231946	Fam221a	family with sequence similarity 221, member A	−0.78758
232164	Paip2b	poly(A) binding protein interacting protein 2B	−0.75229
232341	Wnk1	WNK lysine deficient protein kinase 1	0.863304
232413	Clec12a	C-type lectin domain family 12, member a	−0.84477
232414	Clec9a	C-type lectin domain family 9, member a	−1.05639
232449	Dera	2-deoxyribose-5-phosphate aldolase homolog (C. elegans)	−0.6902
232879	Zbtb45	zinc finger and BTB domain containing 45	0.587431
232946	Bloc1s3	biogenesis of lysosomal organelles complex-1, subunit 3	0.6053
233046	Rasgrp4	RAS guanyl releasing protein 4	−0.65095
233222	Mrgpra3	MAS-related GPR, member A3	−1.66076
233575	Pgap2	post-GPI attachment to proteins 2	0.66794
233670	Olfr6	olfactory receptor 6	1.020888
233752	Insc	inscuteable homolog (Drosophila)	−0.76245
233765	Plekha7	pleckstrin homology domain containing, family A member 7	0.791715
233802	Thumpd1	THUMP domain containing 1	−0.84648
233812	BC030336	cDNA sequence BC030336	−0.65796
233865	D430042O09Rik	RIKEN cDNA D430042O09 gene	0.689748
234479	Gm4890	predicted gene 4890	−0.96266
234595	Slc38a7	solute carrier family 38, member 7	0.684613
234776	Atmin	ATM interactor	−0.59201
234852	Chmp1a	charged multivesicular body protein 1A	0.675486
234889	Gucy1a2	guanylate cyclase 1, soluble, alpha 2	−0.90142
234915	Cep126	centrosomal protein 126	−0.75877
235281	Scn3b	sodium channel, voltage-gated, type III, beta	−1.07314
235300	Tmem136	transmembrane protein 136	−0.97948
235497	Leo1	Leo1, Paf1/RNA polymerase II complex component, homolog (S. cerevisiae)	−1.20671
235504	Slc17a5	solute carrier family 17 (anion/sugar transporter), member 5	0.671279
235599	6430571L13Rik	RIKEN cDNA 6430571L13 gene	−1.40553
235854	Mrgpra4	MAS-related GPR, member A4	0.94099
236082	Dhrsx	dehydrogenase/reductase (SDR family) X chromosome	0.724746
236727	Slc9a7	solute carrier family 9 (sodium/hydrogen exchanger), member 7	−1.61799
237029	4932411N23Rik	RIKEN cDNA 4932411N23 gene	−1.70119
237400	Mex3d	mex3 homolog D (C. elegans)	−0.74985
237433	Gm4925	predicted gene 4925	−0.75051
237859	Ccdc55	coiled-coil domain containing 55	−0.6461
237928	Phospho1	phosphatase, orphan 1	0.959223
238023	Hexdc	hexosaminidase (glycosyl hydrolase family 20, catalytic domain) containing	0.587653
238276	Akap5	A kinase (PRKA) anchor protein 5	−0.73582
238323	Rps6kl1	ribosomal protein S6 kinase-like 1	−0.68172
239157	Pnma2	paraneoplastic antigen MA2	−0.7546
239217	Kctd12	potassium channel tetramerisation domain containing 12	−0.66721
239393	Lrp12	low density lipoprotein-related protein 12	−0.91013
239559	A4galt	alpha 1,4-galactosyltransferase	−0.61236
239796	Mb21d2	Mab-21 domain containing 2	−0.63591
240063	Zfp811	zinc finger protein 811	−0.8079
240120	Zfp119b	zinc finger protein 119b	−0.75377
240215	Slc4a9	solute carrier family 4, sodium bicarbonate cotransporter, member 9	0.724738
240261	Ccdc112	coiled-coil domain containing 112	−0.81534
240427	Setbp1	SET binding protein 1	−0.82105
240614	Ranbp6	RAN binding protein 6	−0.78465
240665	Ccnj	cyclin J	−0.83635
240667	Sec31b	Sec31 homolog B (S. cerevisiae)	0.725795
240753	Plekha6	pleckstrin homology domain containing, family A member 6	0.770152
241062	Pgap1	post-GPI attachment to proteins 1	−0.61956
241230	St8sia6	ST8 alpha-N-acetyl-neuraminide alpha-2,8-sialyltransferase 6	−1.15413
241289	Ppp1r26	protein phosphatase 1, regulatory subunit 26	−0.5953
241520	Fam171b	family with sequence similarity 171, member B	−0.75721
241568	Lrrc4c	leucine rich repeat containing 4C	−0.92716
241638	Lzts3	leucine zipper, putative tumor suppressor family member 3	−0.94913
241688	Dzank1	double zinc ribbon and ankyrin repeat domains 1	−0.64316
241732	Tspyl3	TSPY-like 3	−0.62221
241950	Bbs12	Bardet-Biedl syndrome 12 (human)	−0.63846
242362	Manea	mannosidase, endo-alpha	−0.64504
242384	Lingo2	leucine rich repeat and Ig domain containing 2	−1.35214
242584	Wdr78	WD repeat domain 78	−0.78936
242602	BC055111	cDNA sequence BC055111	−1.08408
242642	Hpdl	4-hydroxyphenylpyruvate dioxygenase-like	−1.15793
243537	Uroc1	urocanase domain containing 1	−1.23138
243574	Kbtbd8	kelch repeat and BTB (POZ) domain containing 8	−1.35829
243621	Iqsec3	IQ motif and Sec7 domain 3	−1.30989
243655	Klre1	killer cell lectin-like receptor family E member 1	−1.48959
243753	2010107G12Rik	RIKEN cDNA 2010107G12 gene	0.675634
243780	E330009J07Rik	RIKEN cDNA E330009J07 gene	−0.61948
243834	Zfp324	zinc finger protein 324	−1.05214
244061	Gm4971	Smad nuclear interacting protein 1 pseudogene	−0.79988
244871	Zc3h12c	zinc finger CCCH type containing 12C	−0.67263
244879	Npat	nuclear protein in the AT region	−0.95615
244891	Scaper	S phase cyclin A-associated protein in the ER	0.61481
244958	Mrap2	melanocortin 2 receptor accessory protein 2	−1.04743
245350	AA414768	expressed sequence AA414768	−0.74258
245572	Tbx22	T-box 22	−2.36944
245622	Fam199x	family with sequence similarity 199, X-linked	−0.72958
245670	Rragb	Ras-related GTP binding B	−0.71673
246102	Rttn	rotatin	−0.74096
246104	Rhbdl3	rhomboid, veinlet-like 3 (Drosophila)	−0.7166
246177	Myo1g	myosin IG	−1.24641
257633	Acsf3	acyl-CoA synthetase family member 3	0.597114
258042	Olfr487	olfactory receptor 487	1.442597
258097	Olfr1500	olfactory receptor 1500	−2.17059
258180	Olfr699	olfactory receptor 699	−1.80076
258269	Olfr930	olfactory receptor 930	0.81376
258272	Olfr1402	olfactory receptor 1402	0.81345
258285	Olfr122	olfactory receptor 122	1.095957
258375	Olfr794	olfactory receptor 794	0.719334
258499	Olfr945	olfactory receptor 945	0.927516
258584	Olfr1101	olfactory receptor 1101	−0.96051
258633	Olfr1153	olfactory receptor 1153	−0.87716
258662	Olfr738	olfactory receptor 738	−0.70938
258810	Olfr665	olfactory receptor 665	−1.43003
258825	Olfr975	olfactory receptor 975	0.863193
258877	Olfr1395	olfactory receptor 1395	0.985627
258992	Olfr1494	olfactory receptor 1494	1.798832
259057	Olfr649	olfactory receptor 649	0.843841
260298	Fev	FEV (ETS oncogene family)	0.587319
263764	Creg2	cellular repressor of E1A-stimulated genes 2	−0.94823
264895	Acsf2	acyl-CoA synthetase family member 2	1.406468
266459	Gm5039	eukaryotic translation initiation factor 1A pseudogene	−5.11673
268294	Zbtb24	zinc finger and BTB domain containing 24	−0.75203
268354	Fam19a2	family with sequence similarity 19, member A2	−1.33155
268470	Ube2z	ubiquitin-conjugating enzyme E2Z (putative)	−0.90113
268564	Zbtb1	zinc finger and BTB domain containing 1	−0.78751
268595	D430019H16Rik	RIKEN cDNA D430019H16 gene	−0.84558
269019	Stk32a	serine/threonine kinase 32A	−0.81441
269023	Zfp608	zinc finger protein 608	0.70976
269033	4930503L19Rik	RIKEN cDNA 4930503L19 gene	−1.10804
269060	Dagla	diacylglycerol lipase, alpha	−1.07842
269113	Nup54	nucleoporin 54	−0.60862
269132	Colgalt2	collagen beta(1-O)galactosyltransferase 2	−0.69347
269378	Ahcy	S-adenosylhomocysteine hydrolase	0.706816
269397	Ss18l1	synovial sarcoma translocation gene on chromosome 18-like 1	−0.65773
269585	Zscan20	zinc finger and SCAN domains 20	−0.84188
269682	Golga3	golgi autoantigen, golgin subfamily a, 3	−0.62261
269713	Clip2	CAP-GLY domain containing linker protein 2	0.717882
269941	Chsy1	chondroitin sulfate synthase 1	−0.67983
270160	Rab39	RAB39, member RAS oncogene family	−1.25899
270163	Myo9a	myosin IXa	0.747452
270190	Ephb1	Eph receptor B1	−0.82718
270624	Spin4	spindlin family, member 4	−0.70933
272350	Gm5065	predicted gene 5065	0.957372
272589	Tbcel	tubulin folding cofactor E-like	0.604728
276891	Timd4	T cell immunoglobulin and mucin domain containing 4	−1.14558
277333	Gm5069	glyceraldehyde-3-phosphate dehydrogenase pseudogene	0.59056
277343	Wfdc8	WAP four-disulfide core domain 8	0.615777
277360	Prex1	phosphatidylinositol-3,4,5-trisphosphate-dependent Rac exchange factor 1	−0.76644
277854	Depdc5	DEP domain containing 5	−0.68308
278240	Spin2c	spindlin family, member 2C	−0.73623
286942	Kif19a	kinesin family member 19A	0.613557
286968	6530437J22Rik	RIKEN cDNA 6530437J22 gene	−1.11696
319148	Hist1h3c	histone cluster 1, H3c	1.163395
319154	Hist2h3b	histone cluster 2, H3b	0.879989
319160	Hist1h4k	histone cluster 1, H4k	0.714299
319166	Hist1h2ae	histone cluster 1, H2ae	−1.25142
319200	Gpr82	G protein-coupled receptor 82	−1.04316
319208	4930403D09Rik	RIKEN cDNA 4930403D09 gene	−2.0435
319272	A130077B15Rik	RIKEN cDNA A130077B15 gene	0.734046
319278	A230050P20Rik	RIKEN cDNA A230050P20 gene	0.766542
319357	C530043A13Rik	RIKEN cDNA C530043A13 gene	1.216349
319460	A130094D17Rik	RIKEN cDNA A130094D17 gene	−0.62704
319463	C230057M02Rik	RIKEN cDNA C230057M02 gene	0.740153
319481	Wdr59	WD repeat domain 59	−0.68606
319504	Nrcam	neuronal cell adhesion molecule	−0.72373
319552	B230216G23Rik	RIKEN cDNA B230216G23 gene	−0.61414
319615	Zfp944	zinc finger protein 944	−0.59127
319670	Eml5	echinoderm microtubule associated protein like 5	−0.72942
319678	D430040D24Rik	RIKEN cDNA D430040D24 gene	−1.015
319688	5930422O12Rik	RIKEN cDNA 5930422O12 gene	−1.16825
319719	Simc1	SUMO-interacting motifs containing 1	−0.7732
319760	D130020L05Rik	RIKEN cDNA D130020L05 gene	−0.60442
319782	A730021G18Rik	RIKEN cDNA A730021G18 gene	−0.68427
319798	A730090N16Rik	RIKEN cDNA A730090N16 gene	1.795917
319818	A930011G23Rik	RIKEN cDNA A930011G23 gene	−1.46833
319839	A530020G20Rik	RIKEN cDNA A530020G20 gene	0.875643
319888	Oacyl	O-acyltransferase like	1.01763
319924	Apba1	amyloid beta (A4) precursor protein binding, family A, member 1	−0.69613
319982	5930430L01Rik	RIKEN cDNA 5930430L01 gene	−0.66615
320057	A630057N01Rik	RIKEN cDNA A630057N01 gene	0.96067
320060	B230308N11Rik	RIKEN cDNA B230308N11 gene	−0.96382
320108	1110019B24Rik	RIKEN cDNA 1110019B24 gene	−1.69477
320148	B430306N03Rik	RIKEN cDNA B430306N03 gene	−1.46054
320214	Maats1	MYCBP-associated, testis expressed 1	−1.11355
320238	A830054O07Rik	RIKEN cDNA A830054O07 gene	−0.67304
320244	Ttll5	tubulin tyrosine ligase-like family, member 5	0.585645
320265	Fam19a1	family with sequence similarity 19, member A1	−0.74359
320279	C630007K24Rik	RIKEN cDNA C630007K24 gene	−0.65845
320383	B230317F23Rik	RIKEN cDNA B230317F23 gene	−0.86395
320384	B230334L07Rik	RIKEN cDNA B230334L07 gene	−0.63226
320387	D930030O05Rik	RIKEN cDNA D930030O05 gene	−0.67588
320404	Itpkb	inositol 1,4,5-trisphosphate 3-kinase B	0.620676
320586	A630089N07Rik	RIKEN cDNA A630089N07 gene	−0.86049
320587	Tmem88b	transmembrane protein 88B	−0.68374
320616	B130006D01Rik	RIKEN cDNA B130006D01 gene	−0.97238
320621	D830044D21Rik	RIKEN cDNA D830044D21 gene	−0.85512
320642	A630066F11Rik	RIKEN cDNA A630066F11 gene	−0.60529
320654	E330024J20Rik	RIKEN cDNA E330024J20 gene	−0.85272
320664	Cass4	Cas scaffolding protein family member 4	−0.60979
320692	9430037G07Rik	RIKEN cDNA 9430037G07 gene	−0.73744
320705	Bend6	BEN domain containing 6	−1.38104
320714	Trappc11	trafficking protein particle complex 11	−0.73732
320770	A630072M18Rik	RIKEN cDNA A630072M18 gene	−1.0799
320790	Chd7	chromodomain helicase DNA binding protein 7	0.67632
320800	9230112E08Rik	RIKEN cDNA 9230112E08 gene	−0.73619
320812	C230071I02Rik	RIKEN cDNA C230071I02 gene	−0.80585
320854	9030203C11Rik	RIKEN cDNA 9030203C11 gene	−0.7223
320896	C330020E22Rik	RIKEN cDNA C330020E22 gene	0.810205
320907	B430105G09Rik	RIKEN cDNA B430105G09 gene	−0.94059
320908	E430014B02Rik	RIKEN cDNA E430014B02 gene	−2.05688
320919	A230107N01Rik	RIKEN cDNA A230107N01 gene	−1.06285
320929	4732460I02Rik	RIKEN cDNA 4732460I02 gene	−0.76996
320951	Pisd	phosphatidylserine decarboxylase	0.612991
320961	A630026N12Rik	RIKEN cDNA A630026N12 gene	−0.69497
321001	C230021G24Rik	RIKEN cDNA C230021G24 gene	−0.85727
321007	Serac1	serine active site containing 1	−0.89922
327749	Gm5079	predicted gene 5079	0.597094
327901	4831410D14	uncharacterized protein 4831410D14	−1.00182
327957	Scimp	SLP adaptor and CSK interacting membrane protein	−1.41472
328019	Spata32	spermatogenesis associated 32	0.623855
328572	Ep300	E1A binding protein p300	−1.15464
328633	4930515I15	uncharacterized protein 4930515I15	−0.96989
328825	Gm5093	predicted gene 5093	−1.61191
328833	Treml2	triggering receptor expressed on myeloid cells-like 2	−1.44539
328977	Zfp532	zinc finger protein 532	−1.09148
329178	Unc80	unc-80 homolog (C. elegans)	−1.00593
329251	Ppp1r12b	protein phosphatase 1, regulatory (inhibitor) subunit 12B	0.615869
329509	1810024B03Rik	RIKEN cDNA 1810024B03 gene	−0.75417
329650	Med12l	mediator complex subunit 12-like	−0.58692
329739	Fam102b	family with sequence similarity 102, member B	−0.75147
329872	Frem1	Fras1 related extracellular matrix protein 1	−0.74056
330301	Zfp786	zinc finger protein 786	−0.96717
330361	Gcfc2	GC-rich sequence DNA binding factor 2	−0.91483
330463	Zfp78	zinc finger protein 78	−0.72744
330662	Dock1	dedicator of cytokinesis 1	0.598972
330836	Slc7a6	solute carrier family 7 (cationic amino acid transporter, y+ system), member 6	−0.85828
331474	Rgag4	retrotransposon gag domain containing 4	−0.88012
331532	Tceal5	transcription elongation factor A (SII)-like 5	−0.85451
332359	Tigd3	tigger transposable element derived 3	−1.07907
333605	Frmpd4	FERM and PDZ domain containing 4	−2.05022
338362	Ust	uronyl-2-sulfotransferase	−1.31467
338364	Trim65	tripartite motif-containing 65	−0.91679
338366	Mia3	melanoma inhibitory activity 3	0.705735
347712	Pramel7	preferentially expressed antigen in melanoma like 7	−1.98677
353237	Pcdhac2	protocadherin alpha subfamily C, 2	−0.70372
368203	Gm5136	predicted gene 5136	−0.87734
380684	Nefh	neurofilament, heavy polypeptide	−0.84363
380839	Serpinb1c	serine (or cysteine) peptidase inhibitor, clade B, member 1c	−1.39057
380842	Stmnd1	stathmin domain containing 1	−0.98107
380855	Rsl1	regulator of sex limited protein 1	−0.74806
380928	Lmo7	LIM domain only 7	0.645333
380977	A330009N23Rik	RIKEN cDNA A330009N23 gene	−0.66633
381022	Kmt2d	lysine (K)-specific methyltransferase 2D	−0.71231
381066	Zfp948	zinc finger protein 948	−0.92967
381280	Hjurp	Holliday junction recognition protein	0.792372
381350	Spag6l	sperm associated antigen 6 like	1.198639
381413	Gpr176	G protein-coupled receptor 176	−1.61273
381418	Ctxn2	cortexin 2	−1.244
381560	Xkr8	X Kell blood group precursor related family member 8 homolog	−0.61954
381633	Gm1673	predicted gene 1673	−0.6134
381792	2310040G24Rik	RIKEN cDNA 2310040G24 gene	−0.64727
381823	Apold1	apolipoprotein L domain containing 1	−0.96821
381921	Taok2	TAO kinase 2	0.631535
381970	Scgb2b2	secretoglobin, family 2B, member 2	−0.85366
381994	E030018B13Rik	RIKEN cDNA E030018B13 gene	−1.638
382111	Susd5	sushi domain containing 5	−0.97382
382275	Gm5168	predicted gene 5168	−1.0048
382301	Sly	Sycp3 like Y-linked	−0.98228
382543	Ankfn1	ankyrin-repeat and fibronectin type III domain containing 1	−2.29511
382620	Tmed8	transmembrane emp24 domain containing 8	−0.65559
384783	Irs2	insulin receptor substrate 2	0.794769
386655	Eid2	EP300 interacting inhibitor of differentiation 2	−0.79541
387514	Tas2r143	taste receptor, type 2, member 143	1.177925
399633	A630014C17Rik	RIKEN cDNA A630014C17 gene	−1.6978
399635	D230044B12Rik	RIKEN cDNA D230044B12 gene	−0.77295
399640	A1300008O04Rik	RIKEN cDNA A1300008O04 gene	−1.00647
402737	A130014A01Rik	RIKEN cDNA A130014A01 gene	−0.72303
402753	D630033A02Rik	RIKEN cDNA D630033A02 gene	−1.14091
402771	C130090I23Rik	RIKEN cDNA C130090I23 gene	−0.79038
407800	Ecm2	extracellular matrix protein 2, female organ and adipocyte specific	−0.74797
407824	BC020402	cDNA sequence BC020402	−0.59659
408058	BC048507	cDNA sequence BC048507	−1.34383
408067	Zfp874b	zinc finger protein 874b	−0.58904
414072	BC031361	cDNA sequence BC031361	−0.80441
432488	Gm17745	predicted gene, 17745	−0.80004
432596	LOC432596	uncharacterized LOC432596	−0.6342
432637	Gm5433	predicted gene 5433	0.8515
432769	Zfp708	zinc finger protein 708	−0.8239
432842	LOC432842	uncharacterized LOC432842	0.876499
433022	Plcxd2	phosphatidylinositol-specific phospholipase C, X domain containing 2	−0.68622
433424	Zeb2os	zinc finger E-box binding homeobox 2, opposite strand	−0.69187
433791	Gm13251	predicted gene 13251	−0.77061
433801	Gm13212	predicted gene 13212	−1.10183
433804	Gm13154	predicted gene 13154	−0.84184
433882	Gm16223	predicted gene 16223	−1.98454
433931	Pigg	phosphatidylinositol glycan anchor biosynthesis, class G	−1.5897
433956	Dnaaf5	dynein, axonemal, assembly factor 5	−0.76322
434156	Eid2b	EP300 interacting inhibitor of differentiation 2B	−0.96003
434172	Gm5592	predicted gene 5592	−1.67145
434198	B130024G19Rik	RIKEN cDNA B130024G19 gene	−0.84788
434232	Iqck	IQ motif containing K	−0.81656
434246	Trim72	tripartite motif-containing 72	0.634362
434249	Gm5602	predicted gene 5602	−0.70145
434377	Zfp560	zinc finger protein 560	−0.95233
434778	Ccdc160	coiled-coil domain containing 160	−1.05991
434797	Gm5640	predicted gene 5640	0.670032
435350	Serpinb6e	serine (or cysteine) peptidase inhibitor, clade B, member 6e	−1.58783
435366	Platr25	pluripotency associated transcript 25	−0.85272
436336	Gm5767	predicted gene 5767	−1.04922
442834	D830031N03Rik	RIKEN cDNA D830031N03 gene	−0.80254
494448	Cbx6	chromobox 6	−0.59216
544848	Gm5784	predicted gene 5784	−1.14614
545007	Gm5796	predicted gene 5796	−0.62013
545056	Gm5801	ubiquitin-conjugating enzyme E2, J2 homolog pseudogene	−0.89454
545389	Cep170	centrosomal protein 170	−0.61291
545471	Zfp345	zinc finger protein 345	−1.6032
545486	Tubb1	tubulin, beta 1 class VI	−0.7624
545554	Ankrd34a	ankyrin repeat domain 34A	−0.98216
545578	Gm5858	predicted gene 5858	−1.53946
546336	Prrg1	proline rich Gla (G-carboxyglutamic acid) 1	−0.76062
547103	Gm10674	predicted gene 10674	−1.3482
547160	Gm14484	predicted gene 14484	−0.59693
548102	LOC548102	uncharacterized LOC548102	−0.76902
548632	BC023719	cDNA sequence BC023719	−1.17744
552875	AU015680	expressed sequence AU015680	−1.26137
619307	9430078G10Rik	RIKEN cDNA 9430078G10 gene	−1.8657
621893	Hist2h2ab	histone cluster 2, H2ab	−1.00183
622208	Gm6297	predicted gene 6297	−0.89178
622320	Kctd21	potassium channel tetramerisation domain containing 21	−1.15251
622976	Gm6377	predicted gene 6377	−1.04066
623169	Gm6402	ribosomal protein S17 pseudogene	−0.70094
623796	Gm12504	prothymosin alpha pseudogene	−2.11846
624367	Gm6498	glyceraldehyde-3-phosphate dehydrogenase pseudogene	0.617721
625210	Gm14625	predicted gene 14625	−0.69256
625421	C230062I16Rik	RIKEN cDNA C230062I16 gene	−0.91903
626305	Scgb1b7	secretoglobin, family 1B, member 7	−2.18039
626316	Gm13051	predicted gene 13051	−1.28385
626359	Wdr93	WD repeat domain 93	−0.95622
626854	Gm38396	predicted gene, 38396	−0.60015
628586	Gm6899	predicted gene 6899	−0.65898
628709	Gm10324	predicted gene 10324	−1.25029
629159	1700008J07Rik	RIKEN cDNA 1700008J07 gene	0.768984
631624	Gm7072	predicted gene 7072	−0.71905
632971	Rergl	RERG/RAS-like	−1.64316
633057	Gm7102	predicted gene 7102	0.701429
633385	Gm7111	predicted gene 7111	−0.68942
635253	Usp51	ubiquitin specific protease 51	−0.91216
639910	Gm20767	predicted gene, 20767	−1.15522
640543	Tgm7	transglutaminase 7	1.020278
654493	8030494B02Rik	Riken cDNA 8030494B02 gene	−0.66953
654498	Hhla1	HERV-H LTR-associating 1	−0.76889
664829	Slx	Sycp3 like X-linked	−0.87022
665155	Srp54b	signal recognition particle 54B	−0.60623
665563	Mthfd2l	methylenetetrahydrofolate dehydrogenase (NADP+ dependent) 2-like	−0.6698
666266	Gm8013	predicted gene 8013	−2.04279
666938	Bend4	BEN domain containing 4	−0.79001
667034	Pnp2	purine-nucleoside phosphorylase 2	−1.24516
667250	Gm12657	predicted gene 12657	−0.71457
667281	H60b	histocompatibility 60b	−1.22405
667705	Gm8773	predicted gene 8773	−0.81139
667766	Gm8801	protein phosphatase 1, regulatory subunit 10 pseudogene	0.595183
668620	Zfp936	zinc finger protein 936	−0.80565
668917	Zfp133-ps	zinc finger protein 133, pseudogene	−1.04998
670496	Gm11564	predicted gene 11564	1.00542
676894	Gm9694	predicted gene 9694	−0.73028
790913	Gm11413	predicted gene 11413	−0.7987
791282	Gm10030	predicted gene 10030	1.757404
791415	Gm12500	predicted gene 12500	−0.70641
100033459	Pydc3	pyrin domain containing 3	−2.01435
100038369	F630201L12Rik	RIKEN cDNA F630201L12 gene	−2.2877
100038410	Gm10536	predicted gene 10536	−1.96018
100038541	Gm10554	predicted gene 10554	−0.90684
100038620	Gm10631	predicted gene 10631	−0.73957
100038641	Gm10452	predicted gene 10452	−0.77865
100038730	Gm10387	predicted gene 10387	−0.76054
100039055	Gm16404	predicted gene 16404	−1.13286
100039065	Gm2030	predicted gene 2030	−1.04397
100039227	Duxbl2	double homeobox B-like 2	−1.47201
100039801	Cldn24	claudin 24	−0.99105
100040044	Gm2568	predicted gene 2568	−1.66025
100040322	3830408C21Rik	RIKEN cDNA 3830408C21 gene	−0.6447
100040416	Gm10071	predicted gene 10071	−1.5075
100040462	Mndal	myeloid nuclear differentiation antigen like	−1.42012
100040724	Mirg	miRNA containing gene	−0.8375
100040834	Gm2990	predicted gene 2990	−1.29896
100041420	Gm3325	predicted gene 3325	−1.00149
100041677	Gm13157	predicted gene 13157	−0.91069
100042498	Mir22hg	Mir22 host gene (non-protein coding)	−0.69247
100042554	Gm3902	predicted gene 3902	−0.69562
100042584	Gm16523	mitochondrial ribosomal protein L40 pseudogene	−0.60524
100042679	Gm16386	zinc finger protein 946 pseudogene	−0.79701
100042922	Gm14483	predicted gene 14483	−0.75136
100042945	Gm4120	predicted gene 4120	0.612395
100043160	Gm10012	cytochrome c oxidase, subunit VIIc pseudogene	0.957175
100043247	Gm4312	predicted gene 4312	−0.97492
100043292	Gm4340	predicted gene 4340	−2.72647
100043431	Gm4430	predicted gene 4430	−0.81773
100043636	AI662270	expressed sequence AI662270	−1.18371
100043836	Scgb2b7	secretoglobin, family 2B, member 7	−0.83891
100044236	Copg2os2	coatomer protein complex, subunit gamma 2, opposite strand 2	−1.70879
100048897	4731419I09Rik	RIKEN cDNA 4731419I09 gene	−1.2436
100049172	Gm10428	predicted gene 10428	−1.42319
100101046	D230018H15Rik	RIKEN cDNA D230018H15 gene	−1.10653
100113398	Adat3	adenosine deaminase, tRNA-specific 3	−0.66756
100126202	9630029G12Rik	RIKEN cDNA 9630029G12 gene	−1.2251
100126243	A030001D20Rik	RIKEN cDNA A030001D20 gene	−0.72168
100126824	Sco2	SCO cytochrome oxidase deficient homolog 2 (yeast)	0.730268
100233208	Gm10778	predicted gene 10778	−1.04693
100303730	Gm16163	predicted gene 16163	0.754236
100303733	Gm10501	predicted gene 10501	−0.61927
100303744	Sprr2a2	small proline-rich protein 2A2	−0.6182
100322896	Dthd1	death domain containing 1	−0.72563
100415902	Gm15658	predicted gene 15658	−1.21315
100415915	A530072M11Rik	RIKEN cDNA gene A530072M11	−0.78401
100502742	1700007L15Rik	RIKEN cDNA 1700007L15 gene	−0.62689
100502764	Gm16617	predicted gene, 16617	0.825367
100502849	Gm19412	predicted gene, 19412	−0.7524
100502936	E330021D16Rik	RIKEN cDNA E330021D16 gene	−0.73087
100502987	Gm17066	predicted gene 17066	−0.71086
100503021	Gm19510	predicted gene, 19510	−1.34725
100503043	Armcx4	armadillo repeat containing, X-linked 4	−0.84865
100503120	A930006K02Rik	RIKEN cDNA A930006K02 gene	−0.72241
100503185	Btbd8	BTB (POZ) domain containing 8	1.038025
100503199	5430416N02Rik	RIKEN cDNA 5430416N02 gene	1.098121
100503388	Gm19668	predicted gene, 19668	0.908692
100503460	Gm19705	predicted gene, 19705	−0.7856
100503463	AI256396	EST AI256396	−1.21866
100503498	Gm16675	predicted gene, 16675	−1.39464
100503823	Gm16973	predicted gene, 16973	−0.77168
100503871	4833432E10Rik	RIKEN cDNA 4833432E10 gene	−1.08814
100504104	Gm16062	predicted gene 16062	−0.76218
100504166	4933421O10Rik	RIKEN cDNA 4933421O10 gene	−0.95235
100504404	H2-Ea-ps	histocompatibility 2, class II antigen E alpha, pseudogene	−1.14313
100504464	E230016K23Rik	RIKEN cDNA E230016K23 gene	−0.67684
100504501	Gm20257	caspase 8 pseudogene	−0.87353
100504591	Gm15987	predicted gene 15987	−1.23069
100856880	Gm9870	predicted gene 9870	−1.47163
100861738	LOC100861738	elongation factor 2 pseudogene	0.619881
100862431	LOC100862431	H-2 class I histocompatibility antigen, D-D alpha chain-like	0.708989
101669761	Gm15694	NADH dehydrogenase (ubiquinone) 1 alpha subcomplex, 7 (B14.5a), pseudogene	−1.02486
102631881	Gm30103	predicted gene, 30103	0.877683
102632087	Gm13373	predicted gene 13373	−1.30443
102632135	Gm30289	predicted gene, 30289	−0.65816
102632597	Gm28523	predicted gene 28523	−0.6761
102632673	Gm15245	predicted gene 15245	0.644342
102632812	Gm30789	predicted gene, 30789	−0.87706
102632821	LOC102632821	60S acidic ribosomal protein P1-like	−0.71021
102632874	Gm30836	predicted gene, 30836	−1.53279
102632891	Gm30848	predicted gene, 30848	−1.38562
102632920	Gm30871	predicted gene, 30871	1.309055
102633823	Gm31555	predicted gene, 31555	−1.93747
102633880	LOC102633880	uncharacterized LOC102633880	−1.36204
102634174	Gm16104	predicted gene 16104	−1.15519
102634215	Gm31854	predicted gene, 31854	−1.40301
102634376	Gm23952	predicted gene, 23952	−1.27918
102634642	Gm32178	predicted gene, 32178	0.99909
102634692	Gm32219	predicted gene, 32219	−1.03775
102634871	Gm32352	predicted gene, 32352	−0.99188
102634922	Gm32391	predicted gene, 32391	0.638654
102635502	LOC102635502	uncharacterized LOC102635502	−1.64675
102635572	Gm38480	predicted gene, 38480	−0.68344
102635880	Gm33104	predicted gene, 33104	−0.7505
102636154	Gm16124	predicted gene 16124	−0.72439
102636349	LOC102636349	uncharacterized LOC102636349	−0.70182
102636446	Gm33509	predicted gene, 33509	−1.64891
102636562	Gm33594	predicted gene, 33594	−1.89551
102636745	Gm33733	predicted gene, 33733	−1.34025
102637088	Gm33990	predicted gene, 33990	−0.68622
102637299	Gm20714	predicted gene 20714	−0.74842
102637409	LOC102637409	uncharacterized LOC102637409	−0.81874
102637572	Gm38499	predicted gene, 38499	0.594527
102637643	Gm34403	predicted gene, 34403	−1.11966
102638002	LOC102638002	uncharacterized LOC102638002	−0.63567
102638420	Gm34991	predicted gene, 34991	0.775052
102638434	Gm16537	predicted gene 16537	−0.97958
102638621	Gm35144	predicted gene, 35144	−0.76214
102638626	Gm35147	predicted gene, 35147	0.605229
102638696	Gm26919	predicted gene, 26919	−1.88055
102638737	Gm35229	predicted gene, 35229	−0.95739
102638849	Gm15764	predicted gene 15764	−0.68516
102639189	Gm35555	predicted gene, 35555	0.888405
102639309	Gm35650	predicted gene, 35650	0.851693
102639375	Gm29514	predicted gene 29514	−0.74413
102639385	LOC102639385	uncharacterized LOC102639385	−1.05062
102639474	Gm35778	predicted gene, 35778	0.936449
102639711	Gm35959	predicted gene, 35959	−0.74889
102639730	Gm35973	predicted gene, 35973	−0.78258
102639982	LOC102639982	uncharacterized LOC102639982	−1.16036
102640359	LOC102640359	uncharacterized LOC102640359	−1.07742
102641119	LOC102641119	uncharacterized LOC102641119	0.676153
102641241	LOC102641241	zinc finger protein 431-like	−0.95231
102641521	LOC102641521	uncharacterized LOC102641521	−1.27549
102641621	LOC102641621	uncharacterized LOC102641621	−1.61991
102642140	LOC102642140	uncharacterized LOC102642140	1.087788
102643134	LOC102643134	uncharacterized LOC102643134	−1.07949
102643243	LOC102643243	uncharacterized LOC102643243	−0.79132
105243072	Gm39102	predicted gene, 39102	0.821623
105243111	LOC105243111	uncharacterized LOC105243111	−0.62293
105243711	LOC105243711	uncharacterized LOC105243711	−1.16616
105244530	LOC105244530	uncharacterized LOC105244530	−0.73536
105244624	Gm12708	predicted gene 12708	−1.36084
105245474	Gm40923	predicted gene, 40923	−2.48661
105246049	Gm41408	predicted gene, 41408	−0.70345

**Table 4:** Differentially expressed genes (DEG) in the colon of mice after 21 days of exposure to E171.

**Entrez gene ID**	**Gene symbol**	**Gene name**	**Log2FC**
11421	Ace	angiotensin I converting enzyme (peptidyl-dipeptidase A) 1	0.651285
12291	Cacna1g	calcium channel, voltage-dependent, T type, alpha 1G subunit	−1.09989
12307	Calb1	calbindin 1	1.282643
12575	Cdkn1a	cyclin-dependent kinase inhibitor 1A (P21)	0.818227
12843	Col1a2	collagen, type I, alpha 2	−0.85437
13367	Diap1	diaphanous homolog 1 (Drosophila)	0.744955
13653	Egr1	early growth response 1	−1.11944
13806	Eno1	enolase 1, alpha non-neuron	0.670466
14048	Eya1	eyes absent 1 homolog (Drosophila)	0.627279
14229	Fkbp5	FK506 binding protein 5	0.888654
14238	Foxf2	forkhead box F2	−0.78798
14464	Gata5	GATA binding protein 5	0.680614
14755	Pigq	phosphatidylinositol glycan anchor biosynthesis, class Q	0.59899
15551	Htr1b	5-hydroxytryptamine (serotonin) receptor 1B	−0.89351
15945	Cxcl10	chemokine (C-X-C motif) ligand 10	−1.61387
16728	L1cam	L1 cell adhesion molecule	0.605456
16835	Ldlr	low density lipoprotein receptor	0.687404
16846	Lep	leptin	0.653292
17116	Mab21l1	mab-21-like 1 (C. elegans)	−1.48895
17254	Slc3a2	solute carrier family 3 (activators of dibasic and neutral amino acid transport), member 2	0.747147
17472	Gbp4	guanylate binding protein 4	−0.96939
17524	Mpp1	membrane protein, palmitoylated	0.637775
18227	Nr4a2	nuclear receptor subfamily 4, group A, member 2	−0.82788
18345	Olfr46	olfactory receptor 46	1.302175
18439	P2rx7	purinergic receptor P2X, ligand-gated ion channel, 7	−0.77769
19326	Rab11b	RAB11B, member RAS oncogene family	0.599463
19387	Rangap1	RAN GTPase activating protein 1	0.658889
20301	Ccl27a	chemokine (C-C motif) ligand 27A	−0.59555
20430	Cyfip1	cytoplasmic FMR1 interacting protein 1	0.838053
20678	Sox5	SRY (sex determining region Y)-box 5	−1.04235
20779	Src	Rous sarcoma oncogene	0.585707
20917	Suclg2	succinate-Coenzyme A ligase, GDP-forming, beta subunit	0.662674
21379	Tbrg4	transforming growth factor beta regulated gene 4	0.748918
21824	Thbd	thrombomodulin	−0.64834
22123	Psmd3	proteasome (prosome, macropain) 26S subunit, non-ATPase, 3	0.638915
22228	Ucp2	uncoupling protein 2 (mitochondrial, proton carrier)	0.595733
22229	Ucp3	uncoupling protein 3 (mitochondrial, proton carrier)	0.598441
22750	Zfp9	zinc finger protein 9	−0.64348
26372	Clcn6	chloride channel 6	−0.58875
27386	Npas3	neuronal PAS domain protein 3	0.834607
30874	C77488	EST C77488	0.835609
50768	Dlc1	deleted in liver cancer 1	−0.588
52234	D7Ertd183e	DNA segment, Chr 7, ERATO Doi 183, expressed	0.640063
52679	E2f7	E2F transcription factor 7	0.638337
54396	Irgm2	immunity-related GTPase family M member 2	−1.0629
55981	Pigb	phosphatidylinositol glycan anchor biosynthesis, class B	0.662836
56492	Cldn18	claudin 18	0.770857
57269	Olfr1507	olfactory receptor 1507	0.696378
57385	P2ry4	pyrimidinergic receptor P2Y, G-protein coupled, 4	1.273995
60406	Sap30	sin3 associated polypeptide	−0.6009
60530	Fignl1	fidgetin-like 1	−0.64374
63859	Impg1	interphotoreceptor matrix proteoglycan 1	0.813132
63953	Dusp10	dual specificity phosphatase 10	−0.76496
64008	Aqp9	aquaporin 9	−0.97315
64705	Dpys	dihydropyrimidinase	−1.84623
66547	2010203P06Rik	RIKEN cDNA 2010203P06 gene	−0.9194
66610	Abi3	ABI gene family, member 3	0.698279
66709	4921507G05Rik	RIKEN cDNA 4921507G05 gene	0.634214
66829	Lrrc75aos2	leucine rich repeat containing 75A, opposite strand 2	1.102741
66933	1700025L06Rik	RIKEN cDNA 1700025L06 gene	0.692497
67266	Fam69a	family with sequence similarity 69, member A	−0.63671
67637	4930470P17Rik	RIKEN cDNA 4930470P17 gene	−1.59037
67690	Prss37	protease, serine 37	0.902707
68201	Ccdc34	coiled-coil domain containing 34	−0.59813
68228	1700095K22Rik	RIKEN cDNA 1700095K22 gene	−0.87726
69065	Chac1	ChaC, cation transport regulator 1	−1.55648
69080	Gmppa	GDP-mannose pyrophosphorylase A	0.595097
69306	Efcab9	EF-hand calcium binding domain 9	−0.75115
69473	Krtap3-1	keratin associated protein 3-1	−2.66099
69748	Aldh16a1	aldehyde dehydrogenase 16 family, member A1	0.586275
69909	2610027K06Rik	RIKEN cDNA 2610027K06 gene	−0.73013
70230	3300002P13Rik	RIKEN cDNA 3300002P13 gene	0.701897
70417	Megf10	multiple EGF-like-domains 10	−0.71301
70831	Krtap31-1	keratin associated protein 31-1	0.675628
70847	4733401D01Rik	RIKEN cDNA 4733401D01 gene	1.290688
70960	4921531P14Rik	RIKEN cDNA 4921531P14 gene	0.7037
71021	4933403J19Rik	RIKEN cDNA 4933403J19 gene	−1.21723
71386	Krtap28-13	keratin associated protein 28-13	−1.3675
71854	Dpep3	dipeptidase 3	−2.03434
71950	Nanog	Nanog homeobox	0.968158
71995	Erv3	endogenous retroviral sequence 3	0.625712
72893	2900040C04Rik	RIKEN cDNA 2900040C04 gene	0.870901
72901	2900011F02Rik	RIKEN cDNA 2900011F02 gene	0.599728
73062	Ppp1r16a	protein phosphatase 1, regulatory (inhibitor) subunit 16A	0.65124
73299	1700041G16Rik	RIKEN cDNA 1700041G16 gene	−0.95323
73336	Prss44	protease, serine 44	0.800819
73432	1700061N14Rik	RIKEN cDNA 1700061N14 gene	0.731943
73456	Izumo1	izumo sperm-egg fusion 1	3.443263
73696	Platr9	pluripotency associated transcript 9	−0.79652
74488	Lrrc15	leucine rich repeat containing 15	−3.15789
74646	Spsb1	splA/ryanodine receptor domain and SOCS box containing 1	0.662786
74970	4930483O08Rik	RIKEN cDNA 4930483O08 gene	0.976605
75085	4930519L02Rik	RIKEN cDNA 4930519L02 gene	0.629165
75120	4930509E22Rik	RIKEN cDNA 4930509E22 gene	0.9049
75288	Slc35f4	solute carrier family 35, member F4	0.588767
77038	Arfgap2	ADP-ribosylation factor GTPase activating protein 2	0.650269
77056	Tmco4	transmembrane and coiled-coil domains 4	0.649722
77397	9530003J23Rik	RIKEN cDNA 9530003J23 gene	1.085436
77940	A930004D18Rik	RIKEN cDNA A930004D18 gene	−0.91426
78082	9230117E06Rik	RIKEN cDNA 9230117E06 gene	−0.84715
78279	5330421C15Rik	RIKEN cDNA 5330421C15 gene	−0.94753
78827	5830426C09Rik	RIKEN cDNA 5830426C09 gene	0.699759
78926	Gas2l1	growth arrest-specific 2 like 1	0.892931
81897	Tlr9	toll-like receptor 9	0.614653
83454	Nxf2	nuclear RNA export factor 2	−0.92136
94184	Pdxdc1	pyridoxal-dependent decarboxylase domain containing 1	0.630685
97402	C86187	expressed sequence C86187	−0.93102
100061	Lrrc19	leucine rich repeat containing 19	0.822892
100155	AI481877	expressed sequence AI481877	0.586151
102975	AU015621	expressed sequence AU015621	1.129115
103203	AI413759	expressed sequence AI413759	−1.11418
103266	Tmem263	transmembrane protein 263	−1.18364
105596	AU020094	expressed sequence AU020094	0.622001
107515	Lgr4	leucine-rich repeat-containing G protein-coupled receptor 4	0.644103
107971	Frs3	fibroblast growth factor receptor substrate 3	0.690578
171190	Vmn1r26	vomeronasal 1 receptor 26	−1.51356
171195	Vmn1r30	vomeronasal 1 receptor 30	0.604116
193796	Kdm4b	lysine (K)-specific demethylase 4B	0.592358
194655	Klf11	Kruppel-like factor 11	−1.01785
208836	Fanci	Fanconi anemia, complementation group I	0.778423
212528	Trmt1	tRNA methyltransferase 1	0.78916
213248	Wdr49	WD repeat domain 49	2.155048
213945	Col28a1	collagen, type XXVIII, alpha 1	−0.65825
214547	She	src homology 2 domain-containing transforming protein E	−0.63251
223658	Mroh1	maestro heat-like repeat family member 1	0.749083
224613	Flywch1	FLYWCH-type zinc finger 1	0.597535
228770	Rspo4	R-spondin family, member 4	0.743283
228775	Trib3	tribbles homolog 3 (Drosophila)	−0.8798
232813	Shisa7	shisa family member 7	1.21652
233549	Mogat2	monoacylglycerol O-acyltransferase 2	−1.13785
233865	D430042O09Rik	RIKEN cDNA D430042O09 gene	0.619914
235674	Acaa1b	acetyl-Coenzyme A acyltransferase 1B	0.695209
237625	Pla2g3	phospholipase A2, group III	−1.41112
241656	Pak7	p21 protein (Cdc42/Rac)-activated kinase 7	0.733928
241764	L3mbtl1	l(3)mbt-like (Drosophila)	−0.67467
242915	Gareml	GRB2 associated, regulator of MAPK1-like	−0.99775
246779	Il27	interleukin 27	0.620833
258064	Olfr316	olfactory receptor 316	0.697396
258199	Olfr1300-ps1	olfactory receptor 1300, pseudogene 1	1.45359
258238	Olfr417	olfactory receptor 417	−0.99523
258267	Olfr370	olfactory receptor 370	−1.87677
258269	Olfr930	olfactory receptor 930	0.853172
258358	Olfr557	olfactory receptor 557	2.218149
258543	Olfr810	olfactory receptor 810	−1.7235
258701	Olfr401	olfactory receptor 401	1.156804
258882	Olfr874	olfactory receptor 874	−1.30081
259114	Olfr570	olfactory receptor 570	1.00058
259145	Olfr1251	olfactory receptor 1251	0.777263
266614	Ly6g5b	lymphocyte antigen 6 complex, locus G5B	0.614901
268510	Mgat5b	mannoside acetylglucosaminyltransferase 5, isoenzyme B	0.931575
269604	Gpr157	G protein-coupled receptor 157	0.984748
270150	Ccdc153	coiled-coil domain containing 153	0.606825
319634	Efcab5	EF-hand calcium binding domain 5	−0.81654
319720	9630028I04Rik	RIKEN cDNA 9630028I04 gene	0.712617
319805	C130073E24Rik	RIKEN cDNA C130073E24 gene	−1.44124
320178	4921529L05Rik	RIKEN cDNA 4921529L05 gene	1.076061
320309	1520401A03Rik	RIKEN cDNA 1520401A03 gene	0.809069
320471	D230022J07Rik	RIKEN cDNA D230022J07 gene	0.797252
320616	B130006D01Rik	RIKEN cDNA B130006D01 gene	−0.6411
320869	Spata33	spermatogenesis associated 33	−1.03489
329253	Gm15850	predicted gene 15850	1.413351
329941	Col8a2	collagen, type VIII, alpha 2	−0.84984
330552	Gm9801	predicted gene 9801	0.596646
333669	Gm5134	predicted gene 5134	1.732013
382083	Snx22	sorting nexin 22	0.621914
382233	Gm5166	DMRT-like family pseudogene	1.08326
382543	Ankfn1	ankyrin-repeat and fibronectin type III domain containing 1	1.216016
384639	Gm5334	tetraspanin 7 pseudogene	0.914592
387345	Tas2r113	taste receptor, type 2, member 113	1.357752
414121	E430016F16Rik	RIKEN cDNA E430016F16 gene	0.960884
432641	Gm17746	predicted gene, 17746	−1.03574
432649	Gm5434	ubiquitin-conjugating enzyme E2F (putative) pseudogene	0.676471
433111	F630040K05Rik	RIKEN cDNA F630040K05 gene	0.590534
433208	D030046N08Rik	RIKEN cDNA D030046N08 gene	−2.4382
433424	Zeb2os	zinc finger E-box binding homeobox 2, opposite strand	−0.64512
434285	BB014433	expressed sequence BB014433	0.599378
434758	Rhox3h	reproductive homeobox 3H	0.738632
435286	Krtap9-5	keratin associated protein 9-5	−0.87046
442803	A830005F24Rik	RIKEN cDNA A830005F24 gene	−1.54044
442816	F830005K03Rik	RIKEN cDNA F830005K03 gene	1.366128
544763	Hbq1b	hemoglobin, theta 1B	−0.7882
624153	H2afb2	H2A histone family, member B2	−0.95194
627367	Vmn2r97	vomeronasal 2, receptor 97	0.650603
629756	Wfdc10	WAP four-disulfide core domain 10	0.719878
632971	Rergl	RERG/RAS-like	−1.22717
634104	Olfr287	olfactory receptor 287	0.724416
665350	Gm7596	predicted gene 7596	−0.82164
675749	Gm10693	predicted pseudogene 10693	−0.73137
1E+08	Gm13274	predicted gene 13274	1.034187
1E+08	Gm10461	predicted gene 10461	0.601642
1E+08	BC048594	cDNA sequence BC048594	0.700788
1E+08	Gm10325	predicted gene 10325	1.05113
1E+08	Gm38398	predicted gene 38398	−0.83597
1E+08	Gm2138	predicted gene 2138	0.627063
1E+08	Tgtp2	T cell specific GTPase 2	−1.42466
1E+08	Gm3364	predicted gene 3364	−0.90308
1E+08	Gm3893	predicted gene 3893	−1.19406
1E+08	Gm4146	predicted gene 4146	0.644792
1E+08	Gm4402	predicted gene 4402	0.781832
1E+08	Gm10584	predicted gene 10584	−0.69025
1E+08	Gm10252	predicted gene 10252	−1.63411
1E+08	Gm11747	predicted gene 11747	0.86802
1.01E+08	A730020M07Rik	RIKEN cDNA A730020M07 gene	0.869668
1.01E+08	Gm12279	predicted gene 12279	−1.10091
1.01E+08	Gm19665	predicted gene, 19665	1.157742
1.01E+08	Gm16754	predicted gene, 16754	−1.13793
1.01E+08	Gm19937	predicted gene, 19937	−0.8815
1.01E+08	Krtap16-1	keratin associated protein 16-1	−0.75551
1.01E+08	Gm20257	caspase 8 pseudogene	−0.59324
1.01E+08	Gm11454	predicted gene 11454	0.626209
1.01E+08	Gm14569	predicted gene 14569	−0.75401
1.03E+08	Gm14642	predicted gene 14642	0.908442
1.03E+08	4930512M02Rik	RIKEN cDNA 4930512M02 gene	0.942116
1.03E+08	Gm13373	predicted gene 13373	−0.7068
1.03E+08	Gm26564	predicted gene, 26564	1.535484
1.03E+08	Gm31805	predicted gene, 31805	0.765069
1.03E+08	Gm31854	predicted gene, 31854	−1.09139
1.03E+08	Gm32296	predicted gene, 32296	0.789333
1.03E+08	LOC102634852	uncharacterized LOC102634852	−1.03989
1.03E+08	Gm32391	predicted gene, 32391	0.722814
1.03E+08	Gm32575	predicted gene, 32575	1.215592
1.03E+08	Gm33452	predicted gene, 33452	−0.85761
1.03E+08	LOC102636367	uncharacterized LOC102636367	0.813995
1.03E+08	Gm38488	predicted gene, 38488	0.814931
1.03E+08	Gm28209	predicted gene 28209	0.681508
1.03E+08	Gm34336	predicted gene, 34336	−2.90205
1.03E+08	Gm15569	predicted gene 15569	0.723968
1.05E+08	Gm38999	predicted gene, 38999	0.641604
1.05E+08	Gm39216	predicted gene, 39216	0.628011

**Table 5:** Group of pathways, pathways, genes related to this pathways and log2FC values after 2 days of exposure to E171 in BALB/c mice. Numbers in bold are upregulated genes. Log2FC=Log2 fold change obtained with LIMMA script with correction for its own time-matched control.

**Group of pathways**	**Pathways**	**EntrezGeneID**	**Genes**	**Log2FC**
Signalling	Odorant GPCRs	258926	Olfr476	−1.66123
		14764	Ptgdr2	−1.13784
		113849	Vmn1r52	−1.06252
		171469	Gpr37l1	−1.00965
		233230	Mrgprb4	−0.99996
		**237716**	**Gpr75**	**0.60654**
		**269604**	**Gpr157**	**0.910932**
		**56544**	**Vmn2r1**	**1.00315**
	Olfactory transduction	258069	Olfr787	−0.88202
		258224	Olfr1358	−0.84454
		258285	Olfr122	−1.24794
		258290	Olfr1143	−0.7162
		383243	Olfr128	−0.81569
		258366	Olfr434	−0.7381
		258375	Olfr794	−0.85019
		258507	Olfr96	−0.87258
		258589	Olfr703	−0.66525
		258590	Olfr702	−1.59164
		258677	Olfr76	−0.91511
		258716	Olfr424	−1.59652
		258750	Olfr551	−0.60955
		258825	Olfr975	−0.91639
		258877	Olfr1395	−0.93299
		259006	Olfr399	−1.18
		259021	Olfr1054	−0.93971
		117005	Olfr74	−1.04523
		257888	Olfr1386	−1.78702
		18331	Olfr32	−0.96808
		57271	Olfr1509	−0.71953
	Olfactory Signalling Pathway	257888	Olfr1386	−1.78702
		258716	Olfr424	−1.59652
		117005	Olfr74	−1.04523
		18331	Olfr32	−0.96808
		259021	Olfr1054	−0.93971
		258677	Olfr76	−0.91511
		258507	Olfr96	−0.87258
		258366	Olfr434	−0.7381
		57271	Olfr1509	−0.71953
		258290	Olfr1143	−0.7162
		258589	Olfr703	−0.66525
	Signalling by GPCR	257888	Olfr1386	−1.78702
		387285	Hcrtr2	−1.70246
		258716	Olfr424	−1.59652
		14764	Ptgdr2	−1.13784
		13492	Drd5	−1.0558
		14602	Ghrhr	−1.05079
		117005	Olfr74	−1.04523
		18331	Olfr32	−0.96808
		259021	Olfr1054	−0.93971
		258677	Olfr76	−0.91511
		258507	Olfr96	−0.87258
		14676	Gna15	−0.86407
		258366	Olfr434	−0.7381
		57271	Olfr1509	−0.71953
		258290	Olfr1143	−0.7162
		258589	Olfr703	−0.66525
		**19206**	**Ptch1**	**0.672454**
		**20302**	**Ccl3**	**2.147125**
	GPCR downstream signalling	257888	Olfr1386	−1.78702
		387285	Hcrtr2	−1.70246
		258716	Olfr424	−1.59652
		14764	Ptgdr2	−1.13784
		13492	Drd5	−1.0558
		14602	Ghrhr	−1.05079
		117005	Olfr74	−1.04523
		18331	Olfr32	−0.96808
		259021	Olfr1054	−0.93971
		258677	Olfr76	−0.91511
		258507	Olfr96	−0.87258
		14676	Gna15	−0.86407
		258366	Olfr434	−0.7381
		57271	Olfr1509	−0.71953
		258290	Olfr1143	−0.7162
		258589	Olfr703	−0.66525
	Signal transduction	257888	Olfr1386	−1.78702
		387285	Hcrtr2	−1.70246
		258716	Olfr424	−1.59652
		14764	Ptgdr2	−1.13784
		12064	Bdnf	−1.07104
		13492	Drd5	−1.0558
		14602	Ghrhr	−1.05079
		117005	Olfr74	−1.04523
		14599	Gh	−1.02848
		18331	Olfr32	−0.96808
		259021	Olfr1054	−0.93971
		258677	Olfr76	−0.91511
		258507	Olfr96	−0.87258
		14676	Gna15	−0.86407
		258366	Olfr434	−0.7381
		57271	Olfr1509	−0.71953
		258290	Olfr1143	−0.7162
		258589	Olfr703	−0.66525
		67603	Dusp6	−0.65975
		**16150**	**Ikbkb**	**0.601827**
		**27412**	**Peg12**	**0.62484**
		**19206**	**Ptch1**	**0.672454**
		**12575**	**Cdkn1a**	**0.728624**
		**20302**	**Ccl3**	**2.147125**
Immune response	Epstein-Barr virus infection	14997	H2-M9	−2.12823
		15511	Hspa1b	−2.10535
		193740	Hspa1a	−1.78004
		69241	Polr2d	−0.66069
		**16150**	**Ikbkb**	**0.601827**
		**12575**	**Cdkn1a**	**0.728624**
		**224754**	**H2-M11**	**1.200937**
	Antigen processing and presentation	14997	H2-M9	−2.12823
		15511	Hspa1b	−2.10535
		193740	Hspa1a	−1.78004
		**224754**	**H2-M11**	**1.200937**
Cancer signalling	MAPK signalling pathway	15511	Hspa1b	−2.10535
		193740	Hspa1a	−1.78004
		12064	Bdnf	−1.07104
		286940	Flnb	−0.81259
		67603	Dusp6	−0.65975
		**16150**	**Ikbkb**	**0.601827**
		**17873**	**Gadd45b**	**0.779545**
		**54652**	**Cacna1f**	**0.882943**
	Protein processing in endoplasmic reticulum	15511	Hspa1b	−2.10535
		193740	Hspa1a	−1.78004
		15505	Hsph1	−1.21534
		15502	Dnaja1	−0.89922
		81489	Dnajb1	−0.67696
		22213	Ube2g2	−0.6056
	Destabilization of mRNA by AUF1 (hnRNP D0)	193740	Hspa1a	−1.78004
		15511	Hspa1b	−2.10535

**Table 6:** Group of pathways, pathways, genes related to this pathways and log2FC values after 7 days of exposure to E171 in BALB/c mice. Numbers in bold are upregulated genes. Log2FC=Log2 fold change obtained with LIMMA script with correction for its own time-matched control.

**Group of pathways**	**Pathways**	**EntrezGeneID**	**Genes**	**Log2FC**
Signalling	Olfactory transduction	259082	Olfr1062	−3.1017
		258390	Olfr1276	−2.28313
		258674	Olfr1427	−2.1351
		18359	Olfr59	−2.08316
		258025	Olfr1211	−1.97936
		18365	Olfr65	−1.93793
		258326	Olfr642	−1.88474
		258409	Olfr1431	−1.85304
		259071	Olfr166	−1.67436
		257912	Olfr948	−1.66079
		258880	Olfr218	−1.65817
		258371	Olfr368	−1.53827
		258177	Olfr1222	−1.53601
		258784	Olfr1245	−1.52883
		258976	Olfr1262	−1.46816
		259023	Olfr1055	−1.46147
		18315	Olfr18	−1.44533
		258616	Olfr357	−1.44533
		258807	Olfr910	−1.42112
		258560	Olfr843	−1.4106
		258800	Olfr905	−1.40489
		258023	Olfr1306	−1.3753
		258494	Olfr318	−1.36443
		258207	Olfr452	−1.35668
		258730	Olfr483	−1.24945
		404222	Olfr231	−1.24083
		258417	Olfr470	−1.23992
		258939	Olfr63	−1.23907
		258662	Olfr738	−1.18753
		258830	Olfr103	−1.16458
		258808	Olfr620	−1.15739
		259041	Olfr1414	−1.12738
		258334	Olfr1396	−1.12314
		257902	Olfr704	−1.09448
		404318	Olfr681	−1.02977
		18314	Olfr17	−0.91665
		258701	Olfr401	−0.88657
		258353	Olfr521	−0.88221
		258359	Olfr1312	−0.85416
		258290	Olfr1143	−0.84815
		258465	Olfr1387	−0.84763
		258310	Olfr145	−0.83906
		258335	Olfr374	−0.82561
		404311	Olfr209	−0.8033
		259105	Olfr549	−0.79583
		107477	Guca1b	−0.72256
		546896	Olfr455	−0.65756
		**259103**	**Olfr616**	**0.595395**
		**258477**	**Olfr197**	**0.727059**
		**233670**	**Olfr6**	**0.93882**
		**258492**	**Olfr484**	**1.013283**
		**258879**	**Olfr330**	**1.320387**
		**258590**	**Olfr702**	**1.771986**
	Olfactory Signalling Pathway	259082	Olfr1062	−3.1017
		258674	Olfr1427	−2.1351
		258025	Olfr1211	−1.97936
		18365	Olfr65	−1.93793
		258409	Olfr1431	−1.85304
		259071	Olfr166	−1.67436
		258880	Olfr218	−1.65817
		258371	Olfr368	−1.53827
		258177	Olfr1222	−1.53601
		258784	Olfr1245	−1.52883
		18315	Olfr18	−1.44533
		258616	Olfr357	−1.44533
		258560	Olfr843	−1.4106
		258494	Olfr318	−1.36443
		258207	Olfr452	−1.35668
		258730	Olfr483	−1.24945
		404222	Olfr231	−1.24083
		258417	Olfr470	−1.23992
		258939	Olfr63	−1.23907
		258830	Olfr103	−1.16458
		258808	Olfr620	−1.15739
		259041	Olfr1414	−1.12738
		257902	Olfr704	−1.09448
		404318	Olfr681	−1.02977
		18314	Olfr17	−0.91665
		258701	Olfr401	−0.88657
		258353	Olfr521	−0.88221
		258290	Olfr1143	−0.84815
		258465	Olfr1387	−0.84763
		258310	Olfr145	−0.83906
		258064	Olfr316	−0.64194
		**258492**	**Olfr484**	**1.013283**
		**258879**	**Olfr330**	**1.320387**
	Signal transduction	259082	Olfr1062	−3.1017
		20750	Spp1	−2.47243
		16333	Ins1	−2.1446
		258674	Olfr1427	−2.1351
		258025	Olfr1211	−1.97936
		18365	Olfr65	−1.93793
		116903	Calcb	−1.87798
		258409	Olfr1431	−1.85304
		14459	Gast	−1.77078
		259071	Olfr166	−1.67436
		258880	Olfr218	−1.65817
		545481	Arhgap40	−1.55664
		258371	Olfr368	−1.53827
		258177	Olfr1222	−1.53601
		258784	Olfr1245	−1.52883
		18315	Olfr18	−1.44533
		258616	Olfr357	−1.44533
		80885	Hcar2	−1.4179
		258560	Olfr843	−1.4106
		258494	Olfr318	−1.36443
		258207	Olfr452	−1.35668
		574417	Tas2r137	−1.33149
		22422	Wnt7b	−1.2964
		258730	Olfr483	−1.24945
		404222	Olfr231	−1.24083
		258417	Olfr470	−1.23992
		258939	Olfr63	−1.23907
		258830	Olfr103	−1.16458
		258808	Olfr620	−1.15739
		259041	Olfr1414	−1.12738
		257902	Olfr704	−1.09448
		226970	Arhgef4	−1.07033
		404318	Olfr681	−1.02977
		13645	Egf	−1.01864
		14602	Ghrhr	−0.99429
		241113	Prkag3	−0.99043
		252837	Ackr4	−0.97382
		14686	Gnat2	−0.96149
		11549	Adra1a	−0.95816
		12776	Ccr8	−0.9491
		18314	Olfr17	−0.91665
		228543	Rhov	−0.90042
		258701	Olfr401	−0.88657
		258353	Olfr521	−0.88221
		228775	Trib3	−0.87301
		258290	Olfr1143	−0.84815
		258465	Olfr1387	−0.84763
		99296	Hrh3	−0.84015
		258310	Olfr145	−0.83906
		12922	Crhr2	−0.83292
		54199	Ccrl2	−0.80972
		22410	Wnt10b	−0.77983
		235611	Plxnb1	−0.74232
		107477	Guca1b	−0.72256
		65086	Lpar3	−0.70333
		224143	Poglut1	−0.67618
		11304	Abca4	−0.66413
		16971	Lrp1	−0.65473
		258064	Olfr316	−0.64194
		242093	Rxfp4	−0.63316
		13982	Esr1	−0.60031
		**26965**	**Cul1**	**0.587056**
		**21372**	**Tbl1x**	**0.594507**
		**18554**	**Pcsk7**	**0.596425**
		**16337**	**Insr**	**0.602833**
		**11789**	**Apc**	**0.608252**
		**54611**	**Pde3a**	**0.612094**
		**64654**	**Fgf23**	**0.61382**
		**20779**	**Src**	**0.618214**
		**102626**	**Mapkapk3**	**0.621833**
		**78757**	**Rictor**	**0.632871**
		**213498**	**Arhgef11**	**0.639783**
		**14367**	**Fzd5**	**0.653371**
		**54126**	**Arhgef7**	**0.671002**
		**18753**	**Prkcd**	**0.684102**
		**66313**	**Smurf2**	**0.696636**
		**18576**	**Pde3b**	**0.699416**
		**208643**	**Eif4g1**	**0.706288**
		**238871**	**Pde4d**	**0.709113**
		**18708**	**Pik3r1**	**0.711339**
		**17311**	**Kitl**	**0.738123**
		**20482**	**Skil**	**0.743497**
		**19730**	**Ralgds**	**0.760822**
		**11652**	**Akt2**	**0.802731**
		**12168**	**Bmpr2**	**0.808147**
		**13618**	**Ednrb**	**0.846174**
		**21337**	**Tacr2**	**0.860064**
		**258492**	**Olfr484**	**1.013283**
		**19206**	**Ptch1**	**1.232834**
		**258879**	**Olfr330**	**1.320387**
	GPCR downstream signalling	259082	Olfr1062	−3.1017
		258674	Olfr1427	−2.1351
		258025	Olfr1211	−1.97936
		18365	Olfr65	−1.93793
		116903	Calcb	−1.87798
		258409	Olfr1431	−1.85304
		14459	Gast	−1.77078
		259071	Olfr166	−1.67436
		258880	Olfr218	−1.65817
		258371	Olfr368	−1.53827
		258177	Olfr1222	−1.53601
		258784	Olfr1245	−1.52883
		18315	Olfr18	−1.44533
		258616	Olfr357	−1.44533
		80885	Hcar2	−1.4179
		258560	Olfr843	−1.4106
		258494	Olfr318	−1.36443
		258207	Olfr452	−1.35668
		574417	Tas2r137	−1.33149
		258730	Olfr483	−1.24945
		404222	Olfr231	−1.24083
		258417	Olfr470	−1.23992
		258939	Olfr63	−1.23907
		258830	Olfr103	−1.16458
		258808	Olfr620	−1.15739
		259041	Olfr1414	−1.12738
		257902	Olfr704	−1.09448
		226970	Arhgef4	−1.07033
		404318	Olfr681	−1.02977
		14602	Ghrhr	−0.99429
		14686	Gnat2	−0.96149
		11549	Adra1a	−0.95816
		12776	Ccr8	−0.9491
		18314	Olfr17	−0.91665
		258701	Olfr401	−0.88657
		258353	Olfr521	−0.88221
		258290	Olfr1143	−0.84815
		258465	Olfr1387	−0.84763
		99296	Hrh3	−0.84015
		258310	Olfr145	−0.83906
		12922	Crhr2	−0.83292
		235611	Plxnb1	−0.74232
		65086	Lpar3	−0.70333
		258064	Olfr316	−0.64194
		242093	Rxfp4	−0.63316
		**54611**	**Pde3a**	**0.612094**
		**213498**	**Arhgef11**	**0.639783**
		**54126**	**Arhgef7**	**0.671002**
		**18753**	**Prkcd**	**0.684102**
		**18576**	**Pde3b**	**0.699416**
		**238871**	**Pde4d**	**0.709113**
		**18708**	**Pik3r1**	**0.711339**
		**13618**	**Ednrb**	**0.846174**
		**21337**	**Tacr2**	**0.860064**
		**258492**	**Olfr484**	**1.013283**
		**258879**	**Olfr330**	**1.320387**
	Signalling by GPCR	259082	Olfr1062	−3.1017
		258674	Olfr1427	−2.1351
		258025	Olfr1211	−1.97936
		18365	Olfr65	−1.93793
		116903	Calcb	−1.87798
		258409	Olfr1431	−1.85304
		14459	Gast	−1.77078
		259071	Olfr166	−1.67436
		258880	Olfr218	−1.65817
		258371	Olfr368	−1.53827
		258177	Olfr1222	−1.53601
		258784	Olfr1245	−1.52883
		18315	Olfr18	−1.44533
		258616	Olfr357	−1.44533
		80885	Hcar2	−1.4179
		258560	Olfr843	−1.4106
		258494	Olfr318	−1.36443
		258207	Olfr452	−1.35668
		574417	Tas2r137	−1.33149
		258730	Olfr483	−1.24945
		404222	Olfr231	−1.24083
		258417	Olfr470	−1.23992
		258939	Olfr63	−1.23907
		258830	Olfr103	−1.16458
		258808	Olfr620	−1.15739
		259041	Olfr1414	−1.12738
		257902	Olfr704	−1.09448
		226970	Arhgef4	−1.07033
		404318	Olfr681	−1.02977
		14602	Ghrhr	−0.99429
		252837	Ackr4	−0.97382
		14686	Gnat2	−0.96149
		11549	Adra1a	−0.95816
		12776	Ccr8	−0.9491
		18314	Olfr17	−0.91665
		258701	Olfr401	−0.88657
		258353	Olfr521	−0.88221
		258290	Olfr1143	−0.84815
		258465	Olfr1387	−0.84763
		99296	Hrh3	−0.84015
		258310	Olfr145	−0.83906
		12922	Crhr2	−0.83292
		54199	Ccrl2	−0.80972
		235611	Plxnb1	−0.74232
		65086	Lpar3	−0.70333
		258064	Olfr316	−0.64194
		242093	Rxfp4	−0.63316
		**54611**	**Pde3a**	**0.612094**
		**213498**	**Arhgef11**	**0.639783**
		**14367**	**Fzd5**	**0.653371**
		**54126**	**Arhgef7**	**0.671002**
		**18753**	**Prkcd**	**0.684102**
		**18576**	**Pde3b**	**0.699416**
		**238871**	**Pde4d**	**0.709113**
		**18708**	**Pik3r1**	**0.711339**
		**13618**	**Ednrb**	**0.846174**
		**21337**	**Tacr2**	**0.860064**
		**258492**	**Olfr484**	**1.013283**
		**19206**	**Ptch1**	**1.232834**
		**258879**	**Olfr330**	**1.320387**
	Odorant GPCRs	113847	Vmn1r43	−1.70758
		113846	Vmn1r47	−1.51436
		18315	Olfr18	−1.44533
		258417	Olfr470	−1.23992
		258939	Olfr63	−1.23907
		113845	Vmn1r48	−1.049
		14788	Gpr162	−0.90054
		258310	Olfr145	−0.83906
		381628	Adgrf3	−0.81046
		224792	Adgrf5	−0.63765
		**233670**	**Olfr6**	**0.93882**
		**107515**	**Lgr4**	**0.968492**
		**258492**	**Olfr484**	**1.013283**
		**22296**	**Vmn1r51**	**1.806044**
	GPCRs, Other	18359	Olfr59	−2.08316
		258326	Olfr642	−1.88474
		113847	Vmn1r43	−1.70758
		113846	Vmn1r47	−1.51436
		18315	Olfr18	−1.44533
		113845	Vmn1r48	−1.049
		14788	Gpr162	−0.90054
		29848	Olfr29-ps1	−0.88445
		**14367**	**Fzd5**	**0.653371**
		**233670**	**Olfr6**	**0.93882**
		**22296**	**Vmn1r51**	**1.806044**
	IRS-related events	16333	Ins1	−2.1446
		241113	Prkag3	−0.99043
		228775	Trib3	−0.87301
		**16337**	**Insr**	**0.602833**
		**64654**	**Fgf23**	**0.61382**
		**18576**	**Pde3b**	**0.699416**
		**208643**	**Eif4g1**	**0.706288**
		**18708**	**Pik3r1**	**0.711339**
		**11652**	**Akt2**	**0.802731**
	Insulin receptor signalling cascade	16333	Ins1	−2.1446
		241113	Prkag3	−0.99043
		228775	Trib3	−0.87301
		**16337**	**Insr**	**0.602833**
		**64654**	**Fgf23**	**0.61382**
		**18576**	**Pde3b**	**0.699416**
		**208643**	**Eif4g1**	**0.706288**
		**18708**	**Pik3r1**	**0.711339**
		**11652**	**Akt2**	**0.802731**
	SHC activation	16333	Ins1	−2.1446
		**16337**	**Insr**	**0.602833**
Cancer signalling	Cellular Senescence	12608	Cebpb	−0.79497
		13619	Phc1	−0.62828
		**22059**	**Trp53**	**0.607334**
		**104248**	**Cabin1**	**0.613774**
		**102626**	**Mapkapk3**	**0.621833**
		**54383**	**Phc2**	**0.702052**
		**320332**	**Hist4h4**	**0.738735**
		**15260**	**Hira**	**0.740336**
		**97122**	**Hist2h4**	**0.763623**
		**319160**	**Hist1h4k**	**0.876777**
		**319148**	**Hist1h3c**	**0.96292**
	Basal cell carcinoma	93897	Fzd10	−1.96894
		22422	Wnt7b	−1.2964
		22410	Wnt10b	−0.77983
		**22059**	**Trp53**	**0.607334**
		**11789**	**Apc**	**0.608252**
		**14367**	**Fzd5**	**0.653371**
		**19206**	**Ptch1**	**1.232834**
	p38MAPK events	**20779**	**Src**	**0.618214**
		**102626**	**Mapkapk3**	**0.621833**
		**19730**	**Ralgds**	**0.760822**
	Activation of PKB	228775	Trib3	−0.87301
		**11652**	**Akt2**	**0.802731**
	Formation of Senescence-Associated Heterochromatin Foci (SAHF)	**104248**	**Cabin1**	**0.613774**
		**15260**	**Hira**	**0.740336**
Cell cycle	Meiosis	382522	Hist3h2bb-ps	−1.45034
		**320332**	**Hist4h4**	**0.738735**
		**97122**	**Hist2h4**	**0.763623**
		**319160**	**Hist1h4k**	**0.876777**
		**319148**	**Hist1h3c**	**0.96292**
		**20962**	**Sycp3**	**1.533723**
	Meiotic Recombination	**320332**	**Hist4h4**	**0.738735**
		**97122**	**Hist2h4**	**0.763623**
		**319160**	**Hist1h4k**	**0.876777**
		**319148**	**Hist1h3c**	**0.96292**
	RNA Polymerase I Promoter Opening	**320332**	**Hist4h4**	**0.738735**
		**97122**	**Hist2h4**	**0.763623**
		**319160**	**Hist1h4k**	**0.876777**
		**319148**	**Hist1h3c**	**0.96292**
Oxidative stress	Oxidative Stress Induced Senescence	13619	Phc1	−0.62828
		**22059**	**Trp53**	**0.607334**
		**102626**	**Mapkapk3**	**0.621833**
		**54383**	**Phc2**	**0.702052**
		**320332**	**Hist4h4**	**0.738735**
		**97122**	**Hist2h4**	**0.763623**
		**319160**	**Hist1h4k**	**0.876777**
		**319148**	**Hist1h3c**	**0.96292**
	Cellular responses to stress	12608	Cebpb	−0.79497
		13619	Phc1	−0.62828
		**22059**	**Trp53**	**0.607334**
		**104248**	**Cabin1**	**0.613774**
		**102626**	**Mapkapk3**	**0.621833**
		**54383**	**Phc2**	**0.702052**
		**320332**	**Hist4h4**	**0.738735**
		**15260**	**Hira**	**0.740336**
		**97122**	**Hist2h4**	**0.763623**
		**319160**	**Hist1h4k**	**0.876777**
		**319148**	**Hist1h3c**	**0.96292**
Neuronal response	Amyloids	**320332**	**Hist4h4**	**0.738735**
		**97122**	**Hist2h4**	**0.763623**
		**319160**	**Hist1h4k**	**0.876777**
		**319148**	**Hist1h3c**	**0.96292**
	Meiotic Synapsis	382522	Hist3h2bb-ps	−1.45034
		**320332**	**Hist4h4**	**0.738735**
		**97122**	**Hist2h4**	**0.763623**
		**319160**	**Hist1h4k**	**0.876777**
		**319148**	**Hist1h3c**	**0.96292**
		**20962**	**Sycp3**	**1.533723**
Metabolism	Vitamin C (ascorbate) metabolism	68214	Gsto2	−0.79791
		**109754**	**Cyb5r3**	**0.61693**
		**54338**	**Slc23a2**	**0.678156**
	Protein citrullination	18599	Padi1	−1.40412
		18600	Padi2	−0.92956

**Table 7:** Group of pathways, pathways, genes related to this pathways and log2FC values after 14 days of exposure to E171 in BALB/c mice. Numbers in bold are upregulated genes. Log2FC=Log2 fold change obtained with LIMMA script with correction for its own time-matched control.

**Group of pathways**	**Pathways**	**EntrezGeneID**	**Genes**	**Log2FC**
Cancer signalling	GAB1 signalosome	12524	Cd86	−1.08973
		18706	Pik3ca	−1.03111
		18595	Pdgfra	−0.79464
		18591	Pdgfb	−0.73059
		19211	Pten	−0.65603
		**18709**	**Pik3r2**	**0.590647**
		**67605**	**Akt1s1**	**0.61903**
		**18708**	**Pik3r1**	**0.668175**
		**64654**	**Fgf23**	**0.67401**
		**56484**	**Foxo3**	**0.778968**
		**384783**	**Irs2**	**0.794769**
		**20779**	**Src**	**0.798342**
		**12575**	**Cdkn1a**	**0.821051**
	PIP3 activates AKT signalling PI-3K cascade PI3K events in ERBB2 signalling PI3K/AKT Signalling in Cancer PI3K/AKT activation Constitutive PI3K/AKT Signalling in Cancer	12524	Cd86	−1.08973
		18706	Pik3ca	−1.03111
		18595	Pdgfra	−0.79464
		18591	Pdgfb	−0.73059
		19211	Pten	−0.65603
		**18709**	**Pik3r2**	**0.590647**
		**67605**	**Akt1s1**	**0.61903**
		**18708**	**Pik3r1**	**0.668175**
		**64654**	**Fgf23**	**0.67401**
		**56484**	**Foxo3**	**0.778968**
		**384783**	**Irs2**	**0.794769**
		**12575**	**Cdkn1a**	**0.821051**
	PI3K events in ERBB4 signalling	12524	Cd86	−1.08973
		18706	Pik3ca	−1.03111
		68318	Aph1c	−0.84754
		18595	Pdgfra	−0.79464
		18591	Pdgfb	−0.73059
		17999	Nedd4	−0.68405
		13982	Esr1	−0.68068
		19211	Pten	−0.65603
		80707	Wwox	−0.64542
		**18709**	**Pik3r2**	**0.590647**
		**67605**	**Akt1s1**	**0.61903**
		**18708**	**Pik3r1**	**0.668175**
		**64654**	**Fgf23**	**0.67401**
		**56484**	**Foxo3**	**0.778968**
		**384783**	**Irs2**	**0.794769**
		**12575**	**Cdkn1a**	**0.821051**
	Glioma	18706	Pik3ca	−1.03111
		18595	Pdgfra	−0.79464
		18591	Pdgfb	−0.73059
		20663	Sos2	−0.65643
		19211	Pten	−0.65603
		**18709**	**Pik3r2**	**0.590647**
		**18708**	**Pik3r1**	**0.668175**
		**12575**	**Cdkn1a**	**0.821051**
		**22059**	**Trp53**	**0.876668**
		**11652**	**Akt2**	**1.261177**
	Melanoma	18706	Pik3ca	−1.03111
		18595	Pdgfra	−0.79464
		18591	Pdgfb	−0.73059
		19211	Pten	−0.65603
		**18709**	**Pik3r2**	**0.590647**
		**18708**	**Pik3r1**	**0.668175**
		**64654**	**Fgf23**	**0.67401**
		**12575**	**Cdkn1a**	**0.821051**
		**22059**	**Trp53**	**0.876668**
		**11652**	**Akt2**	**1.261177**
Cell cycle	Caspase-mediated cleavage of cytoskeletal proteins	14453	Gas2	−0.77701
		17762	Mapt	−0.77412
		**18810**	**Plec**	**0.588317**
		**13169**	**Dbnl**	**0.660419**
	Signalling by ERBB4	12524	Cd86	−1.08973
		18706	Pik3ca	−1.03111
		68318	Aph1c	−0.84754
		18595	Pdgfra	−0.79464
		18591	Pdgfb	−0.73059
		17999	Nedd4	−0.68405
		13982	Esr1	−0.68068
		19211	Pten	−0.65603
		80707	Wwox	−0.64542
		**18709**	**Pik3r2**	**0.590647**
		**67605**	**Akt1s1**	**0.61903**
		**18708**	**Pik3r1**	**0.668175**
		**64654**	**Fgf23**	**0.67401**
		**56484**	**Foxo3**	**0.778968**
		**384783**	**Irs2**	**0.794769**
		**12575**	**Cdkn1a**	**0.821051**
	PERK regulated gene expression	13666	Eif2ak3	−0.68318
		13665	Eif2s1	−0.63654
Oxidative stress	Mitochondrial Uncoupling Proteins The fatty acid cycling model The proton buffering model	**22228**	**Ucp2**	**0.870627**
		**22229**	**Ucp3**	**0.784513**
Immune response	Role of LAT2/NTAL/LAB on calcium mobilization	12524	Cd86	−1.08973
		18706	Pik3ca	−1.03111
		18595	Pdgfra	−0.79464
		18591	Pdgfb	−0.73059
		19211	Pten	−0.65603
		56743	Lat2	−0.59939
		**18709**	**Pik3r2**	**0.590647**
		**67605**	**Akt1s1**	**0.61903**
		**18708**	**Pik3r1**	**0.668175**
		**64654**	**Fgf23**	**0.67401**
		**56484**	**Foxo3**	**0.778968**
		**384783**	**Irs2**	**0.794769**
		**12575**	**Cdkn1a**	**0.821051**
	Chemokine signalling pathway	20302	Ccl3	−2.21765
		12772	Ccr2	−1.20079
		20306	Ccl7	−1.10314
		18706	Pik3ca	−1.03111
		14772	Grk4	−1.02692
		15162	Hck	−1.01759
		140580	Elmo1	−0.99056
		80901	Cxcr6	−0.90825
		20293	Ccl12	−0.84754
		277360	Prex1	−0.76644
		20301	Ccl27a	−0.74211
		11513	Adcy7	−0.68104
		20663	Sos2	−0.65643
		57257	Vav3	−0.62284
		216869	Arrb2	−0.61054
		**18709**	**Pik3r2**	**0.590647**
		**18708**	**Pik3r1**	**0.668175**
		**56484**	**Foxo3**	**0.778968**
		**20779**	**Src**	**0.798342**
		**11652**	**Akt2**	**1.261177**
	Role of phospholipids in phagocytosis	18706	Pik3ca	−1.03111
		14129	Fcgr1	−0.82062
		14130	Fcgr2b	−0.78621
		**18709**	**Pik3r2**	**0.590647**
		**18708**	**Pik3r1**	**0.668175**
Bone development	Chondroitin sulfate biosynthesis	121021	Cspg4	−0.93035
		12111	Bgn	−0.78843
		60322	Chst7	−0.78254
		100910	Chpf2	−0.73774
		269941	Chsy1	−0.67983
	Chondroitin sulfate/dermatan sulfate metabolism	338362	Ust	−1.31467
		121021	Cspg4	−0.93035
		12111	Bgn	−0.78843
		60322	Chst7	−0.78254
		71951	Gpc2	−0.78001
		100910	Chpf2	−0.73774
		269941	Chsy1	−0.67983
		**15932**	**Idua**	**0.700167**
Neuronal response	Serotonin receptors	15566	Htr7	−1.20957
		15551	Htr1b	−1.07636
		**15565**	**Htr6**	**1.237737**

**Table 8:** Group of pathways, pathways, genes related to this pathways and log2FC values after 21 days of exposure to E171 in BALB/c mice. Numbers in bold are upregulated genes. Log2FC=Log2 fold change obtained with LIMMA script with correction for its own time-matched control.

**Group of pathways**	**Pathways**	**EntrezGeneID**	**Genes**	**Log2FC**
Signalling	Olfactory Signalling Pathway	258267	Olfr370	−1.87677
		258882	Olfr874	−1.30081
		**258064**	**Olfr316**	**0.697396**
		**634104**	**Olfr287**	**0.724416**
		**259145**	**Olfr1251**	**0.777263**
		**258269**	**Olfr930**	**0.853172**
		**258701**	**Olfr401**	**1.156804**
		**258358**	**Olfr557**	**2.218149**
	Olfactory transduction	258267	Olfr370	−1.87677
		258543	Olfr810	−1.7235
		258882	Olfr874	−1.30081
		258238	Olfr417	−0.99523
		**57269**	**Olfr1507**	**0.696378**
		**634104**	**Olfr287**	**0.724416**
		**258269**	**Olfr930**	**0.853172**
		**259114**	**Olfr570**	**1.00058**
		**258701**	**Olfr401**	**1.156804**
		**18345**	**Olfr46**	**1.302175**
		**258358**	**Olfr557**	**2.218149**
	GPCR downstream signalling	258267	Olfr370	−1.87677
		15945	Cxcl10	−1.61387
		258882	Olfr874	−1.30081
		15551	Htr1b	−0.89351
		20301	Ccl27a	−0.59555
		**258064**	**Olfr316**	**0.697396**
		**634104**	**Olfr287**	**0.724416**
		**259145**	**Olfr1251**	**0.777263**
		**258269**	**Olfr930**	**0.853172**
		**258701**	**Olfr401**	**1.156804**
		**57385**	**P2ry4**	**1.273995**
		**387345**	**Tas2r113**	**1.357752**
		**258358**	**Olfr557**	**2.218149**
	Signal Transduction	258267	Olfr370	−1.87677
		15945	Cxcl10	−1.61387
		258882	Olfr874	−1.30081
		15551	Htr1b	−0.89351
		228775	Trib3	−0.8798
		20301	Ccl27a	−0.59555
		50768	Dlc1	−0.588
		**20779**	**Src**	**0.585707**
		**81897**	**Tlr9**	**0.614653**
		**16846**	**Lep**	**0.653292**
		**16835**	**Ldlr**	**0.687404**
		**107971**	**Frs3**	**0.690578**
		**258064**	**Olfr316**	**0.697396**
		**634104**	**Olfr287**	**0.724416**
		**259145**	**Olfr1251**	**0.777263**
		**12575**	**Cdkn1a**	**0.818227**
		**258269**	**Olfr930**	**0.853172**
		**258701**	**Olfr401**	**1.156804**
	Signalling by GPCR	258267	Olfr370	−1.87677
		15945	Cxcl10	−1.61387
		258882	Olfr874	−1.30081
		15551	Htr1b	−0.89351
		20301	Ccl27a	−0.59555
		**258064**	**Olfr316**	**0.697396**
		**634104**	**Olfr287**	**0.724416**
		**259145**	**Olfr1251**	**0.777263**
		**258269**	**Olfr930**	**0.853172**
		**258701**	**Olfr401**	**1.156804**
		**57385**	**P2ry4**	**1.273995**
		**387345**	**Tas2r113**	**1.357752**
		**258358**	**Olfr557**	**2.218149**
Oxidative stress	– Mitochondrial Uncoupling Proteins – The fatty acid cycling model – The proton buffering model	**22228**	**Ucp2**	**0.595733**
		**22229**	**Ucp3**	**0.598441**
Metabolism	Synthesis of glycosylphosphatidylinositol (GPI)	**14755**	**Pigq**	**0.59899**
		**55981**	**Pigb**	**0.662836**

## 2 Experimental design, materials and methods

### 2.1 Mouse model

Thirty-two BALB/c mice (16 males, 16 females) of 4–6 weeks old (Harlan Laboratories, Mexico) underwent exposure to E171 exposure after ethical approval from the Comité de Ética de la Facultad de Estudios Superiores Iztacala de la Universidad Nacional Autónoma de México under the number: FESI-ICY-I151. They were housed in polycarbonate cages and kept in a housing room (12 h light/dark cycles, 50–60% relative humidity, air filtered until 5 µm particles and exchanged 18 times/h, and 21 °C). After one week of acclimation, the mice were randomly divided in 2 groups: the control group (8 males, 8 females) was exposed by gavage to sonicated sterile water (30 min 60 Hz), and the exposure group (8 males, 8 females) was exposed to 5 mg/kg bw/day of sonicated sterile E171 (30 min 60 Hz). Four mice (2 males, 2 females) were sacrificed in a humid chamber with sevoflurane after 2, 7, 14 and 21 days. Colons were collected and put overnight at 4 °C in a tube containing RNAlater® (Thermofischer, The Netherlands). Next day the remaining RNAlater® was discarded. The colons were stored at −80 °C until RNA isolation. Transportation of the samples for 2 days in dry ice at −80 °C has been done by a specialised shipping company to the Department of Toxicogenomics, Maastricht University, Maastricht, the Netherlands. The shipping company monitored the temperature of the samples throughout the shipping process with a thermometer to ensure stable freezing conditions and optimal sample quality. Immediately upon arrival the samples have been put at −80 °C.

### 2.2 RNA isolation

Total RNA was isolated from the distal part of colon. Colons were first submerged in Qiazol (Qiagen, The Netherlands) and subsequently disrupted and homogenized using a Mini Bead Beater (BioSpec Products, The Netherlands) (48 beats/s for 30 s). miRNeasy Mini Kit was used for RNA isolation (Qiagen, The Netherlands) including a DNase treatment, according to the manufacturer's protocol [4]. RNA concentrations were measured using a Nanodrop® spectrophotometer (Thermofischer, The Netherlands) and the integrity of total RNA was determined on a Bioanalyzer (Agilent Technologies, The Netherlands). RNA Integrity Number (RIN) higher than 6 was compulsory for each sample to be used for microarray analysis. It was the case for all samples with an average of 8.8±0.7.

### 2.3 Microarray preparation and data pre-processing

Samples were labelled with Cyanine 3 (Cy 3), hybridized, and washed according to the One-Color Microarray-Based Gene Expression Analysis protocol version 6.6 (Agilent Technologies, The Netherlands) [5]. Hybridization was performed on Agilent SurePrint G3 mouse Gene exp 60kv2 microarrays. Samples were scanned using an Agilent DNA Microarray Scanner with Surescan High-resolution Technology (Agilent Technologies, The Netherlands). Raw data was extracted and checked by the quality control pipeline provided by Agilent (Feature extraction software (FES) version 10.7.3.1). Another quality check was performed with an in-house pipeline in R (https://github.com/BiGCAT-UM/arrayQC_Module/) and data was normalized with local background correction, flagging of bad spots, controls and spots with too low intensity, log2 transformation and quantile normalization. Raw data with expression values and genes were selected for data analysis based on flags and missing values (GEO accession: GSE92563). Height groups were defined: control 2 days, 7 days, 14 days, 21 days for the controls and E171 2 days, 7 days, 14 days, 21 days for the exposed samples. Within each group, unique identifiers had to pass for the spot and have less than 40% of missing values. In all groups, unique identifiers had to have an average expression >4, missing values were imputed by k-nearest neighbours (k-NN; k-value 15), and repeated identifiers merged on the median. Differentially expressed genes were extracted with a Linear Mixed Model Analysis for Microarrays (LIMMA) [6] (version 1.0) [7] with the following criteria: a fold-change (FC) of 1.5 and a p-value of 0.05. The data of each control time point (control) was corrected from the time-matched exposed mice to E171. DEG extracted are shown in Table 1, Table 2, Table 3, Table 4.

### 2.4 Pathway analysis

Functional annotation of these treatment-specific DEG was performed with Consensus Pathway Database (CPDB) for an over-representation gene set analysis (ORA) [8], [9]. All the available databases from CPDB were used (release MM9, 11 Oct. 2013) with settings in the “pathways as defined by pathway databases” with a minimum overlap of input list of 2 and a *p*-value cut-off of *p*<0.01. For each annotation set, the p-value is calculated using a Fisher's exact test. CPBD corrects for multiple hypothesis testing using the false discovery rate procedure within each type of annotation set [8], [9]. Results of the ORA are available in Table 5, Table 6, Table 7, Table 8.
